# *N*-Derivatives of (*Z*)-Methyl 3-(4-Oxo-2-thioxothiazolidin-5-ylidene)methyl)-1*H*-indole-2-carboxylates as Antimicrobial Agents—In Silico and In Vitro Evaluation

**DOI:** 10.3390/ph16010131

**Published:** 2023-01-16

**Authors:** Anthi Petrou, Athina Geronikaki, Victor Kartsev, Antonios Kousaxidis, Aliki Papadimitriou-Tsantarliotou, Marina Kostic, Marija Ivanov, Marina Sokovic, Ioannis Nicolaou, Ioannis S. Vizirianakis

**Affiliations:** 1 Department of Pharmaceutical Chemistry, School of Pharmacy, Aristotle University of Thessaloniki, 54124 Thessaloniki, Greece; 2 InterBioScreen, 142432 Chernogolovka, Russia; 3 Laboratory of Pharmacology, School of Pharmacy, Aristotle University of Thessaloniki, 54124 Thessaloniki, Greece; 4 Mycological Laboratory, Department of Plant Physiology, Institute for Biological Research “Siniša Stankovic”—National Institute of Republic of Serbia, University of Belgrade, Bulevar Despota Stefana 142, 11000 Belgrade, Serbia; 5 Department of Life and Health Sciences, University of Nicosia, Nicosia 2417, Cyprus

**Keywords:** indole, rhodanine, antimicrobial, antibacterial activity, antifungal activity, molecular docking, ADMET, cytotoxicity

## Abstract

Herein, we report the experimental evaluation of the antimicrobial activity of seventeen new (*Z*)-methyl 3-(4-oxo-2-thioxothiazolidin-5-ylidene)methyl)-1*H*-indole-2-carboxylate derivatives. All tested compounds exhibited antibacterial activity against eight Gram-positive and Gram-negative bacteria. Their activity exceeded those of ampicillin as well as streptomycin by 10–50 fold. The most sensitive bacterium was *En. Cloacae*, while *E. coli* was the most resistant one, followed by *M. flavus.* The most active compound appeared to be compound **8** with MIC at 0.004–0.03 mg/mL and MBC at 0.008–0.06 mg/mL. The antifungal activity of tested compounds was good to excellent with MIC in the range of 0.004–0.06 mg/mL, with compound **15** being the most potent. *T. viride* was the most sensitive fungal, while *A. fumigatus* was the most resistant one. Docking studies revealed that the inhibition of *E. coli* MurB is probably responsible for their antibacterial activity, while 14a–lanosterol demethylase of CYP51Ca is involved in the mechanism of antifungal activity. Furthermore, drug-likeness and ADMET profile prediction were performed. Finally, the cytotoxicity studies were performed for the most active compounds using MTT assay against normal MRC5 cells.

## 1. Introduction

Infectious diseases symbolize a consequential global strain on public health security and impact socio-economic stability all over the world. They have monopolized for centuries the dominant factors of death and disability of millions of humans.

Since their discovery, antimicrobial agents have been a reliable weapon in fighting life-threatening infections. Despite the availability of effective antibiotics for the most common infections, the emergence of multidrug-resistant bacteria pathogens and the spread of new infectious diseases threaten to weaken the efficiency of the drugs currently approved for infectious disease therapy [1,2].

On the other hand, infections evoked by several pathogenic fungi species result in 1.5 million deaths worldwide every year [3]. The therapeutic approach to these diseases is still challenging due to the continuous increase of resistance to antifungal drugs in sufferers, especially in immuno-suppressed patients (cancer, transplants, HIV) and those who are frequently treated with antimitotic therapy. Therefore, there is an urgent necessity to put efforts into discovering novel agents against invasive microbial infections.

**Figure 1:**
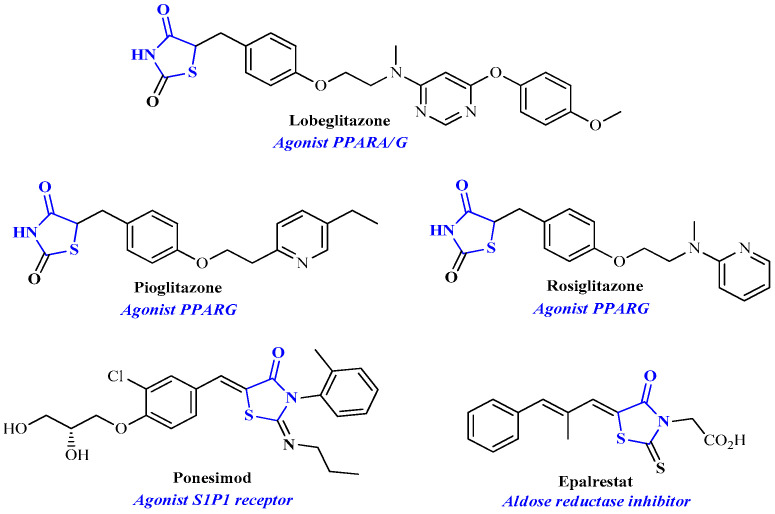
Structures of approved 4-thiazolidinone-based drugs.

Aromatic heterocycles have been used as structural scaffolds to obtain a variety of bioactive compounds possessing antibacterial, antifungal, antiviral, and anti-parasitic activities [4]. Over the past decades, thiazolidine-2,4-diones (glitazones) have emerged as the most successful member of the 4-thiazolidinone compounds [5]. There are several drugs from this class of heterocycles, such as Lobeglitazone, Pioglitazone, Rosiglitazone, and Epalrestat, used broadly as oral anti-hyperglycemic agents for diabetes mellitus type 2 therapies, and recently, Ponesimod was approved for healing multiple sclerosis and psoriasis (Figure 1).

In addition, 2 hodamine derivatives demonstrate a broad spectrum of pharmacological activities, such as antimicrobial [6,7,8,9], anticancer [10,11,12], anti-HIV [13,14], anti-diabetic [15,16], anti-tubercular [17], and immunoproteasome inhibitory activity [18], suggesting that this scaffold occupies a vital position in drug discovery.

**Figure 2:**
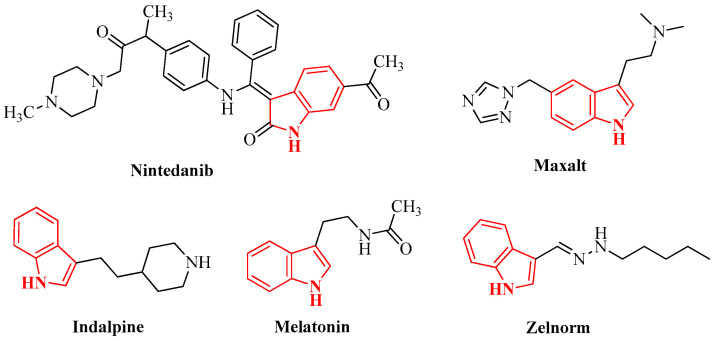
Structures of approved indole-based drugs.

Another promising scaffold is the indole ring attracting considerable attention because of its presence in proteins, amino acids, and bioactive alkaloids [19], and the wide range of biological activities of its derivatives [20,21,22,23,24,25,26,27,28,29,30,31,32,33,34,35], among which is antimicrobial activity [36,37,38,39,40,41]. Furthermore, indole ring is present in many approved drugs (Figure 2).

In recent years, the concept of designing hybrid molecules that contain two or more pharmacophore groups bound together covalently into one molecular framework is gaining ground. There are some publications regarding the importance of co-operative hydrogen bonding in antibiotics and the molecular hybridization approach of sugar-fused indoles [42,43].

Since the molecular hybridization approach may result in compounds that inhibit more than one target, we set up our investigation to discover novel antibacterial and antifungal agents based on this rationale [44].

**Figure 3:**
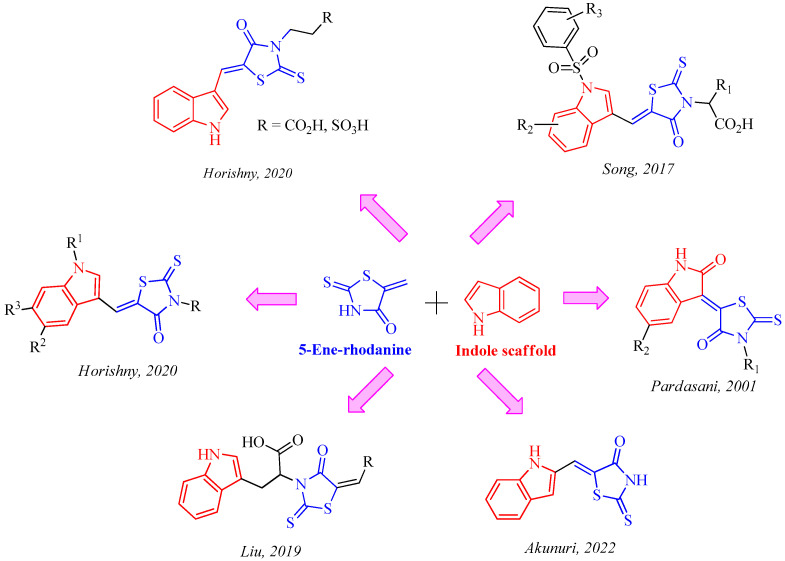
Antimicrobial agents containing indole and rhodanine ring [6,41,45,47,48,49].

So far, many indole-based rhodanine derivatives (Figure 3) have been proposed [6,41,45,46,47,48,49] as antimicrobial agents, some of which possess high efficacy in treating multi-drug resistant pathogens [6,41,47,48,49]. Therefore, it is noteworthy that the combination of indole and rhodanine scaffolds into new chemical entities could be a promising strategy for antimicrobial therapies. Inspired by this strategy, we have described in our previous paper [6,41] the design and synthesis of potential antimicrobial agents by incorporating indole and rhodanine moieties into new molecules. Herein, we report the synthesis, the evaluation of antimicrobial activity, molecular docking studies, and the prediction of pharmacokinetic and toxicity profiles of our compounds.

## 2. Results and Discussion

### 2.1. Prediction of Toxicity

The prediction of compound toxicities is a key part of the drug development pipeline. Toxicity assessments in silico are not only faster than the determination of toxic doses in animals but can also facilitate the lessening of the number of experiments in vivo. In our case, a computational toxicity study was carried out prior to the in vitro evaluation of compounds **1**–**17** with the aim to identify and remove potentially toxic structures. ProTox-II web server was used for the prediction of various toxicity endpoints, such as rat acute toxicity, hepatotoxicity, cytotoxicity, carcinogenicity, mutagenicity, immunotoxicity, and adverse outcome (Tox21) pathways (Tables S1 and S2). In addition, ADMET Predictor 10.4 (Simulation Plus) was used to investigate other critical toxicity issues such as phospholipidosis and hERG-related cardiotoxicity (Table S1).

It is known that toxic doses are often given as LD_50_ values in mg/kg body weight, where LD_50_ represents the median lethal dose upon exposure to a compound. Toxicity classes are defined according to the globally harmonized system of classification of the labeling of chemicals (GHS), that is, Class I: fatal if swallowed (LD50 ≤ 5); Class II: fatal if swallowed (5 < LD50 ≤ 50); Class III: toxic if swallowed (50 < LD50 ≤ 300); Class IV: harmful if swallowed (300 < LD50 ≤ 2000); Class V: may be harmful if swallowed (2000 < LD50 ≤ 5000); and Class VI: non-toxic (LD50 > 5000). In our preliminary investigation, it was estimated (54–66% accuracy) that all examined compounds are classified in category IV, indicating a promising profile towards oral rat acute toxicity. Furthermore, our compounds did not predict to demonstrate adverse outcomes (Table S2).

### 2.2. Chemistry

**Scheme 1:**
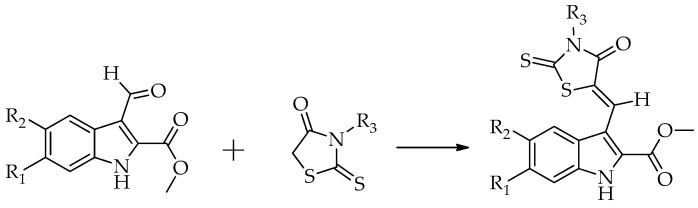
Preparation of compounds **1**–**17** Reagents and conditions: CH_3_COONH_4_, CH_3_COOH, reflux 2 h.

Compounds were synthesized according to the process described in detail in our previous paper [41] and presented in Scheme 1. The structures of all synthesized compounds are illustrated in Table 1 (Table S1) and confirmed by ^1^H and ^13^C NMR spectroscopy and elemental analysis, which are described in the experimental part.

### 2.3. Biological Evaluation

#### 2.3.1. Antimicrobial Activity

The synthesized compounds (Table 1) were evaluated for their antibacterial activity against a panel of eight species using a microdilution method with ampicillin and streptomycin as reference drugs. The antibacterial activity of tested compounds was good to very good with MIC/MBC values ranging from 0.004 mg/mL to 0.045 mg/mL and 0.008 mg/mL to 1.2 mg/mL, respectively, presented in Table 2. The order of activity can be presented as: **8** > **11** > **2** > **1** > **12** > **3** > **17** > **7** > **5** > **13** > **14** > **16** > **9** > **4** = **6** > **15** > **10**. The best activity was expressed by compound **8** with MIC at 0.004–0.03 mg/mL and MBC at 0.008–0.06 mg/mL, while compound **10** was the least active one. Compounds **1**, **2**, and **3** exhibited good activity against *B. cereus* with MIC at 0.015 mg/mL. The same good activity was observed for compounds **2**, **3**, **5**, **6**, and **7** against *S. aureus***; 8** and **12** against *L. monocytogenes*; and compounds **2**–**6** and **12** against *En. Cloacae*. Compound **12**, together with compounds **1**, **3**, **7**, and **11** (MIC 0.015 mg/mL), showed good activity against *S. typhimurium*, while derivatives **1** and **11** showed good activity against *E. coli*. On the other hand, compounds **8** and **12** showed excellent activity with MIC at 0.004 mg/mL against *En. cloacae* and *E. coli*, respectively. Very good activity (MIC 0.008 mg/mL) was observed for compounds **11** and **17** against *B. cereus* and *S. aureus*, respectively. At the same time, compound **11** exhibited good activity with MIC at 0.011 mg/mL against *En. cloacae* and *P. aeruginosa*. It should be mentioned that all compounds were more potent than both reference drugs against all bacteria tested. The most sensitive bacteria appeared to be *En. cloacae*, while *E. coli* was the most resistant one, followed by *M. flavus*.

The structure–activity relationship revealed that the presence of 3-methylbutanoic acid as a substituent on the nitrogen of the 2-thioxothiazolidin-4-one moiety of (*Z*)-2-(5-((5-fluoro-2-(methoxycarbonyl)-1*H*-indol-3-yl)methylene)-4-oxo-2-thioxothiazolidin-3-yl)-3-methylbutanoic acid, as well as methylformate in the indole ring (**8**), is beneficial for antibacterial activity. The replacement of 3-methylbutanoic acid by an acetic acid substituent on the nitrogen of 2-thioxothiazolidin-4-one ring, as well as the removal of the F-substituent at position 5 of the indole ring, decreased a little in activity (**11**), while the presence of benzoic acid at the same position led to a slightly less active compound **2**. The removal of F-substituent at position 5 on the indole ring from compound **8** led to compound **12**, which is fifth in the order of activity, while the presence of 4-hydroxybenzene substituent on the nitrogen of 2-thioxothiazolidin-4-one moiety appeared to be detrimental for antibacterial activity. Thus, the structure–activity relationship studies revealed that the activity of compounds depends not only on the substituent at the 2-thioxothiazolidin-4-one ring but as well as at the indole ring.

#### 2.3.2. Antifungal Activity

The antifungal activity of compounds was tested against eight fungal species using the drugs ketoconazole and bifonazole as reference. The antifungal activity of tested compounds was good to excellent with MIC in the range of 0.004–0.06 mg/mL and MFC at 0.008–0.12 mg/mL, presented in Table 3.

The order of activity of tested compounds can be presented as follows: **15** > **3** > **16** > **10** > **7** > **6** > **2** > **5** > **11** > **9** > **13** > **4** > **12** > **17** > **14** > **1** > **8.** Thus, the best activity was achieved by compound **15** with MIC/MFC at 0.008–0.015/0.015–0.03 mg/mL, respectively, while compound **8** was the least active. The opposite was observed in the case of antibacterial activity, where compound **8** was the most active and **15** was one of the least active. Some compounds showed excellent activity against some fungal species being more potent than both reference drugs. Thus, compound **1** expressed activity with MIC 0.004 mg/mL against *P. ochramensis*, while **3**, **5**, **7**, and **10** expressed activity against *T. viride.* At the same time, compounds **10** and **9** showed the same good activity against *A. ochraceus*. Most of the compounds demonstrated very good activity against *A. ochraceus* with MIC at 0.008 mg/mL (**1**, **2**, **4**–**8**, **11**), while compounds **4**, **6**, **9**, **11**, **12**, **15**, and **16** were also very good against *T. viride*. Furthermore, compounds **6**, **7**, **10**, and **15** were also very potent against *P. ochramensis*, whereas **3 7**, **10**, and **15** also demonstrated very good activity against *A. niger* and *P. funiculosum*, respectively. Finally, compound **15** appeared to be very active also against *P. cyclpoium var verucosum* after *A. fumigatus*, the most resistant fungi. *T. viride* was found to be the most sensitive one.

The structure–activity relationship studies revealed that the presence of the methyl group substituent on the nitrogen of 2-thioxothiazolidin-4-one (**15**) moiety of (*Z*)-methyl 5-fluoro-3-((3-methyl-4-oxo-2-thioxothiazolidin-5-ylidene)methyl)-1*H*-indole-2-carboxylate is favorable for antifungal activity. The replacement of the methyl group by 4-hydroxybenzene (**3**) and the removal of the F-substituent of the indole ring slightly decreased the activity. The introduction of morpholine as a substituent instead of methyl in compound **15** led to a less active compound (**16**), nevertheless being among the third most active compounds, while the removal of the fluorine atom from the indole ring resulted in compound **6** being sixth in order of activity.

Finally, the presence of 3-methylbutanoic acid as a substituent at the nitrogen of 2-thioxothiazolidin-4-one moiety (**8**) was detrimental to antifungal activity, while showing the best antibacterial activity among all tested compounds. Thus, the antifungal activity of compounds, as in the case of antibacterial, depends not only on substituent at 2-thioxothiazolidin-4-one but also at the indole ring as well.

### 2.4. Docking Studies

#### 2.4.1. Docking to Antibacterial Targets

In order to predict the possible mechanism of activity of synthesized compounds, docking studies in different targets were carried out. In this direction, for docking studies, we used enzymes responsible for the most common mechanisms of activity of antibacterial agents, such as *E. coli* DNA gyrase, thymidylate kinase, *E. coli* primase, and *E. coli* MurA and *E. coli* MurB enzymes.

A low Free Energy of Binding represents a strong binding of ligand to the enzyme. Taking this into account, docking studies revealed that the Free Energy of Binding of all tested compounds to *E. coli* DNA gyrase, thymidylate kinase, *E. coli* primase, and *E. coli* MurA enzymes was higher than that of *E. coli* MurB (−7.54–10.88 kcal/mol). Therefore, it may be suggested that the inhibition of *E. coli* MurB is probably the most suitable mechanism of action of the compounds where binding scores were consistent with biological activity (Table 4).

**Figure 4:**
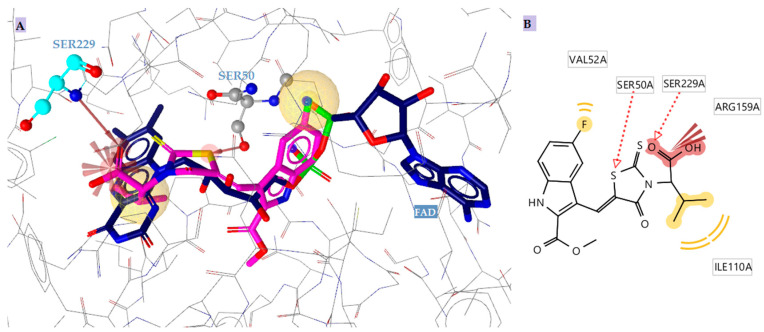
(**A**) Superposition of compound **8** (magenta) and **FAD** (blue) in *E. coli* MurB. (**B**) Docked conformation of the most active compound **8** in *E. coli* MurB. Red dotted arrows indicate H-bond and yellow spheres indicate hydrophobic interactions.

The docking pose of the most active compound **8** in the *E. coli* MurB enzyme showed two favorable hydrogen bond interactions. The first one is between the oxygen atom of the carbonyl group of the compound and the hydrogen of the side chain of Ser229 (distance 3.11 Å), and the other one is between the S atom of thiazolidinone moiety and Ser50 residue (distance 3.64 Å). Moreover, hydrophobic interactions between residues Val52, Arg159, Ile110, and the compound were detected, contributing to the stability of the complex ligand–enzyme (Figure 4). It is noteworthy to highlight that the hydrogen bond with the residue Ser229 is crucial for the inhibitory action of this compound because this residue takes part in the proton transfer at the second stage of peptidoglycan synthesis [50]. Hydrogen bond interactions with the residue Ser229 were also observed for most of the compounds (Table 4).

Moreover, detailed analysis of the docking pose of the most active compounds showed that they bind MurB in a similar way to FAD and they fit into the binding center of the enzyme in the same way, interacting with the same residues, such as Ser50, Arg213, Arg158, and Ser229 (Figure 4A). This is probably the reason why these compounds showed good inhibitory activity comparable to that of ampicillin.

#### 2.4.2. Docking to Antifungal Targets

All the synthesized compounds and the reference drug ketoconazole were docked to lanosterol 14α-demethylase of *C. albicans* and DNA topoisomerase IV (Table 5) in order to explore the possible mechanism of antifungal activity of compounds.

**Figure 5:**
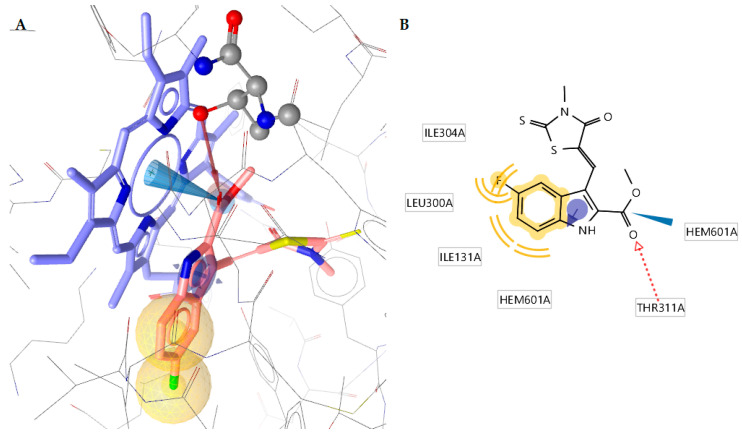
Docked conformation of the most active compound **15** in lanosterol 14α-demethylase of *C*. *albicans* (CYP51_ca_). (**A**) 3D representation of compound **15**. (**B**) 2D representation. Red dotted arrows indicate H-bond, blue arrows indicate aromatic interactions, and yellow spheres indicate hydrophobic interactions.

**Figure 6:**
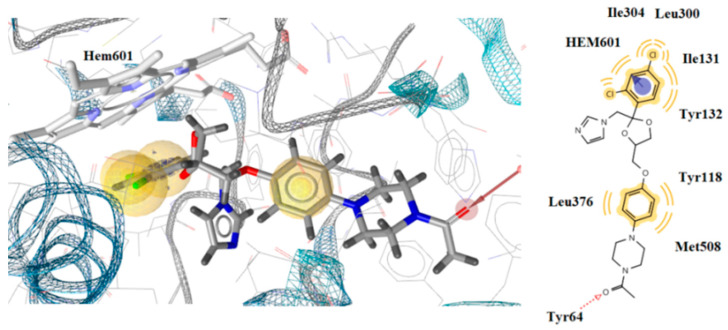
Docked conformation of ketoconazole in lanosterol 14α-demethylase of *C*. *albicans* (CYP51_ca_).

Docking studies showed that all compounds can bind the CYP51_Ca_ enzyme in an analogous mode to that of ketoconazole. Compound **15** is placed inside the enzyme by the side of the heme group, interacting hydrophobically aromatically and binding to the Fe of the heme. Furthermore, compound **15** forms one H-bond between the oxygen atom of the CO_2_ group and the side-chain hydrogen of Thr311. Hydrophobic interactions were detected between residues Ile304, Leu300, and Ile131 and the benzene ring of the compound **15** (Figure 5B). Interaction with the heme group was also observed with the benzene ring of ketoconazole, which forms aromatic and hydrophobic interactions (Figure 5). However, compound **15** formed a more stable complex of the ligand with the enzyme, indicating their interaction with the Fe of heme. This is probably the reason why compound **15** (Figure 5A) has better antifungal activity than ketoconazole (Figure 6).

### 2.5. Drug-Likeness and Bioavailability

**Figure 7:**
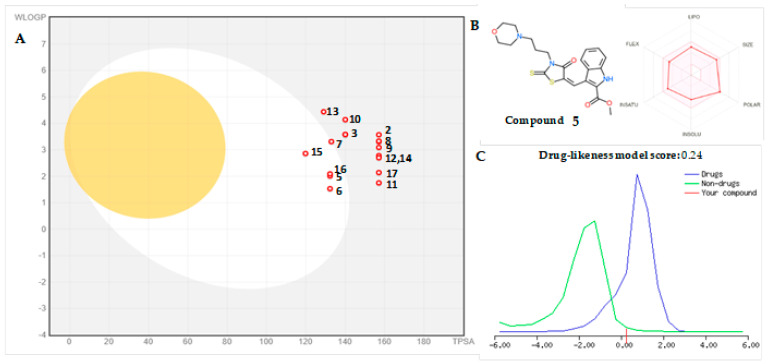
(**A**) Boiled egg, (**B**) bioavailability radar of compound **5**, (**C**) drug−likeness model. In bioavailability radar, the pink area represents the optimal range for each property for oral bioavailability, (Lipophilicity (LIPO): XLOGP3 between −0.7 and +5.0, Molecular weight (SIZE): MW between 150 and 500 g/mol, Polarity (POLAR) TPSA between 20 and 130 Å^2^, Solubility (INSOLU): log S not higher than 6, Saturation (INSATU): fraction of carbons in the sp^3^ hybridization not less than 0.25, and Flexibility (FLEX): no more than nine rotatable bonds.

The drug-likeness and bioavailability scores of all tested compounds are shown in Table 6. According to prediction, the bioavailability score of most of the compounds was about 0.55, except for compounds **2**, **9**, **12**, **14**, and **17** with a value of 0.11. According to the BOILED-Egg illustration (Figure 7A), all compounds are predicted to demonstrate moderate to high gastrointestinal (GI) absorption. Especially, compounds **5**, **6**, **7**, **15**, and **16** are predicted to be passively absorbed by the gastrointestinal tract. In addition, none of the examined compounds are predicted to passively permeate the blood–brain barrier. All compounds showed no violation of Lipinski’s rule of five. Half of the compounds demonstrated a TPSA value < 140 Å, thus indicating their good oral bioavailability. Furthermore, all compounds displayed moderate drug-likeness scores ranging from −0.95 to 0.38, probably due to the enhanced TPSA values and the absence of a basic moiety on rhodanine’s nitrogen. The best in silico prediction was achieved for compound **5**, bearing the basic propyl-morpholine group with a drug-likeness score of 0.24 (Figure 7, Table 6).

### 2.6. ADMET Properties

All compounds have been assessed for their ADMET profile in ADMET Predictor version 10.4 provided by the Simulation Plus software package [1,2,3,4,5,6,7,8,9,10,11,12,13,14,15,16,17,18,19,20,21,22,23,24,25,26,27,28,29,30,31,32,33,34,35,36,37,38,39,40,41,42,43,44,45,46,47,48,49,50,51,52,53]. We have found that compounds **1**, **8**, **9**, **11**, **12**, **14**, and **17** demonstrate optimal properties regarding distribution and, especially, absorption (Table 7). Although these compounds have moderate permeability indices due to their carboxylate moiety, they showed low potential to penetrate the blood–brain barrier (decreased logBB values) as well as acceptable water and salt solubility at a normal blood pH value of 7.4. Moreover, we believe that these compounds could be well absorbed orally after food in the small intestine based on the favorable solubility values in fed state simulated intestinal fluid (FeSSIF). On the other hand, they exhibit low fraction unbound values (less than 6.0%) in comparison with other derivatives in this series (e.g., neutral or basic analogs). Nevertheless, the values of volume of distribution (Vd) and blood-to-plasma ratio (RBP) in humans were predicted to be satisfactory for all compounds in this series.

Furthermore, it has been predicted that the clearance mechanism of all compounds is metabolism (possibility of 74–99%) rather than hepatic uptake or renal elimination (99% both). In view of this fact, a comprehensive in silico analysis of cytochrome P450-mediated metabolism of our compounds was executed (Table 8). First of all, we have found that the different groups incorporated on the nitrogen atom of the rhodanine scaffold can alter compounds’ metabolism. Among the most well-absorbed compounds, **8** and **12** have been assessed to show high clearance through CYP2C9 isoenzyme; thus, they exhibit a CYP risk greater than zero. In addition, compounds **1**, **9**, **11**, **14**, and **17** display zero possibilities of CYP risks. Therefore, they show optimal metabolism characteristics for further studies. We have also estimated that compounds **1**, **2**, **8**–**12**, **14**, and **17** are not CYP2E1 substrates (67–87%), whereas compounds **3**–**7**, **13**, **15**, and **16** are predicted to be good CYP2E1 substrates. Moreover, the majority of compounds may inhibit CYP3A4 isoenzyme (33–80%) except for compounds **1**, **2**, **8**, **11**, and **12** (71–75%). Despite their differences in metabolism characteristics, all compounds share some common characteristics. Particularly, all compounds may inhibit CYP1A2 (68–97%) but not the isoenzymes of CYP2C9 (62–95%), CYP2C19 (94–99%), and CYP2D6 (95%). As illustrated in Table 8, it has been estimated that the major metabolizing enzymes for compounds **1**, **9**, **11**, **14**, and **17** are CYP2C9 and CYP2C19 (over 90% both). On the other hand, all compounds may be also metabolized through CYP2C8 (70–91%) rather than CYP2A6 (67–99%) and CYP2B6 (57–98%). In addition, these compounds may be subjected to glucuronidation by UDP-glucuronosyltransferases 1A3 and 1A9. Last but not least, we suggest that compounds **1**, **9**, **11**, **14**, and **17** may exhibit the most promising metabolism profiles according to the low intrinsic clearance values concerning cytochrome P450 metabolism (CYP-CLint) as well as molecule-level hepatic clearance in humans (HEP-CLint).

**Figure 8:**
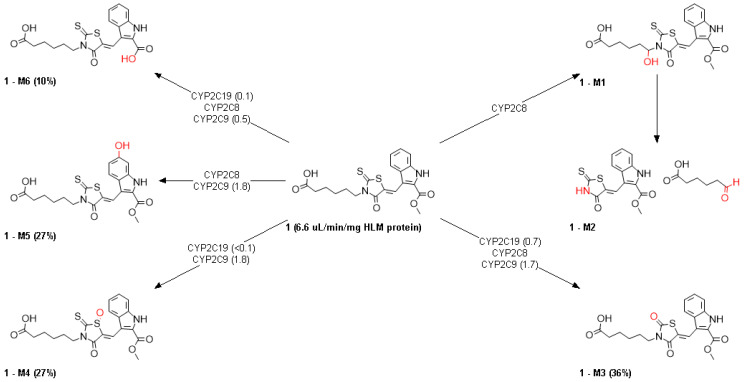
Metabolism of compound **1**.

**Figure 9:**
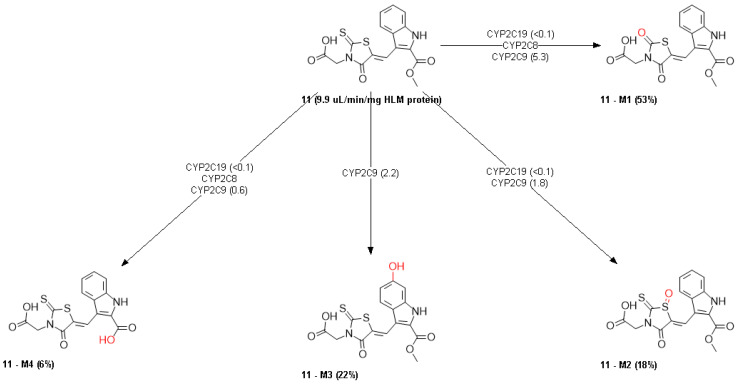
Metabolism of compound **11**.

We have also predicted the metabolic pathways that will take place for the most potent compounds with optimal metabolism profiles, e.g., compounds **1** (Figure 8) and **11** (Figure 9). For both compounds, two main routes of oxidation and demethylation have been found. In particular, demethylated carboxylic acid derivatives account for 10% and 6% of the metabolisms of compounds **1** and **11**, respectively. It is obvious that CYP2C19 and, even more, CYP2C9 are converting the free esters to carboxylic acid derivatives with similar yields. During oxidation, the 6-hydroxy-indole derivatives are also formed in almost equal amounts of 27% and 22% of the metabolic pathway of compounds **1** and **11**, respectively. In addition, it has been shown that CYP2C9 is the major isoenzyme that may be responsible for the sulfone formation of these compounds. Thus, M4 is formed in 27% of the metabolism of compound **1** with regard to M2, which occurs in 18% of compound **11**. Furthermore, it was also assumed that the main metabolites of these compounds could be the 2,4-thiazolidinones, namely 1-M3 and 11-M1, with surprising values of 36% and 53%, respectively. On the other hand, CYP2C8 has also an impact on the metabolism of compound **1** since it may cleave the acidic tail from the nitrogen atom of the rhodanine group, leaving a second metabolite of 6-oxohexanoic acid to be formed. However, this procedure has been established to occur only in compound **1** and not in the case of compound **11**, which possesses the short acetic acid chain. Last but not least, it is worth mentioning that all metabolites of compounds **1** and **11** which have been estimated during at least three metabolic cycles have been found to be non-toxic.

We further studied in detail the possibility of our compounds being substrates or inhibitors against selected human transporters, which play a critical role in pharmacokinetic properties, especially distribution and excretion (Table 9). The ADMET Predictor software revealed that all compounds are both good P-glycoprotein (90–99%) substrates and also breast cancer receptor protein (BCRP) (62–95%) substrates except compound **12**. It has been also assessed that all compounds may not inhibit BCRP (69–97%). Moreover, most of the examined compounds are non-P-glycoprotein inhibitors except compounds **2**–**7**, **10**, **13**, and **16**. In addition, the bile salt export pump (BSEP) which is almost exclusively expressed in the liver, is of great relevance to hepatotoxicity. BSEP inhibition can result in the accumulation of bile salts in the liver, which can lead to cholestasis and drug-induced liver injury (DILI) [54]. In our case, the majority of compounds exhibit no possibilities (≥52%) to inhibit BSEP and thus induce no potential hepatotoxicity.

### 2.7. Cytotoxicity

**Figure 10:**
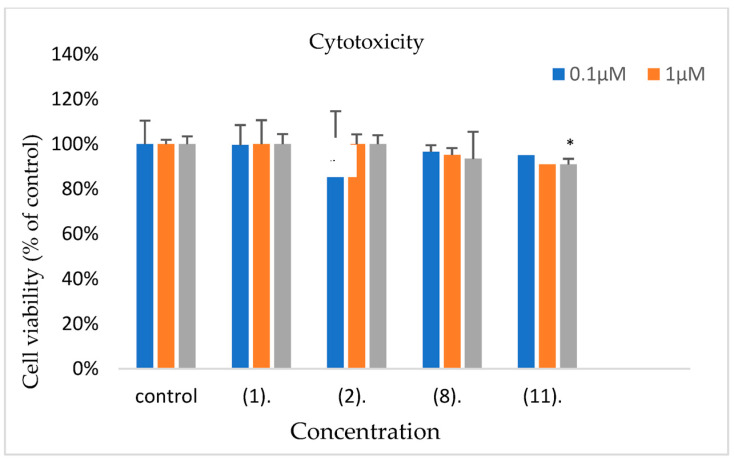
Cell viability levels (%) of MRC-5 cells exposed for 48h at various concentrations in culture to compounds **1**, **2**, **8**, and **11**. All values are presented as mean ± standard deviation (SD) of triplicate independent cellular experiments. With asterisk (*), the significant differences between each concentration and the control group are presented. The significance level was determined at *p* < 0.05.

To evaluate cytotoxicity, compounds **1**, **2**, **8**, and **11** were chosen to be tested in the human normal fetal lung fibroblast MRC-5 cell line within the following range of three concentrations for each substance: 1 × 10^−7^ M (0.1 μM), 1 × 10^−6^ Μ (1 μM), and 1 × 10^−5^ M (10 μΜ). As shown in Figure 10, after 48 h of exposure in culture, all three compounds exhibited no substantial cytotoxicity within the range of concentration tested since viability was ≥91% compared to control untreated culture. By comparing the three agents, only compound **11** exhibited a very slight inhibition in cellular viability at concentrations of 1 μM and 10 μM. More specifically, under this exposure to compound **11**, the cellular viability was measured to the level of 91.0%. The other compounds, **1**, **2**, and **8**, showed no statistically significant change in cellular viability compared to control cells. Overall, these data propose that the tested compounds at the level of concentrations investigated are not cytotoxic in human normal MRC-5 cells.

## 3. Materials and Methods

### 3.1. Prediction of Toxicity

The prediction of toxicity was performed using Protox II webserver [55].

### 3.2. Chemistry

NMR spectra were determined with Varian Mercury VX-400″ (Varian Co., Palo Alto, CA, USA) and AM-300 Bruker 300 MHz. spectrometers in DMSO-d6, and spectra are in Table S1.

Chemical shifts of nuclei ^1^H were measured relatively in the residual signals of deuteron solvent (δ = 2.50 ppm). Coupling constants (*J*) are reported in Hz. Τhe assignment was based on 2D NMR techniques. Melting points were determined using the Fisher-Johns Melting Point Apparatus (Fisher Scientific, Hampton, NH, USA) and are uncorrected. Elemental analysis was performed by the classical method of microanalysis.

All compounds were synthesized analogous to the process described in our previous paper [6].

A mixture of a corresponding amino acid (50 mmol), a cooled solution of KOH in water (20 mL) (150 mmol in case of dicarbonic acids), and CS_2_ (mmol) were stirred in a flat-bottomed flask until a solution was formed. A solution of monochloracetic acid (55 mmol) was added with stirring, pre-neutralized with sodium bicarbonate (55 mmol) in water (25 mL), and left at room temperature for 2 days.

Then, to the formed solution, a 6N HCl solution (20 mL) was added and heated to boiling and kept at a slow boil for 1 h. After cooling, the precipitate formed was filtered off, dried, and recrystallized, alternately, from diluted acetic acid, ethanol, and toluene.

In a round-bottom flask equipped with a reflux condenser, 2.5 mmol of methyl 3-formyl-5,6-disabstituted-1*H*-indole-2-carboxylate, 3.3 mmol of 3-substituted-2-thioxothiazolidin-4-one, 2.5 mmol of ammonium acetate, and 5 mL of acetic acid were placed. The reaction mixture was boiled for 2 h and cooled. Then, the precipitate was filtered off, washed with acetic acid and water, dried, and recrystallized.

(*Z*)-6-(5-((2-(methoxycarbonyl)-1*H*-indol-3-yl)methylene)-4-oxo-2-thioxothiazolidin-3-yl)hexanoic acid (**1**). m.p. 135–138 °C. ^1^H NMR (300 MHz, DMSO-*d6*) δ 12.06 (s, 2H,NH, OH), 8.49 (s, 1H), 7.79 (s, 1H), 7.58 (s, 1H), 7.35 (s, 1H), 7.24 (s, 1H), 4.09 (s, 2H), 4.00 (s, 3H, CH_3_), 2.21 (s, 2H), 1.75 (s, 2H), 1.65 (s, 2H), 1.45 (s, 2H). Anal. Calcd. For C_20_H_20_N_2_O_5_S_2_ (%): C, 55.54; H, 4.66; N, 6.48; O, 18.50. Found (%): C, 55.50; H, 4.69; N, 6.52; O, 18.47.
(*Z*)-4-(5-((2-(methoxycarbonyl)-1*H*-indol-3-yl)methylene)-4-oxo-2-thioxothiazolidin-3-yl)benzoic acid (**2**). m.p. 294–295 °C. ^1^H NMR (300 MHz, DMSO-*d6*) δ 12.69 (s, 1H, OH), 8.51 (s, 1H), 8.16 (d, *J* = 8.1 Hz, 2H), 7.87 (d, *J* = 8.2 Hz, 1H), 7.60 (d, *J* = 8.1 Hz, 1H), 7.47 (d, *J* = 8.2 Hz, 2H), 7.37 (t, *J* = 7.6 Hz, 1H), 7.28 (t, *J* = 7.5 Hz, 1H), 4.00 (s, 3H, CH_3_). Anal. Calcd. For C_21_H_14_N_2_O_5_S_2_ (%): C, 57.52; H, 3.22; N, 6.39; O, 18.24. Found (%): C, 57.54; H, 3.20; N, 6.42; O, 18.25.
(*Z*)-methyl 3-((3-(3-hydroxyphenyl)-4-oxo-2-thioxothiazolidin-5-ylidene)methyl)-1*H*-indole-2-carboxylate (**3**). m.p. 245–247 °C. ^1^H NMR (300 MHz, DMSO-*d6*) δ 12.66 (s, 1H, OH), 9.61 (s, 1H, NH), 8.48 (s, 1H), 7.87 (d, *J* = 8.2 Hz, 1H), 7.59 (d, *J* = 8.3 Hz, 1H), 7.31 (dq, *J* = 7.2, 22.3 Hz, 3H), 6.90 (d, *J* = 8.4 Hz, 1H), 6.71 (d, *J* = 5.2 Hz, 2H), 4.00 (s, 3H,CH_3_). Anal. Calcd. For C_20_H_14_N_2_O_4_S_2_ (%): C, 58.52; H, 3.44; N, 6.82; O, 15.59. Found (%): C, 58.50; H, 3.43; N, 6.87; O, 15.53.
(*Z*)-methyl 3-((3-(4-hydroxyphenyl)-4-oxo-2-thioxothiazolidin-5-ylidene)methyl)-1*H*-indole-2-carboxylate (**4**). m.p. 282–284 °C. ^1^H NMR (300 MHz, DMSO-*d6*) δ 12.64 (s, 1H, OH), 9.62 (s, 1H, NH), 8.47 (s, 1H), 7.86 (d, *J* = 8.2 Hz, 1H), 7.59 (d, *J* = 8.3 Hz, 1H), 7.36 (t, *J* = 7.7 Hz, 1H), 7.26 (t, *J* = 7.6 Hz, 1H), 7.08 (d, *J* = 8.3 Hz, 2H), 6.90 (d, *J* = 8.3 Hz, 2H), 4.00 (s, 3H, CH_3_). Anal. Calcd. For C_20_H_14_N_2_O_4_S_2_ (%): C, 58.52; H, 3.44; N, 6.82; O, 15.59. Found (%): C, 58.50; H, 3.47; N, 6.86; O, 15.55.
(*Z*)-methyl 3-((3-(3-morpholinopropyl)-4-oxo-2-thioxothiazolidin-5-ylidene)methyl)-1*H*-indole-2-carboxylate (**5**). m.p. 213–214 °C. ^1^H NMR (300 MHz, DMSO-*d6*) δ 12.64 (s, 1H, NH), 8.47 (s, 1H), 7.78 (d, *J* = 8.2 Hz, 1H), 7.58 (d, *J* = 8.3 Hz, 1H), 7.35 (t, *J* = 7.6 Hz, 1H), 7.24 (t, *J* = 7.6 Hz, 1H), 4.18 (t, *J* = 7.0 Hz, 2H), 4.00 (s, 2H), 3.55 (t, *J* = 4.6 Hz, 4H, CH_3_), 2.58–2.29 (m, 8H), 1.89 (p, *J* = 6.8 Hz, 2H). Anal. Calcd. For C_21_H_23_N_3_O_4_S_2_ (%): C, 56.61; H, 5.20; N, 9.43; O, 14.36. Found (%): C, 56.64; H, 5.18; N, 9.45; O, 14.30.
(*Z*)-methyl 3-((3-morpholino-4-oxo-2-thioxothiazolidin-5-ylidene)methyl)-1*H*-indole-2-carboxylate (**6**). m.p. 278–280 °C. ^1^H NMR (300 MHz) δ 12.65 (s, 1H, NH), 8.42 (s, 1H), 7.78 (d, *J* = 8.1 Hz, 1H), 7.57 (d, *J* = 8.3 Hz, 1H), 7.34 (t, *J* = 7.7 Hz, 1H), 7.23 (t, *J* = 7.6 Hz, 1H), 4.00 (s, 3H, CH_3_), 3.05 (d, *J* = 22.9 Hz, 4H), 2.50 (s, 3H). Anal. Calcd. For C_18_H_17_N_3_O_4_S_2_ (%): C, 53.58; H, 4.25; N, 10.41; O, 15.86. Found (%): C, 53.60; H, 4.21; N, 10.46; O, 15.83.
(*Z*)-methyl 3-((3-(furan-2-ylmethyl)-4-oxo-2-thioxothiazolidin-5-ylidene)methyl)-1*H*-indole-2-carboxylate (**7**). ^1^H NMR (300 MHz, DMSO-*d6*) δ 12.65 (s, 1H, NH), 8.52 (s, 1H), 7.79 (d, *J* = 8.2 Hz, 1H), 7.57 (d, *J* = 8.2 Hz, 1H), 7.47 (s, 1H), 7.34 (t, *J* = 7.7 Hz, 1H), 7.23 (t, *J* = 7.6 Hz, 1H), 6.41 (s, 1H), 6.36 (s, 1H), 5.26 (s, 2H), 4.00 (s, 3H, CH_3_). m.p. 212–213 °C. Anal. Calcd. For C_19_H_14_N_2_O_4_S_2_ (%): C, 57.27; H, 3.54; N, 7.03; O, 16.06. Found (%): C, 57.31; H, 3.50; N, 7.08; O, 16.02.
(*Z*)-2-(5-((5-fluoro-2-(methoxycarbonyl)-1*H*-indol-3-yl)methylene)-4-oxo-2-thioxothiazolidin-3-yl)-3-methylbutanoic acid (**8**). ^1^H NMR (300 MHz, DMSO-*d6*) δ 8.44 (s, 1H, NH), 7.58 (dd, *J* = 4.7, 9.0 Hz, 1H), 7.48 (d, *J* = 9.7 Hz, 1H), 7.15 (t, *J* = 9.2 Hz, 1H), 5.12 (d, *J* = 9.6 Hz, 1H), 4.00 (s, 3H, O-CH_3_), 2.80 (s, 1H, CH-(CH_3_)_2_), 1.28 (d, *J* = 6.2 Hz, 3H, CH-CH_3_), 0.84 (d, *J* = 6.7 Hz, 3H, CH-CH_3_). m.p. 234–235 °C. Anal. Calcd. For C_19_H_17_FN_2_O_5_S_2_ (%): C, 52.28; H, 3.93; F, 4.35; N, 6.42; O, 18.33. Found (%): C, 52.23; H, 3.90; F, 4.28; N, 6.54; O, 18.29.
(*Z*)-4-(5-((5-fluoro-2-(methoxycarbonyl)-1*H*-indol-3-yl)methylene)-4-oxo-2-thioxothiazolidin-3-yl)butanoic acid (**9**). m.p. 192–193 °C. ^1^H NMR (300 MHz, DMSO-*d6*) δ 12.72 (s, 1H, NH), 11.90 (s, 1H, OH), 8.38 (s, 1H), 7.57 (dd, *J* = 4.7, 9.0 Hz, 1H), 7.46 (dd, *J* = 2.3, 9.9 Hz, 1H), 7.14 (td, *J* = 2.5, 9.1 Hz, 1H), 4.14 (t, *J* = 7.0 Hz, 2H), 3.99 (s, 3H, CH_3_), 2.32 (t, *J* = 7.4 Hz, 2H), 1.98 (p, *J* = 7.4 Hz, 2H). Anal. Calcd. For C_18_H_15_FN_2_O_5_S_2_ (%): C, 51.18; H, 3.58; F, 4.50; N, 6.63; O, 18.94. Found (%): C, 51.23; H, 3.55; F, 4.48; N, 6.69; O, 18.90.
(*Z*)-methyl 5-fluoro-3-((3-(4-hydroxyphenyl)-4-oxo-2-thioxothiazolidin-5-ylidene)methyl)-1*H*-indole-2-carboxylate (**10**). m.p. 267–268 °C. ^1^*H* NMR (300 MHz, DMSO-*d6*) δ 12.74 (s, 1H, OH), 9.61 (s, 1H, NH), 8.37 (s, 1H), 7.64 – 7.47 (m, 2H), 7.15 (t, *J* = 8.7 Hz, 1H), 7.07 (d, *J* = 8.4 Hz, 2H), 6.90 (d, *J* = 8.4 Hz, 2H), 3.99 (s, 3H, O-CH_3_). Anal. Calcd. For C_20_H_13_FN_2_O_4_S_2_ (%): C, 56.06; H, 3.06; F, 4.43; N, 6.54; O, 14.94. Found (%): C, 56.12; H, 3.02; F, 4.39; N, 6.58; O, 14.87.
(*Z*)-2-(5-((2-(methoxycarbonyl)-1*H*-indol-3-yl)methylene)-4-oxo-2-thioxothiazolidin-3-yl)acetic acid (**11**). m.p. 292–294 °C. ^1^H NMR (300 MHz, DMSO-*d6*) δ 8.51 (s, 1H), 7.80 (d, *J* = 8.3 Hz, 1H), 7.57 (d, *J* = 8.3 Hz, 1H), 7.34 (t, *J* = 7.6 Hz, 1H), 7.24 (t, *J* = 7.6 Hz, 1H), 4.65 (s, 2H), 3.99 (s, 3H,O-CH_3_). Anal. Calcd. For C_16_H_12_N_2_O_5_S_2_ (%): C, 51.05; H, 3.21; N, 7.44; O, 21.25. Found (%): C, 51.11; H, 3.27; N, 7.33; O, 21.20.
(*Z*)-2-(5-((2-(methoxycarbonyl)-1*H*-indol-3-yl)methylene)-4-oxo-2-thioxothiazolidin-3-yl)-3-methylbutanoic acid (**12**). m.p. 256–258 °C. ^1^H NMR (300 MHz, DMSO-*d6*) δ 8.51 (s, 1H), 7.82 (d, *J* = 8.2 Hz, 1H), 7.58 (d, *J* = 8.2 Hz, 1H), 7.34 (t, *J* = 7.7 Hz, 1H), 7.24 (t, *J* = 7.6 Hz, 1H), 5.13 (d, *J* = 9.2 Hz, 1H), 4.00 (s, 3H), 2.79 (s, 1H, CH-(CH_3_)_2_), 1.28 (d, *J* = 6.3 Hz, 3H, CH-CH_3_), 0.84 (d, *J* = 6.8 Hz, 3H, CH-CH_3_). Anal. Calcd. For C_19_H_18_N_2_O_5_S_2_ (%): C, 54.53; H, 4.34; N, 6.69; O, 19.12. Found (%): C, 54.49; H, 4.37; N, 6.71; O, 19.10.
(*Z*)-methyl 3-((3-(3-fluorophenyl)-4-oxo-2-thioxothiazolidin-5-ylidene)methyl)-6-methoxy-1*H*-indole-2-carboxylate (**13**). m.p. 238–240 °C. ^1^H NMR (300 MHz, DMSO-*d6*) δ 12.65 (s, 1H, NH), 8.52 (s, 1H), 7.79 (d, *J* = 8.2 Hz, 1H), 7.57 (d, *J* = 8.2 Hz, 1H), 7.47 (s, 1H), 7.34 (t, *J* = 7.7 Hz, 1H), 7.23 (t, *J* = 7.6 Hz, 1H), 6.41 (s, 1H), 6.36 (s, 1H), 5.26(s, 3H, O-CH_3_), 4.00 (s, 3H, O-CH_3_). Anal. Calcd. For C_21_H_15_FN_2_O_4_S_2_ (%): C, 57.00; H, 3.42; F, 4.29; N, 6.33; O, 14.46. Found (%): C, 57.12; H, 3.39; F, 4.32; N, 6.38; O, 14.51.
(*Z*)-3-(5-((5-fluoro-2-(methoxycarbonyl)-1*H*-indol-3-yl)methylene)-4-oxo-2-thioxothiazolidin-3-yl)propanoic acid (**14**). m.p. 275–276 °C. ^1^H NMR (300 MHz, DMSO-*d6*) δ 12.76 (s, 1H, OH), 12.30 (s, 1H, NH), 8.39 (s, 1H), 7.57 (dd, *J* = 4.6, 9.0 Hz, 1H), 7.44 (d, *J* = 9.8 Hz, 1H), 7.15 (t, *J* = 8.9 Hz, 1H), 4.29 (t, *J* = 7.9 Hz, 2H), 3.99 (s, 3H, CH_3_), 2.64 (t, *J* = 8.1 Hz, 2H). Anal. Calcd. For C_17_H_13_FN_2_O_5_S_2_ (%): C, 49.99; H, 3.21; F, 4.65; N, 6.86; O, 19.59. Found (%): C, 49.92; H, 3.25; F, 4.60; N, 6.90; O, 19.62.
(*Z*)-methyl 5-fluoro-3-((3-methyl-4-oxo-2-thioxothiazolidin-5-ylidene)methyl)-1*H*-indole-2-carboxylate (**15).** m.p. 234–244 °C. ^1^H NMR (300 MHz) δ 12.75 (s, 1H, NH), 8.40 (s, 1H), 7.57 (dd, *J* = 4.7, 9.0 Hz, 1H), 7.43 (d, *J* = 9.7 Hz, 1H), 7.15 (t, *J* = 9.0 Hz, 1H), 3.99 (s, 3H, O-CH_3_), 3.48 (s, 3H, N-CH_3_). Anal. Calcd. For C_15_H_11_FN_2_O_3_S_2_ (%): C, 51.42; H, 3.16; F, 5.42; N, 7.99; O, 13.70. Found (%): C, 51.40; H, 3.21; F, 5.45; N, 7.93; O, 13.75.
(*Z*)-methyl 5-fluoro-3-((3-morpholino-4-oxo-2-thioxothiazolidin-5-ylidene)methyl)-1*H*-indole-2-carboxylate (**16**). m.p. 273–274 °C. ^1^H NMR (300 MHz, DMSO-*d6*) δ 12.76 (s, 1H, NH), 8.32 (s, 1H), 7.57 (dd, *J* = 4.6, 9.1 Hz, 1H), 7.46 (d, *J* = 9.6 Hz, 1H), 7.15 (t, *J* = 9.1 Hz, 1H), 4.00 (s, 3H, CH_3_), 3.77 (dd, *J* = 16.8, 29.8 Hz, 6H), 3.05 (d, *J* = 26.1 Hz, 3H). Anal. Calcd. For C_18_H_16_FN_3_O_4_S_2_ (%): C, 51.30; H, 3.83; F, 4.51; N, 9.97; O, 15.18. Found (%): C, 51.36; H, 3.79; F, 4.54; N, 9.93; O, 15.21.
(*Z*)-3-(5-((2-(methoxycarbonyl)-1*H*-indol-3-yl)methylene)-4-oxo-2-thioxothiazolidin-3-yl)propanoic acid (**17**). m.p. 265–266 °C. ^1^H NMR (300 MHz, DMSO-*d6*) δ 12.65 (s, 1H, NH), 12.30 (s, 1H, OH), 8.49 (s, 1H), 7.78 (d, *J* = 8.2 Hz, 1H), 7.57 (d, *J* = 8.3 Hz, 1H), 7.35 (t, *J* = 7.7 Hz, 1H), 7.24 (t, *J* = 7.4 Hz, 1H), 4.29 (t, *J* = 7.9 Hz, 2H), 4.00 (s, 3H, O-CH_3_), 2.64 (t, *J* = 8.1 Hz, 2H). Anal. Calcd. For C_17_H_14_N_2_O_5_S_2_ (%): C, 52.30; H, 3.61; N, 7.17; O, 20.49. Found (%): C, 52.28; H, 3.65; N, 7.21; O, 20.52.

### 3.3. Biological Evaluation

#### 3.3.1. Antibacterial Activity

The following Gram-negative bacteria: *Escherichia coli* (ATCC 35210), *Enterobacter cloacae* (clinical isolate), *Salmonella typhimurium* (ATCC 13311), as well as Gram-positive bacteria: *Listeria monocytogenes* (NCTC 7973), *Bacillus cereus* (clinical isolate), and *Staphylococcus aureus* (ATCC 6538) were used. The organisms were obtained from the Mycological Laboratory, Department of Plant Physiology, Institute for Biological Research “Siniša Stankovic”, Belgrade, Serbia. The minimum inhibitory (MIC) and minimum bactericidal (MBC) concentrations were determined by the modified microdilution method, as previously reported [6,41].

#### 3.3.2. Antifungal Activity

For the antifungal bioassays, six fungi were used: *Aspergillus niger* (ATCC 6275), *Aspergillus fumigatus* (ATCC 1022), *Aspergillus versicolor* (ATCC 11730), *Penicillium funiculosum* (ATCC 36839), *Trichoderma viride* (IAM 5061), and *Penicillium verrucosum var. cyclopium* (food isolate). The organisms were obtained from the Mycological Laboratory, Department of Plant Physiology, Institute for Biological Research “Siniša Stankovic”, Belgrade, Serbia. All experiments were performed in duplicate [56,57].

### 3.4. Docking Studies

AutoDock 4.2^®^ software was used for the in silico studies, and a detailed procedure is reported in our previous paper [58].

### 3.5. Drug Likeness

Five filters were used to predict drug-likeness [59] by the Molsoft software and SwissADME program (http://swissadme.ch, accessed on 25 October 2022) via the ChemAxon’s Marvin JS structure drawing tool.

### 3.6. ADMET

The prediction of the pharmacokinetic and toxicity (phospholipidosis, hERG-cardiotoxicity) profile of compounds was executed by ADMET Predictor 10.4 [51,52,53].

### 3.7. Assessment of Cytotoxicity

The normal human fetal lung fibroblast MRC-5 cell line was maintained and used in our laboratory (Dr. I.s.Vizirianakis, Laboratory of Pharmacology, School of Pharmacy, Aristotle University of Thessaloniki, Greece) (passage 15 < 40), as previously published [60]. Cells were grown in culture under the following conditions: 37 °C, humidified atmosphere containing 5% *v*/*v* CO_2_, in DMEM (Dulbecco’s modified Eagle Medium) enriched with 10% FBS and 1% PS. The compounds tested were dissolved in DMSO and stored at 4 °C. For the assessment of cytotoxicity, the cells were cultivated in a 96-well plate at an initial concentration of 5 × 10^4^ cells/mL and allowed to attach for 20 h before the addition of the compounds at concentrations of 1 × 10^−5^ M (10 μM), 1 × 10^−6^ M(1 μM), and 1 × 10^−7^ M (0.1 μM). It is worth noting that the concentration of DMSO in the culture was 0.02% *v*/*v*, in which no detectable effect on cell toxicity was observed. To assess the cytotoxicity of each compound, the cells were allowed to grow for 48 h under the effect of each substance. Subsequently, Cell Counting Kit-8 (CCK8, St. Louis, MO, USA, Sigma-Aldrich) reagent was added to each well and incubated for 4 h at 37 °C. After the incubation, the OD for each well was determined at 450 nm in a multifunction microplate reader. Wells containing only the CCK-8 reagent were used as blank control. Data are presented as mean ± standard deviation (SD) of triplicate incubations. Statistical *t*-test and one-way analysis of variance (ANOVA) were performed via the use of the SPSS program and the significance level was determined at *p* < 0.05.

## 4. Conclusions

Seventeen (*Z*)-methyl-3-(4-oxo-2-thioxothiazolidin-5-ylidene)methyl)-1*H*-indole-2-carboxylates **1**–**17** were designed, synthesized, and evaluated in silico and experimentally for their antimicrobial actions against a panel of Gram-positive and Gram-negative bacteria and fungi.

The evaluation revealed that all compounds were more potent than both reference drugs, ampicillin, and streptomycin against all bacteria tested. *En. cloacae* appeared to be the most sensitive bacterial strain towards our derivatives, whereas *E. coli* was the most resistant one, followed by *M. flavus*.

Concerning antifungal action, the tested compounds exhibited very good to excellent activity against all the fungal species tested, being more active than ketoconazole and bifonazole. Most of the compounds appeared to be very potent against *A. ochraceus* and *T. viride* with the last one being the most sensitive to tested compounds. Filamentous *A. fumigatus* was the most resistant strain.

It can be observed that the growth of both Gram-negative and Gram-positive bacteria and fungi responded differently to the tested compounds, suggesting that different substituents may lead to different modes of action or that the metabolism of some bacteria/fungi was able to overcome the effect of the compounds or adapt to it.

Docking analysis to DNA Gyrase, Thymidylate kinase, and *E. coli* MurB indicated MurB inhibition as a putative antibacterial mechanism of compounds tested, while docking analysis to 14α-lanosterol demethylase (CYP51) and tetrahydrofolate reductase of *Candida albicans* pointed out a probable implication of CYP51 reductase in the antifungal activity of the compounds.

Even though compounds displayed moderate to good drug-likeness scores (−0.89 to +0.24), no violation of the Lipinski rule was observed. Furthermore, according to predicted results, six out of seventeen compounds can be orally absorbed since their TPSA are less than 140 Å. All compounds have been assessed for their ADMET profile in ADMET Predictor version 10.4, provided by the Simulation Plus software package. We have found that compounds **1**, **8**, **9**, **11**, **12**, **14**, and **17** demonstrate optimal properties regarding distribution and especially absorption.

The prediction of metabolic pathways that could take place for the potent compounds **1** and **11** with optimal metabolism profiles indicated the transformation of rhodanine to thiazolidinone and indole ring hydroxylation as the two main routes. The evaluation of the cytotoxicity of compounds on normal human fetal lung fibroblast MRC-5 cell lines revealed that compounds are not toxic.

Thus, these derivatives can be considered as lead compounds for the development of novel potent and safe antimicrobial agents.

## Figures and Tables

**Table 1:** The structures of evaluated compounds.

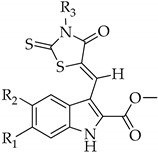											
N	R_1_	R_2_	R_3_	N	R_1_	R_2_	R_3_	N	R_1_	R_2_	R_3_
**1**	H	H	-(CH_2_)_5_COOH	**7**	H	H	2-methylfuran	**13**	-OCH_3_	H	3-FC_6_H_4_
**2**	H	H	4-COOHC_6_H_4_	**8**	H	F	-C(CH_3_)_2_COOH	**14**	H	F	-(CH_2_)_2_COOH
**3**	H	H	3-OHC_6_H_4_	**9**	H	F	-(CH_2_)_3_COOH	**15**	H	F	-CH_3_
**4**	H	H	4-OHC_6_H_4_	**10**	H	F	4-OHC_6_H_4_	**16**	H	F	morpholine
**5**	H	H	4-propylmorpholine	**11**	H	H	-CH_2_COOH	**17**	H	H	-(CH_2_)_2_COOH
**6**	H	H	morpholine	**12**	H	H	-C(CH_3_)_2_COOH				

**Table 2:** Antibacterial activity of target compounds (MIC and MBC in mg/mL).

Compounds		*S.a.*	*B.c.*	*M.f.*	*L.m.*	*P.a.*	*E. coli*	*En.cl*	*S.t.*
**1**	MIC	0.022 ± 0.005	0.015 ± 0.009	0.022 ± 0.005	0.022 ± 0.004	0.022 ± 0.005	0.015 ± 0.009	0.022 ± 0.005	0.015 ± 0.009
	MBC	0.03 ± 0.01	0.03 ± 0.01	0.03 ± 0.01	0.06 ± 0.006	0.06 ± 0.006	0.03 ± 0.01	0.03 ± 0.01	0.03 ± 0.01
**2**	MIC	0.015 ± 0.009	0.015 ± 0.009	0.022 ± 0.005	0.03 ± 0.01	0.03 ± 0.01	0.022 ± 0.005	0.015 ± 0.009	0.022 ± 0.005
	MBC	0.03 ± 0.01	0.03 ± 0.01	0.03 ± 0.01	0.06 ± 0.006	0.06 ± 0.006	0.03 ± 0.01	0.03 ± 0.01	0.03 ± 0.01
**3**	MIC	0.015 ± 0.009	0.015 ± 0.009	0.045 ± 0.003	0.03 ± 0.01	0.03 ± 0.01	0.022 ± 0.005	0.015 ± 0.009	0.015 ± 0.009
	MBC	0.03 ± 0.01	0.03 ± 0.01	0.06 ± 0.006	0.06 ± 0.006	0.06 ± 0.006	0.03 ± 0.01	0.03 ± 0.01	0.03 ± 0.01
**4**	MIC	0.03 ± 0.01	0.03 ± 0.01	0.045 ± 0.003	0.045 ± 0.001	0.03 ± 0.01	0.045 ± 0.003	0.015 ± 0.009	0.045 ± 0.003
	MBC	0.06 ± 0.006	0.06 ± 0.006	0.06 ± 0.006	0.06 ± 0.006	0.06 ± 0.006	0.06 ± 0.006	0.03 ± 0.01	0.06 ± 0.006
**5**	MIC	0.015 ± 0.009	0.03 ± 0.01	0.045 ± 0.003	0.03 ± 0.01	0.03 ± 0.01	0.03 ± 0.01	0.015 ± 0.009	0.022 ± 0.005
	MBC	0.03 ± 0.01	0.06 ± 0.006	0.06 ± 0.006	0.06 ± 0.006	0.06 ± 0.006	0.06 ± 0.006	0.03 ± 0.01	0.03 ± 0.01
**6**	MIC	0.015 ± 0.009	0.03 ± 0.01	0.045 ± 0.003	0.03 ± 0.01	0.03 ± 0.01	0.06 ± 0.006	0.015 ± 0.009	0.03 ± 0.01
	MBC	0.03 ± 0.01	0.06 ± 0.006	0.06 ± 0.006	0.06 ± 0.006	0.06 ± 0.006	0.12 ± 0.01	0.03 ± 0.01	0.06 ± 0.006
**7**	MIC	0.015 ± 0.009	0.03 ± 0.01	0.045 ± 0.003	0.03 ± 0.01	0.03 ± 0.01	0.03 ± 0.01	0.015 ± 0.009	0.015 ± 0.009
	MBC	0.03 ± 0.01	0.06 ± 0.006	0.06 ± 0.006	0.06 ± 0.006	0.06 ± 0.006	0.06 ± 0.006	0.03 ± 0.01	0.03 ± 0.01
**8**	MIC	0.03 ± 0.01	0.03 ± 0.01	0.03 ± 0.01	0.015 ± 0.001	0.004 ± 0.005	0.008 ± 0.001	0.004 ± 0.005	0.008 ± 0.009
	MBC	0.06 ± 0.006	0.06 ± 0.006	0.06 ± 0.006	0.03 ± 0.01	0.008 ± 0.0006	0.015 ± 0.009	0.008 ± 0.0006	0.015 ± 0.009
**9**	MIC	0.03 ± 0.01	0.03 ± 0.01	0.03 ± 0.01	0.03 ± 0.01	0.03 ± 0.01	0.03 ± 0.01	0.03 ± 0.01	0.03 ± 0.01
	MBC	0.06 ± 0.006	0.06 ± 0.006	0.06 ± 0.006	0.06 ± 0.006	0.06 ± 0.006	0.06 ± 0.006	0.06 ± 0.006	0.06 ± 0.006
**10**	MIC	0.03 ± 0.01	0.03 ± 0.01	0.045 ± 0.003	0.03 ± 0.01	0.03 ± 0.01	0.03 ± 0.01	0.03 ± 0.01	0.045 ± 0.003
	MBC	0.06 ± 0.006	0.06 ± 0.006	0.06 ± 0.006	0.06 ± 0.006	0.06 ± 0.006	0.06 ± 0.006	0.06 ± 0.006	0.06 ± 0.006
**11**	MIC	0.008 ± 0.0006	0.008 ± 0.0006	0.03 ± 0.01	0.03 ± 0.01	0.011 ± 0.01	0.015 ± 0.009	0.011 ± 0.01	0.015 ± 0.009
	MBC	0.015 ± 0.009	0.015 ± 0.009	0.06 ± 0.006	0.06 ± 0.006	0.03 ± 0.01	0.03 ± 0.01	0.03 ± 0.01	0.03 ± 0.01
**12**	MIC	0.03 ± 0.01	0.03 ± 0.01	0.03 ± 0.01	0.015 ± 0.001	0.015 ± 0.009	0.015 ± 0.009	0.03 ± 0.01	0.004 ± 0.005
	MBC	0.06 ± 0.006	0.06 ± 0.006	0.06 ± 0.006	0.03 ± 0.01	0.03 ± 0.01	0.03 ± 0.01	0.06 ± 0.006	0.008 ± 0.009
**13**	MIC	0.015 ± 0.009	0.03 ± 0.01	0.045 ± 0.003	0.03 ± 0.01	0.03 ± 0.01	0.045 ± 0.003	0.015 ± 0.009	0.03 ± 0.01
	MBC	0.03 ± 0.01	0.06 ± 0.006	0.06 ± 0.006	0.06 ± 0.006	0.06 ± 0.006	0.06 ± 0.006	0.03 ± 0.01	0.06 ± 0.006
**14**	MIC	0.03 ± 0.01	0.03 ± 0.01	0.045 ± 0.003	0.045 ± 0.003	0.015 ± 0.009	0.045 ± 0.003	0.015 ± 0.009	0.045 ± 0.003
	MBC	0.06 ± 0.006	0.06 ± 0.006	0.06 ± 0.006	0.06 ± 0.006	0.03 ± 0.01	0.06 ± 0.006	0.03 ± 0.01	0.06 ± 0.006
**15**	MIC	0.022 ± 0.005	0.03 ± 0.01	0.045 ± 0.003	0.045 ± 0.003	0.03 ± 0.01	0.045 ± 0.003	0.03 ± 0.01	0.045 ± 0.003
	MBC	0.03 ± 0.01	0.06 ± 0.006	0.06 ± 0.006	0.06 ± 0.006	0.06 ± 0.006	0.06 ± 0.006	0.06 ± 0.006	0.06 ± 0.006
**16**	MIC	0.022 ± 0.005	0.015 ± 0.009	0.045 ± 0.003	0.045 ± 0.003	0.03 ± 0.01	0.03 ± 0.01	0.045 ± 0.003	0.045 ± 0.003
	MBC	0.03 ± 0.01	0.03 ± 0.01	0.06 ± 0.006	0.06 ± 0.006	0.06 ± 0.006	0.06 ± 0.006	0.06 ± 0.006	0.06 ± 0.006
**17**	MIC	0.008 ± 0.0006	0.03 ± 0.01	0.03 ± 0.01	0.03 ± 0.01	0.008 ± 0.000	0.045 ± 0.003	0.008 ± 0.0006	0.03 ± 0.01
	MBC	0.015 ± 0.00	0.06 ± 0.006	0.06 ± 0.006	0.06 ± 0.006	0.015 ± 0.009	0.06 ± 0.006	0.015 ± 0.009	0.06 ± 0.006
Ampicillin	MIC	0.10 ± 0.05	0.006 ± 0.003	0.25 ± 0.09	0.15 ± 0.03	0.30 ± 0.03	0.10 ± 0.05	0.25 ± 0.09	0.15 ± 0.03
	MBC	0.15 ± 0.05	0.025 ± 0.00	0.50 ± 0.1	0.30 ± 0.03	0.50 ± 0.1	0.20 ± 0.00	0.50 ± 0.1	0.20 ± 0.009
Streptomycin	MIC	0.10 ± 0.00	0.0015 ± 0.0002	0.20 ± 0.00	0.15 ± 0.03	0.10 ± 0.05	0.10 ± 0.05	0.20 ± 0.005	0.10 ± 0.05
	MBC	0.20 ± 0.00	0.003 ± 0.0005	0.30 ± 0.03	0.30 ± 0.03	0.20 ± 0.00	0.20 ± 0.00	0.30 ± 0.03	0.20 ± 0.009

*B.c.*—*B. cereus*, *M.f.*—*M. flavus*, *S.a.*—*S. aurues*, *L.m.*—*L. monocytogenes*, *En.c.*—*En. cloacae*, *P.a.*—*P. aeruginosa*, *S.t.*—*S. typhimurium*.

**Table 3:** Antifungal activity of target compounds (MIC and MFC in mg/mL).

Compounds		*A.f.*	*A.v.*	*A.o.*	*A.n.*	*T.v.*	*P.o.*	*P.f.*	*P.v.c.*
**1**	MIC	0.06 ± 0.006	0.03 ± 0.01	0.008 ± 0.0006	0.022 ± 0.005	0.015 ± 0.003	0.004 ± 0.001	0.015 ± 0.003	0.03 ± 0.01
	MFC	0.12 ± 0.04	0.06 ± 0.006	0.015 ± 0.003	0.03 ± 0.01	0.03 ± 0.01	0.008 ± 0.0006	0.03 ± 0.01	0.06 ± 0.006
**2**	MIC	0.03 ± 0.01	0.015 ± 0.003	0.11 ± 0.01	0.022 ± 0.005	0.008 ± 0.0006	0.015 ± 0.003	0.015 ± 0.003	0.03 ± 0.01
	MFC	0.06 ± 0.006	0.03 ± 0.01	0.015 ± 0.003	0.03 ± 0.01	0.015 ± 0.003	0.03 ± 0.01	0.03 ± 0.01	0.06 ± 0.006
**3**	MIC	0.03 ± 0.01	0.008 ± 0.0006	0.008 ± 0.0006	0.11 ± 0.01	0.004 ± 0.001	0.008 ± 0.0006	0.008 ± 0.0006	0.015 ± 0.003
	MFC	0.06 ± 0.006	0.015 ± 0.003	0.015 ± 0.003	0.015 ± 0.003	0.008 ± 0.0006	0.015 ± 0.003	0.015 ± 0.003	0.03 ± 0.01
**4**	MIC	0.06 ± 0.006	0.008 ± 0.0006	0.008 ± 0.0006	0.015 ± 0.003	0.008 ± 0.0006	0.015 ± 0.003	0.015 ± 0.003	0.03 ± 0.01
	MFC	0.12 ± 0.04	0.015 ± 0.003	0.015 ± 0.003	0.03 ± 0.01	0.015 ± 0.003	0.03 ± 0.01	0.03 ± 0.01	0.06 ± 0.006
**5**	MIC	0.06 ± 0.006	0.008 ± 0.0006	0.008 ± 0.0006	0.015 ± 0.003	0.004 ± 0.001	0.11 ± 0.01	0.015 ± 0.003	0.015 ± 0.003
	MFC	0.12 ± 0.04	0.015 ± 0.003	0.015 ± 0.003	0.03 ± 0.01	0.008 ± 0.0006	0.015 ± 0.003	0.03 ± 0.01	0.03 ± 0.01
**6**	MIC	0.06 ± 0.006	0.008 ± 0.0006	0.008 ± 0.0006	0.11 ± 0.01	0.008 ± 0.0006	0.008 ± 0.0006	0.015 ± 0.003	0.015 ± 0.003
	MFC	0.12 ± 0.04	0.015 ± 0.003	0.015 ± 0.003	0.03 ± 0.01	0.015 ± 0.003	0.015 ± 0.003	0.03 ± 0.01	0.03 ± 0.01
**7**	MIC	0.06 ± 0.006	0.008 ± 0.0006	0.008 ± 0.0006	0.008 ± 0.0006	0.004 ± 0.001	0.008 ± 0.0006	0.015 ± 0.003	0.015 ± 0.003
	MFC	0.12 ± 0.04	0.015 ± 0.003	0.015 ± 0.003	0.015 ± 0.003	0.008 ± 0.0006	0.015 ± 0.003	0.03 ± 0.01	0.03 ± 0.01
**8**	MIC	0.06 ± 0.006	0.015 ± 0.003	0.008 ± 0.0006	0.015 ± 0.003	0.11 ± 0.01	0.015 ± 0.003	0.015 ± 0.003	0.06 ± 0.006
	MFC	0.12 ± 0.04	0.03 ± 0.01	0.015 ± 0.003	0.03 ± 0.01	0.015 ± 0.003	0.03 ± 0.01	0.03 ± 0.01	0.12 ± 0.04
**9**	MIC	0.09 ± 0.003	0.015 ± 0.003	0.004 ± 0.001	0.015 ± 0.003	0.008 ± 0.0006	0.015 ± 0.003	0.015 ± 0.003	0.022 ± 0.005
	MFC	0.12 ± 0.04	0.03 ± 0.01	0.008 ± 0.0006	0.03 ± 0.01	0.015 ± 0.003	0.03 ± 0.01	0.03 ± 0.01	0.015 ± 0.003
**10**	MIC	0.06 ± 0.006	0.008 ± 0.0006	0.004 ± 0.001	0.008 ± 0.0006	0.004 ± 0.001	0.008 ± 0.0006	0.008 ± 0.0006	0.015 ± 0.003
	MFC	0.12 ± 0.04	0.015 ± 0.003	0.008 ± 0.0006	0.015 ± 0.003	0.008 ± 0.0006	0.015 ± 0.003	0.015 ± 0.003	0.03 ± 0.01
**11**	MIC	0.06 ± 0.006	0.015 ± 0.003	0.008 ± 0.0006	0.015 ± 0.003	0.008 ± 0.0006	0.015 ± 0.003	0.015 ± 0.003	0.015 ± 0.003
	MFC	0.12 ± 0.04	0.03 ± 0.01	0.015 ± 0.003	0.03 ± 0.01	0.015 ± 0.003	0.03 ± 0.01	0.03 ± 0.01	0.03 ± 0.01
**12**	MIC	0.06 ± 0.006	0.008 ± 0.0006	0.008 ± 0.0006	0.022 ± 0.005	0.008 ± 0.0006	0.015 ± 0.003	0.015 ± 0.003	0.03 ± 0.01
	MFC	0.12 ± 0.04	0.015 ± 0.003	0.015 ± 0.003	0.03 ± 0.01	0.015 ± 0.003	0.03 ± 0.01	0.03 ± 0.01	0.06 ± 0.006
**13**	MIC	0.06 ± 0.006	0.015 ± 0.003	0.008 ± 0.0006	0.015 ± 0.003	0.11 ± 0.01	0.015 ± 0.003	0.015 ± 0.003	0.015 ± 0.003
	MFC	0.12 ± 0.04	0.03 ± 0.01	0.015 ± 0.003	0.03 ± 0.01	0.03 ± 0.01	0.03 ± 0.01	0.03 ± 0.01	0.03 ± 0.01
**14**	MIC	0.06 ± 0.006	0.015 ± 0.003	0.015 ± 0.003	0.015 ± 0.003	0.11 ± 0.01	0.015 ± 0.003	0.015 ± 0.003	0.015 ± 0.003
	MFC	0.12 ± 0.04	0.03 ± 0.01	0.03 ± 0.01	0.03 ± 0.01	0.03 ± 0.01	0.03 ± 0.01	0.03 ± 0.01	0.03 ± 0.01
**15**	MIC	0.015 ± 0.003	0.008 ± 0.0006	0.008 ± 0.0006	0.008 ± 0.0006	0.008 ± 0.0006	0.008 ± 0.0006	0.008 ± 0.0006	0.008 ± 0.0006
	MFC	0.03 ± 0.01	0.015 ± 0.003	0.015 ± 0.003	0.015 ± 0.003	0.015 ± 0.003	0.015 ± 0.003	0.015 ± 0.003	0.015 ± 0.003
**16**	MIC	0.015 ± 0.003	0.015 ± 0.003	0.015 ± 0.003	0.015 ± 0.003	0.008 ± 0.0006	0.015 ± 0.003	0.015 ± 0.003	0.015 ± 0.003
	MFC	0.03 ± 0.01	0.03 ± 0.01	0.03 ± 0.01	0.03 ± 0.01	0.015 ± 0.003	0.03 ± 0.01	0.03 ± 0.01	0.03 ± 0.01
**17**	MIC	0.06 ± 0.006	0.015 ± 0.003	0.008 ± 0.0006	0.022 ± 0.005	0.11 ± 0.01	0.015 ± 0.003	0.015 ± 0.003	0.03 ± 0.01
	MFC	0.12 ± 0.04	0.03 ± 0.01	0.015 ± 0.003	0.015 ± 0.003	0.015 ± 0.003	0.03 ± 0.01	0.03 ± 0.01	0.06 ± 0.006
Ketoconazole	MIC	0.20 ± 0.01	0.20 ± 0.01	0.25 ± 0.05	0.20 ± 0.01	1.00 ± 0.1	2.50 ± 0.3	0.20 ± 0.01	0.20 ± 0.01
	MFC	0.50 ± 0.001	0.50 ± 0.002	0.50 ± 0.006	0.50 ± 0.004	1.50 ± 0.09	3.50 ± 0.03	0.50 ± 0.003	0.30 ± 0.01
Bifonazole	MIC	0.15 ± 0.05	0.10 ± 0.002	0.15 ± 0.05	0.15 ± 0.05	0.15 ± 0.05	0.20 ± 0.01	0.20 ± 0.01	0.10 ± 0.009
	MFC	0.20 ± 0.01	0.20 ± 0.01	0.20 ± 0.01	0.20 ± 0.01	0.20 ± 0.01	0.25 ± 0.05	0.25 ± 0.05	0.20 ± 0.01

*A.f.*—*A. fumigatus*, *A.v.*—*A. versicolor*, *A.o.*—*A. ochraceus*, *A.n.*—*A. niger*, *T.v.*—*T. viride*, *P.f.*—*P. funiculosum*, *P.o.*—*P. ochrochloron*, *P.v.c.*—*P. cyclpoium var verucosum.*

**Table 4:** Molecular docking free binding energies (kcal/mol) to antibacterial targets.

Comp.	Est. Binding Energy (kcal/mol)					Residues Involved in H-Bond Formation in *E. coli* MurB
	*E. coli* Gyrase 1KZN	Thymidylate Kinase 4QGG	*E. coli* Primase 1DDE	*E. coli* MurA JV4T	*E. coli* MurB 2Q85	
**1**	−4.15	−1.68	-	−5.63	−9.82	Arg213, Ser229
**2**	−3.17	-	-	−5.23	−10.04	Arg158, Ser229
**3**	−4.63	−2.55	−1.28	−6.27	−9.53	Arg213, Ser229
**4**	−5.10	-	-	−4.63	−7.90	Arg158, Arg213
**5**	−3.51	-	-	−5.20	−8.97	Arg213, Ser229
**6**	−4.15	-	−1.39	−4.82	−7.68	Arg213
**7**	−4.38	-	-	−6.12	−9.14	Arg158, Ser229
**8**	−5.37	−2.85	-	−5.92	−10.88	Ser50, Ser229
**9**	−4.12	-	-	−5.37	−8.11	Ser50, Arg158
**10**	−5.47	-	-	−5.42	−7.54	Ser50
**11**	−5.21	−3.47	−2.61	−6.20	−10.46	Ser116, Ser229
**12**	−3.50	−2.51	−	−5.73	−9.56	Ser229
**13**	−4.39	-	−	−5.14	−8.55	Ser229
**14**	−4.26	-	−1.29	−5.32	−8.42	Ser229
**15**	−3.94	-	−2.57	−5.18	−7.60	Arg213
**16**	−5.28	-	-	−4.92	−8.30	Ser229
**17**	−4.36	-	-	−5.16	−9.46	Arg158, Ser229

**Table 5:** Molecular docking free binding energies (kcal/mol) to antifungal targets.

	Est. Binding Energy (kcal/mol)		Residues Involved in H-Bond Formation	Residues Involved in Hydrophobic Interactions	Residues Involved in Aromatic Interactions	Interactions with HEM601
Comp.	DNA TopoIV 1S16	CYP51 of *C. albicans* 5V5Z				
**1**	−3.69	−7.54	-	Tyr118, Thr311, Phe380, Met508, Hem601	-	Hydrophobic
**2**	−3.18	−9.70	Tyr132	Tyr118, Leu300, Ile304, Thr311, Hem601	Tyr118	Hydrophobic
**3**	−3.25	−12.34	Tyr132	Tyr118,Ile131, Ile304, Hem601	Hem601	Hydrophobic, aromatic, Fe binding
**4**	−2.66	−8.70	-	Tyr118, Leu300, Thr311, Leu376, Phe380, Met508, Hem601	Tyr118, Hem601	Hydrophobic, aromatic
**5**	−4.25	−9.34	Tyr118	Tyr118, Leu376, Met508, Hem601	Tyr118	Hydrophobic
**6**	−3.14	−9.65	Tyr64	Tyr118, Tyr122, Thr311, Leu376, Phe380, Hem601	Tyr118	Hydrophobic
**7**	−4.17	−9.82	Tyr132	Tyr118, Leu121, Thr311, Phe380, Met508, Hem601	Hem601	Hydrophobic, aromatic
**8**	−2.59	−7.11	-	Tyr118, Leu376, Met508, Hem601	-	Hydrophobic
**9**	−4.39	−9.54	Tyr132	Tyr118, Phe380, Met508, Hem601	-	Hydrophobic
**10**	−2.67	−10.11	Tyr64	Tyr118, Leu300, Thr311, Met508, Hem601	Tyr118, Hem601	Hydrophobic, aromatic
**11**	−2.73	−9.31	Tyr118	Leu300, Met508, Hem601	Hem601	Hydrophobic, aromatic
**12**	−4.37	−8.62	-	Tyr118, Tyr122, Thr311, Met508, Hem601	Tyr122, Hem601	Hydrophobic, aromatic
**13**	−3.56	−8.81	-	Tyr118, Tyr122, Thr311, Leu376, Phe380, Hem601	Tyr118	Hydrophobic
**14**	−3.28	−7.64	-	Tyr118, Thr311, Leu376, Met508, Hem601	-	Hydrophobic
**15**	−3.10	−12.95	Thr311	Ile131, Leu300, Ile304, Hem601	Hem601	Hydrophobic, aromatic, Fe binding
**16**	−2.67	−10.26	Tyr132	Tyr118, Tyr122, Leu300, Ile304, Hem601	Hem601	Hydrophobic, aromatic
**17**	−2.57	−7.96	-	Tyr118, Tyr122, Thr311, Leu376, Met508, Hem601	Tyr118, Hem601	Hydrophobic, aromatic
Ketoconazole	-	−8.23	Tyr64	Tyr118, Ile131, Tyr132, Leu300, Ile304, Leu376, Met508, Hem601	Hem601	Hydrophobic, aromatic

**Table 6:** Drug-likeness predictions of tested compounds.

Comp.	MW ^a^ (≤500)	HBAs ^b^ (≤10)	HBDs ^c^ (≤5)	Lipophilicity (ClogP) ^d^ (≤5)	RBs ^e^ (≤10)	TPSA ^f^ (≤140)	Lipinski Violations	Bioavailability Score	Drug-Likeness Score
**1**	432.51	5	2	3.30	9	157.09	0	0.55	−0.14
**2**	438.48	5	2	3.45	5	157.09	0	0.11	−0.64
**3**	410.47	4	2	3.46	4	140.02	0	0.55	−0.78
**4**	410.47	4	2	3.47	4	140.02	0	0.55	−0.81
**5**	445.56	5	1	2.86	7	132.26	0	0.55	+0.24
**6**	403.48	5	1	2.48	4	132.26	0	0.55	−0.38
**7**	398.46	4	1	3.28	5	132.93	0	0.55	−0.69
**8**	436.48	6	2	3.31	6	157.09	0	0.55	−0.70
**9**	422.45	6	2	2.99	7	157.09	0	0.11	−0.37
**10**	428.46	5	2	3.78	4	140.02	0	0.55	−0.77
**11**	376.41	5	2	2.11	5	157.09	0	0.55	−0.71
**12**	418.49	5	2	3.02	6	157.09	0	0.11	−0.24
**13**	442.48	5	1	4.15	5	129.02	0	0.55	−0.95
**14**	408.42	6	2	2.65	6	157.09	0	0.11	−0.40
**15**	350.39	4	1	3.02	3	119.79	0	0.55	−0.15
**16**	421.47	6	1	2.79	4	132.26	0	0.55	−0.14
**17**	390.43	5	2	2.35	6	157.09	0	0.11	−0.59

^a^ Molecular weight; ^b^ number of hydrogen bond acceptors; ^c^ number of hydrogen bond donors; ^d^ consensus logarithm of partition coefficient (octanol/water) predicted as an average of five methods (iLOGP, XLOGP3, WLOGP, MLOGP, SILICOS-IT); ^e^ rotatable bonds; ^f^ topological polar surface area (Å).

**Table 7:** Absorption and distribution properties for the examined compounds **1**–**17**.

No	Absorption								Distribution				
	Peff ^a^	MDCK ^b^	Sw ^c^	SpH ^d^	FaSSGF ^e^	FaSSIF ^f^	FeSSIF ^g^	Absorption Risk	BBB ^h^	LogBBB ^i^	fu% ^j^	Vd ^k^	RBP ^l^
**1**	1.642	18.188	23	10.009	0.009	0.067	0.160	0.000	Low (59%)	−1.231	4.588	0.328	0.661
**2**	2.232	112.633	5	1.295	0.008	0.040	0.115	1.000	Low (66%)	−0.793	4.446	0.244	0.692
**3**	2.491	160.415	2	0.002	0.026	0.010	0.155	1.000	High (89%)	−0.071	4.548	0.691	0.738
**4**	2.439	192.624	2	0.002	0.026	0.008	0.111	1.000	High (88%)	−0.127	4.449	0.742	0.737
**5**	1.979	244.487	30	0.049	0.914	0.031	0.089	0.000	High (99%)	−0.006	9.925	1.232	0.748
**6**	2.608	289.456	8	0.008	0.124	0.071	0.197	0.329	High (99%)	−0.130	9.631	1.097	0.767
**7**	2.846	695.382	2	0.002	0.013	0.013	0.159	1.000	High (94%)	−0.393	5.233	0.780	0.744
**8**	1.713	20.994	28	6.904	0.009	0.035	0.381	0.000	Low (84%)	−0.964	5.525	0.325	0.680
**9**	1.824	11.524	37	14.568	0.014	0.090	0.207	0.000	Low (66%)	−1.151	5.491	0.328	0.677
**10**	2.552	238.505	2	0.002	0.024	0.005	0.104	1.000	High (77%)	−0.046	4.722	0.746	0.741
**11**	1.557	18.324	37	7.429	0.017	0.092	0.380	0.000	Low (74%)	−1.047	5.954	0.313	0.684
**12**	1.624	16.857	29	7.179	0.010	0.051	0.308	0.000	Low (84%)	−1.056	5.402	0.327	0.677
**13**	3.755	512.700	1	0.001	0.018	0.006	0.085	1.918	High (96%)	0.129	4.768	0.861	0.731
**14**	1.864	39.838	38	13.699	0.014	0.178	0.322	0.000	Low (90%)	−0.997	5.963	0.313	0.683
**15**	3.536	572.262	4	0.004	0.099	0.018	0.211	1.000	High (99%)	0.137	9.910	0.779	0.783
**16**	3.229	352.377	8	0.008	0.122	0.061	0.169	0.370	High (99%)	−0.037	10.236	1.141	0.769
**17**	1.652	29.583	38	13.731	0.015	0.236	0.260	0.000	Low (84%)	−1.085	5.838	0.314	0.680

^a^ Human jejunal effective permeability (10^4^ cm/s); ^b^ MDCK Transwell permeability (10^7^ cm/s); ^c^ water solubility (μg/mL); ^d^ water solubility in mg/mL at pH 7.4; ^e^ solubility expressed in mg/mL in fast state simulated gastric fluid; ^f^ solubility expressed in mg/mL in fast state simulated intestinal fluid; ^g^ solubility expressed in mg/mL in fed state simulated intestinal fluid; ^h^ possibility to penetrate blood–brain barrier; ^i^ logarithm of the brain/blood partition coefficient; ^j^ human %fraction unbound; ^k^ human volume of distribution in steady state (L/kg); ^l^ blood-to-plasma ratio in humans.

**Table 8:** Studies on the metabolism characteristics of compounds **1**–**17**.

No	Metabolism													
	CYP 1A2 Inh.	CYP1A2 Substr. K_m_	CYP 2C9 Inh	CYP2C9 Substr. K_m_	CYP 2C19 Inh.	CYP2C19 Substr. K_m_	CYP 2D6 Inh.	CYP2D6 Substr. K_m_	CYP 3A4 Inh.	CYP3A4 Substr. K_m_	CYP CL_int_^a^	HEP CL_int_^b^	UGTs Subs. ^c^	CYP Risk
**1**	No	Non Subs.	No	86.146	No	24.703	No	Non Subs.	No	Non Subs.	6.568	11.362	1A3, 1A9	0.000
**2**	No	23.682	No	8.127	No	39.264	No	Non Subs.	No	Non Subs.	100.398	16.359	1A8, 1A9, 1A10	2.000
**3**	No	30.916	No	14.403	No	29.495	No	127.051	Yes	Non Subs.	65.013	71.146	1A1, 1A8, 1A9, 1A10, 2B15	2.372
**4**	No	29.485	No	8.423	No	93.004	No	204.398	Yes	Non Subs.	80.365	89.557	1A1, 1A8, 1A9, 1A10, 2B15	2.985
**5**	No	18.129	No	83.216	No	20.465	No	13.006	Yes	22.767	78.710	23.739	1A4, 1A9	1.147
**6**	No	7.486	No	87.569	No	44.972	No	28.012	Yes	31.235	101.603	18.418	Non Subs.	1.000
**7**	No	26.192	No	15.906	No	4.882	No	38.409	Yes	21.943	89.261	21.857	1A9	0.981
**8**	No	Non Subs.	No	20.918	No	203.738	No	Non Subs.	No	Non Subs.	19.358	7.088	1A3, 1A8, 1A9, 1A10	0.935
**9**	No	Non Subs.	No	123.319	No	16.370	No	Non Subs.	Yes	Non Subs.	6.868	8.300	1A3, 1A9	0.000
**10**	No	34.151	No	7.744	No	94.454	No	255.204	Yes	Non Subs.	131.921	93.639	1A1, 1A8, 1A9, 1A10, 2B15	3.000
**11**	No	Non Subs.	No	36.930	No	220.331	No	Non Subs.	No	Non Subs.	9.918	5.080	1A3, 1A8, 1A9	0.000
**12**	No	Non Subs.	No	25.953	No	199.456	No	Non Subs.	No	Non Subs.	12.348	6.689	1A3, 1A8, 1A9	0.234
**13**	No	3.514	No	15.364	No	66.722	No	27.176	Yes	11.550	259.955	80.554	1A1, 1A9	3.228
**14**	No	Non Subs.	No	96.500	No	5.165	No	Non Subs.	Yes	Non Subs.	13.286	6.370	1A3, 1A9	0.000
**15**	No	20.385	No	167.240	No	60.458	No	91.889	Yes	130.324	57.430	21.782	1A1, 1A9	0.740
**16**	No	9.212	No	64.353	No	48.228	No	32.873	Yes	24.123	102.341	18.506	1A8, 1A9	1.055
**17**	No	Non Subs.	No	100.364	No	4.815	No	Non Subs.	Yes	Non Subs.	10.276	5.966	1A3, 1A9	0.000

^a^ A total intrinsic human cytochrome P-450 mediated clearance based on the sum of CYP1A2, CYP2C9, CYP2C19, CYP2D6, and CYP3A4 individual clearances; ^b^ human hepatocytes intrinsic clearance (μL/min/10^6^ cells); ^c^ potential isoenzyme substrates of UDP-glucuronosyltransferases (UGTs).

**Table 9:** Studies on selected transporters for the examined compounds **1**–**17**.

No	Pgp Sub.	Pgp Inh.	BCRP Sub.	BCRP Inh.	BSEP Inh.
**1**	Yes	No (49%)	Yes	No	No
**2**	Yes	Yes (62%)	Yes	No	No
**3**	Yes	Yes (97%)	Yes	No	No
**4**	Yes	Yes (97%)	Yes	No	No
**5**	Yes	Yes (88%)	Yes	No	No
**6**	Yes	Yes (60%)	Yes	No	No
**7**	Yes	Yes (97%)	Yes	No	No
**8**	Yes	No (93%)	Yes	No	No
**9**	Yes	No (49%)	Yes	No	No
**10**	Yes	Yes (97%)	Yes	No	No
**11**	Yes	No (93%)	Yes	No	No
**12**	Yes	No (93%)	No	No	No
**13**	Yes	Yes (97%)	Yes	No	No
**14**	Yes	No (68%)	Yes	No	No
**15**	Yes	No (46%)	Yes	No	No
**16**	Yes	Yes (57%)	Yes	No	No
**17**	Yes	No (78%)	Yes	No	No

## Data Availability

Not applicable.

## Supplementary Materials

The following supporting information can be downloaded at: https://www.mdpi.com/article/10.3390/ph16010131/s1, Table S1. Prediction of organ toxicity and toxicity end points for compounds **1**–**17**, Table S2. Prediction of adverse outcomes according to TOX21 of compounds **1**–**17**, ^1^H-NMR and ^13^C-NMR spectra of compounds.
