# Supplementary material for: N-Derivatives of (Z)-Methyl 3-(4-Oxo-2-thioxothiazolidin-5-ylidene)methyl)-1H-indole-2-carboxylates as Antimicrobial Agents—In Silico and In Vitro Evaluation
Source: Pharmaceuticals (Basel). 2023 Jan 16;16(1):131. doi: 10.3390/ph16010131 (PMC9865890; doi:10.3390/ph16010131)
Supplement: Supplementary file 1 [file pharmaceuticals-16-00131-s001.zip › pharmaceuticals-2111021-supplementary.pdf]

Article

N-Derivatives of (Z)-methyl 3-(4-oxo-2-thioxothiazolidin-5-ylidene) methyl)-1*H*-indole-2-carboxylates as antimicrobial agents. *In silico* and *in vitro* evaluation.

Anthi Petrou[1], Athina Geronikaki[1*], Victor Kartsev[2], Antonis Kousaxidis[1], Aliki Papadimitriou-Tsantarliotou[3], Marina Kostic[4], Marija Ivanov[4], Marina Sokovic[4], Ioannis Nikolaou[1], Ioannis S. Vizirianakis[3, 5]

[1] Department of Pharmaceutical Chemistry, School of Pharmacy, Aristotle University of Thessaloniki, 54124 Thessaloniki, Greece
[2] InterBioScreen, 142432 Chernogolovka, Moscow Region, Russia
[3] Laboratory of Pharmacology, School of Pharmacy, Aristotle University of Thessaloniki, Thessaloniki, Greece
[4]Mycological Laboratory, Department of Plant Physiology, Institute for Biological Research, Siniša, Stankovic-National Institute of Republic of Serbia, University of Belgrade, Bulevar Despota Stefana 142, 11000 Belgrade, Serbia
[5]Department of Life and Health Sciences, University of Nicosia, Nicosia, Cyprus
* Correspondence: geronik@pharm.auth.gr

Table S1. Prediction of organ toxicity and toxicity end points for compounds **1-17**.

**Comp.**	**Organ toxicity**			**Toxicity end points**			
	**Cardiotoxicity**	**Hepatotoxicity**	**Carcinogenicity**	**Immunotoxicity**	**Mutagenicity**	**Cytotoxicity**	**Phospholipidosis**
**1**	Inactive (0.79)	Inactive (0.68)	Inactive (0.62)	Inactive(0.55)	Inactive (0.74)	Inactive(0.64)	Inactive (0.99)
**2**	Inactive (0.75)	Active (0.56)	Inactive (0.63)	Inactive (0.97)	Inactive (0.55)	Inactive (0.64)	Inactive (0.89)
**3**	Inactive (0.93)	Active (0.59)	Inactive (0.61)	Active (0.61)	Inactive (0.54)	Inactive(0.61)	Inactive (0.89)
**4**	Inactive (0.93)	Active (0.60)	Inactive (0.60)	Active(0.72)	Inactive (0.53)	Inactive(0.64)	Inactive (0.89)
**5**	Inactive (0.60)	Inactive (0.66)	Inactive (0.57)	Active (0.88)	Inactive (0.69)	Inactive (0.65)	Inactive (0.81)
**6**	Inactive (0.93)	Active (0.52)	Inactive (0.56)	Inactive (0.76)	Inactive (0.58)	Inactive (0.65)	Inactive (0.93)
**7**	Inactive (0.84)	Inactive (0.50)	Inactive (0.58)	Inactive (0.76)	Inactive (0.61)	Inactive (0.62)	Inactive (0.89)
**8**	Inactive (0.84)	Active (0.55)	Inactive (0.66)	Active (0.64)	Inactive (0.64)	Inactive (0.70)	Inactive (0.99)
**9**	Inactive (0.84)	Active (0.51)	Inactive (0.62)	Active (0.77)	Inactive (0.71)	Inactive (0.63)	Inactive (0.99)
**10**	Inactive (0.93)	Active (0.59)	Inactive (0.62)	Active (0.84)	Inactive (0.58)	Inactive (0.67)	Inactive (0.89)
**11**	Inactive (0.84)	Inactive (0.53)	Inactive (0.63)	Inactive (0.82)	Inactive (0.69)	Inactive (0.64)	Inactive (0.99)
**12**	Inactive (0.84)	Inactive (0.54)	Inactive (0.68)	Inactive (0.88)	Inactive (0.63)	Inactive (0.67)	Inactive (0.99)
**13**	Inactive (0.93)	Active (0.57)	Inactive (0.60)	Active (0.98)	Inactive (0.59)	Inactive (0.70)	Inactive (0.81)
**14**	Inactive (0.84)	Inactive (0.52)	Inactive (0.63)	Active (0.78)	Inactive (0.70)	Inactive (0.63)	Inactive (0.99)
**15**	Inactive (0.93)	Active (0.56)	Inactive (0.63)	Inactive (0.54)	Inactive (0.58)	Inactive (0.73)	Inactive (0.89)
**16**	Inactive (0.93)	Active (0.51)	Inactive (0.59)	Active (0.81)	Inactive (0.57)	Inactive (0.65)	Inactive (0.93)
**17**	Inactive (0.79)	Active (0.59)	Inactive (0.63)	Active (0.79)	Inactive (0.71)	Inactive (0.63)	Inactive (0.99)

Number in brackets indicate possibilites. Cardiotoxicity is reffered to the probability of inhibition of the inward rectifying voltage gated potassium channel in the heart encoded by the human ether-a-go-go related gene (hERG).

Table S2. Prediction of adverse outcomes according to TOX21 of compounds **1-17**.

Comp.	Tox21-Nuclear receptor signalling pathways							Tox21-Stress response pathways				
	**AhR**	**AR**	**AR-LBD**	**ARO**	**ER**	**ER-LBD**	**PPARγ**	**NRF2/ARE**	**HSE**	**MMP**	**p53**	**ATAD5**
**1**	Inactive (0.77)	Inactive (0.98)	Inactive (0.97)	Inactive (0.93)	Inactive (0.87)	Inactive (0.95)	Inactive (0.81)	Inactive (0.85)	Inactive (0.85)	Inactive (0.85)	Inactive (0.81)	Inactive (0.90)
**2**	Inactive (0.61)	Inactive (0.95)	Inactive (0.97)	Inactive (0.81)	Inactive (0.76)	Inactive (0.92)	Inactive (0.81)	Inactive (0.90)	Inactive (0.90)	Inactive (0.54)	Inactive (0.75)	Inactive (0.71)
**3**	Inactive (0.60)	Inactive (0.92)	Inactive (0.97)	Inactive (0.84)	Inactive (0.73)	Inactive (0.92)	Inactive (0.82)	Inactive (0.89)	Inactive (0.89)	Inactive (0.51)	Inactive (0.71)	Inactive (0.75)
**4**	Inactive (0.63)	Inactive (0.94)	Inactive (0.97)	Inactive (0.83)	Inactive (0.72)	Inactive (0.90)	Inactive (0.81)	Inactive (0.91)	Inactive (0.91)	Inactive (0.51)	Inactive (0.70)	Inactive (0.75)
**5**	Inactive (0.78)	Inactive (0.95)	Inactive (0.93)	Inactive (0.91)	Inactive (0.87)	Inactive (0.94)	Inactive (0.82)	Inactive (0.91)	Inactive (0.91)	Inactive (0.75)	Inactive (0.82)	Inactive (0.94)
**6**	Inactive (0.82)	Inactive (0.95)	Inactive (0.95)	Inactive (0.90)	Inactive (0.87)	Inactive (0.94)	Inactive (0.88)	Inactive (0.92)	Inactive (0.92)	Inactive (0.69)	Inactive (0.89)	Inactive (0.94)
**7**	Inactive (0.65)	Inactive (0.98)	Inactive (0.96)	Inactive (0.93)	Inactive (0.84)	Inactive (0.95)	Inactive (0.84)	Inactive (0.91)	Inactive (0.91)	Inactive (0.79)	Inactive (0.81)	Inactive (0.83)
**8**	Inactive (0.63)	Inactive (0.97)	Inactive (0.97)	Inactive (0.87)	Inactive (0.79)	Inactive (0.88)	Inactive (0.87)	Inactive (0.89)	Inactive (0.89)	Inactive (0.65)	Inactive (0.79)	Inactive (0.91)
**9**	Inactive (0.78)	Inactive (0.98)	Inactive (0.97)	Inactive (0.90)	Inactive (0.84)	Inactive (0.90)	Inactive (0.79)	Inactive (0.85)	Inactive (0.85)	Inactive (0.82)	Inactive (0.82)	Inactive (0.87)
**10**	Inactive (0.65)	Inactive (0.96)	Inactive (0.96)	Inactive (0.87)	Inactive (0.71)	Inactive (0.83)	Inactive (0.78)	Inactive (0.89)	Inactive (0.89)	Inactive (0.54)	Inactive (0.73)	Inactive (0.82)
**11**	Inactive (0.72)	Inactive (0.98)	Inactive (0.97)	Inactive (0.93)	Inactive (0.89)	Inactive (0.95)	Inactive (0.80)	Inactive (0.86)	Inactive (0.86)	Inactive (0.83)	Inactive (0.84)	Inactive (0.79)
**12**	Inactive (0.64)	Inactive (0.97)	Inactive (0.97)	Inactive (0.91)	Inactive (0.82)	Inactive (0.92)	Inactive (0.88)	Inactive (0.90)	Inactive (0.90)	Inactive (0.67)	Inactive (0.79)	Inactive (0.89)
**13**	Inactive (0.63)	Inactive (0.95)	Inactive (0.95)	Inactive (0.88)	Inactive (0.72)	Inactive (0.88)	Inactive (0.86)	Inactive (0.89)	Inactive (0.89)	Inactive (0.53)	Inactive (0.75)	Inactive (0.84)
**14**	Inactive (0.78)	Inactive (0.98)	Inactive (0.96)	Inactive (0.89)	Inactive (0.84)	Inactive (0.89)	Inactive (0.81)	Inactive (0.86)	Inactive (0.86)	Inactive (0.82)	Inactive (0.83)	Inactive (0.86)
**15**	Inactive (0.58)	Inactive (0.97)	Inactive (0.96)	Inactive (0.89)	Inactive (0.80)	Inactive (0.93)	Inactive (0.89)	Inactive (0.91)	Inactive (0.91)	Inactive (0.65)	Inactive (0.77)	Inactive (0.88)
**16**	Inactive (0.84)	Inactive (0.97)	Inactive (0.93)	Inactive (0.91)	Inactive (0.80)	Inactive (0.90)	Inactive (0.90)	Inactive (0.91)	Inactive (0.91)	Inactive (0.73)	Inactive (0.89)	Inactive (0.93)
**17**	Inactive (0.79)	Inactive (0.98)	Inactive (0.96)	Inactive (0.94)	Inactive (0.89)	Inactive (0.95)	Inactive (0.80)	Inactive (0.89)	Inactive (0.89)	Inactive (0.85)	Inactive (0.81)	Inactive (0.84)

(1) Aryl hydrocarbon Receptor, (2) Androgen Receptor, (3) Androgen Receptor Ligand Binding Domain, (4) Aromatase, (5) Estrogen Receptor Alpha, (6) Estrogen Receptor Ligand Binding Domain, (7) Peroxisome Proliferator Activated Receptor Gamma, (8) Nuclear factor (erythroid-derived 2)-like 2/antioxidant responsive element, (9) Heat shock factor response element, (10) Heat shock factor response element, (11) Phosphoprotein (Tumor Suppressor) p53, (12) ATPase family AAA domain-containing protein 5

^{1}H-NMR and ^{13}C-NMR of compounds

Compound 1

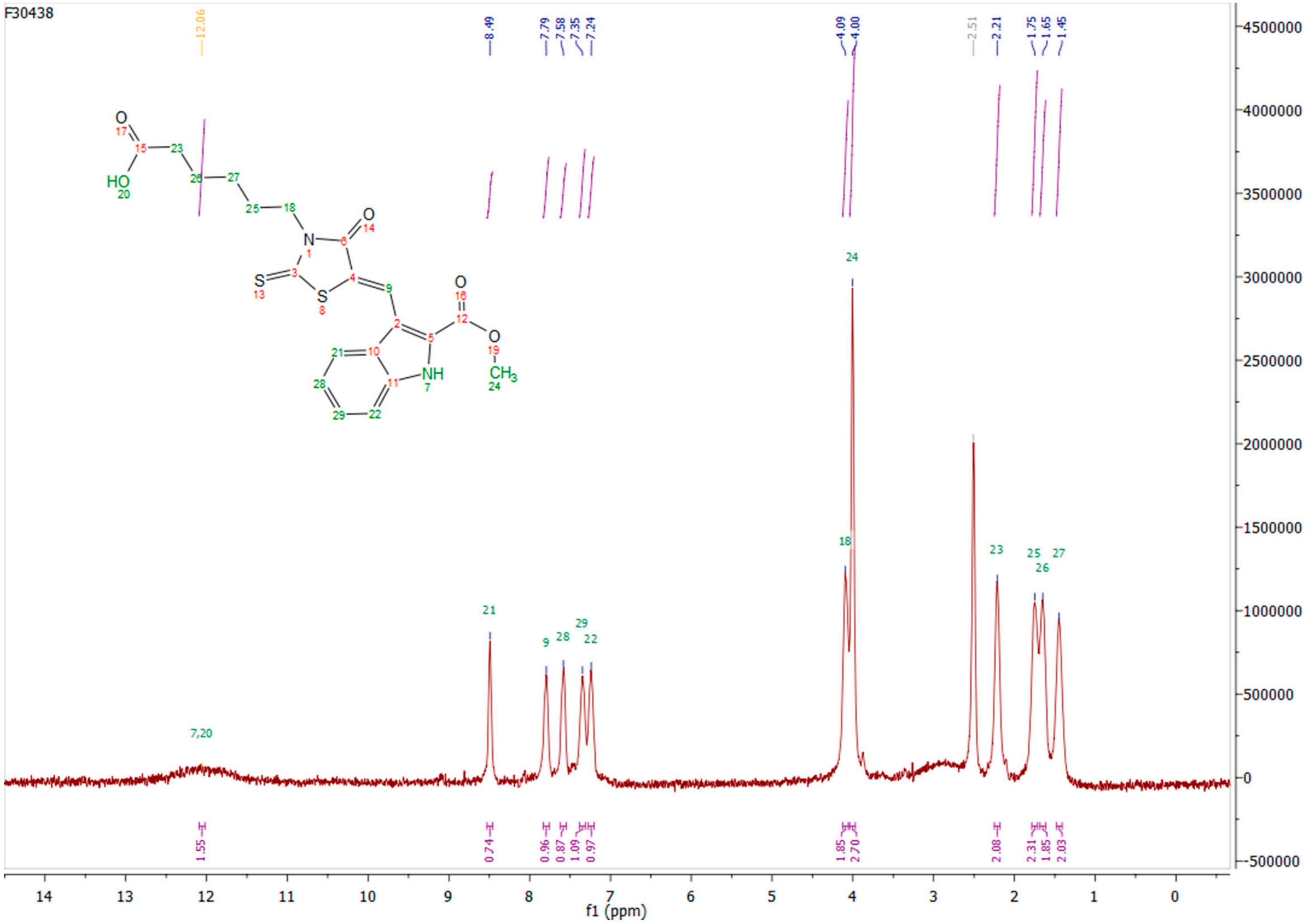

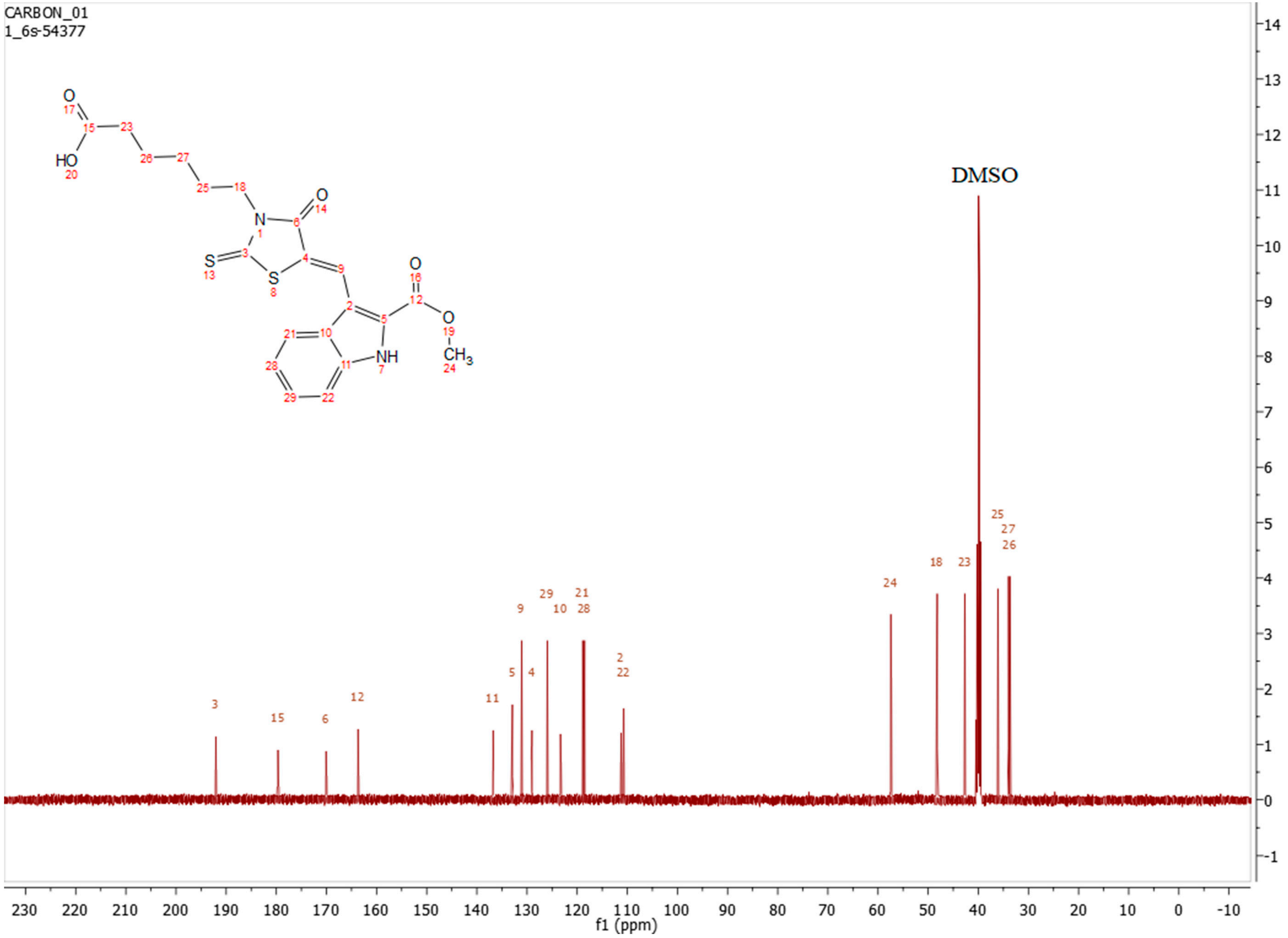
CARBON_01
1_6s-54377
DMSO
f1 (ppm)

Compound 2

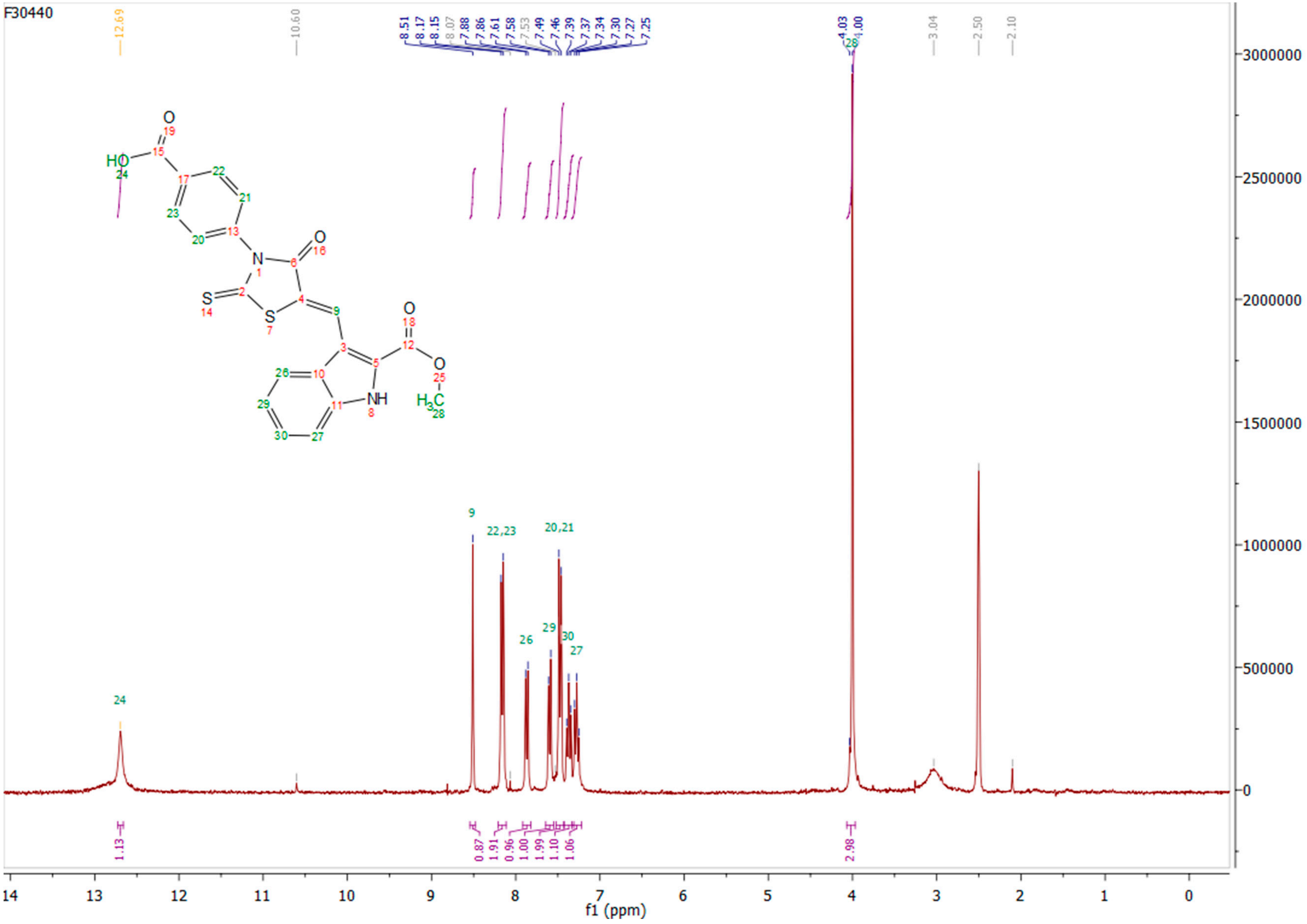

CARBON_01
2_45217

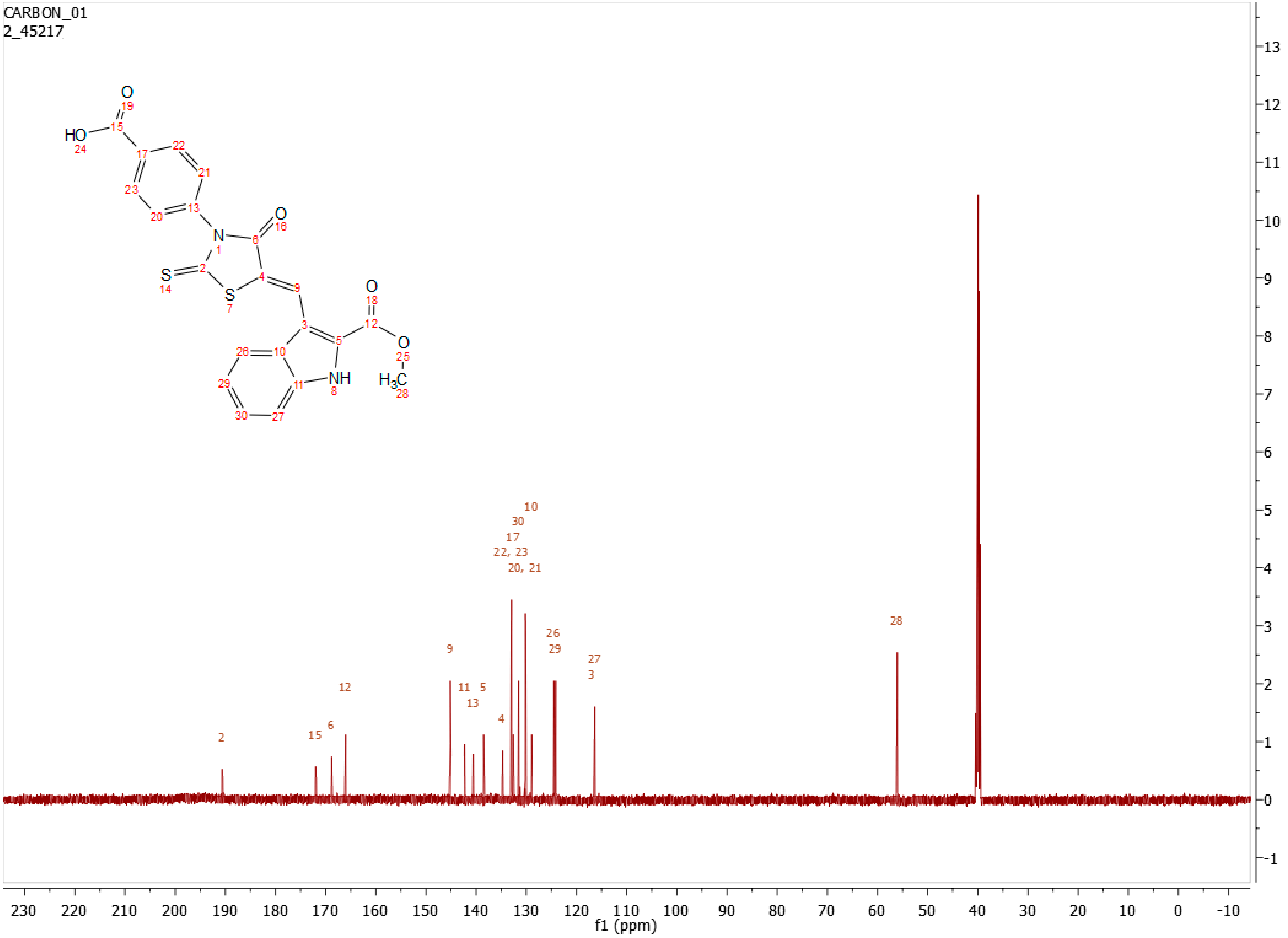

Compound 3

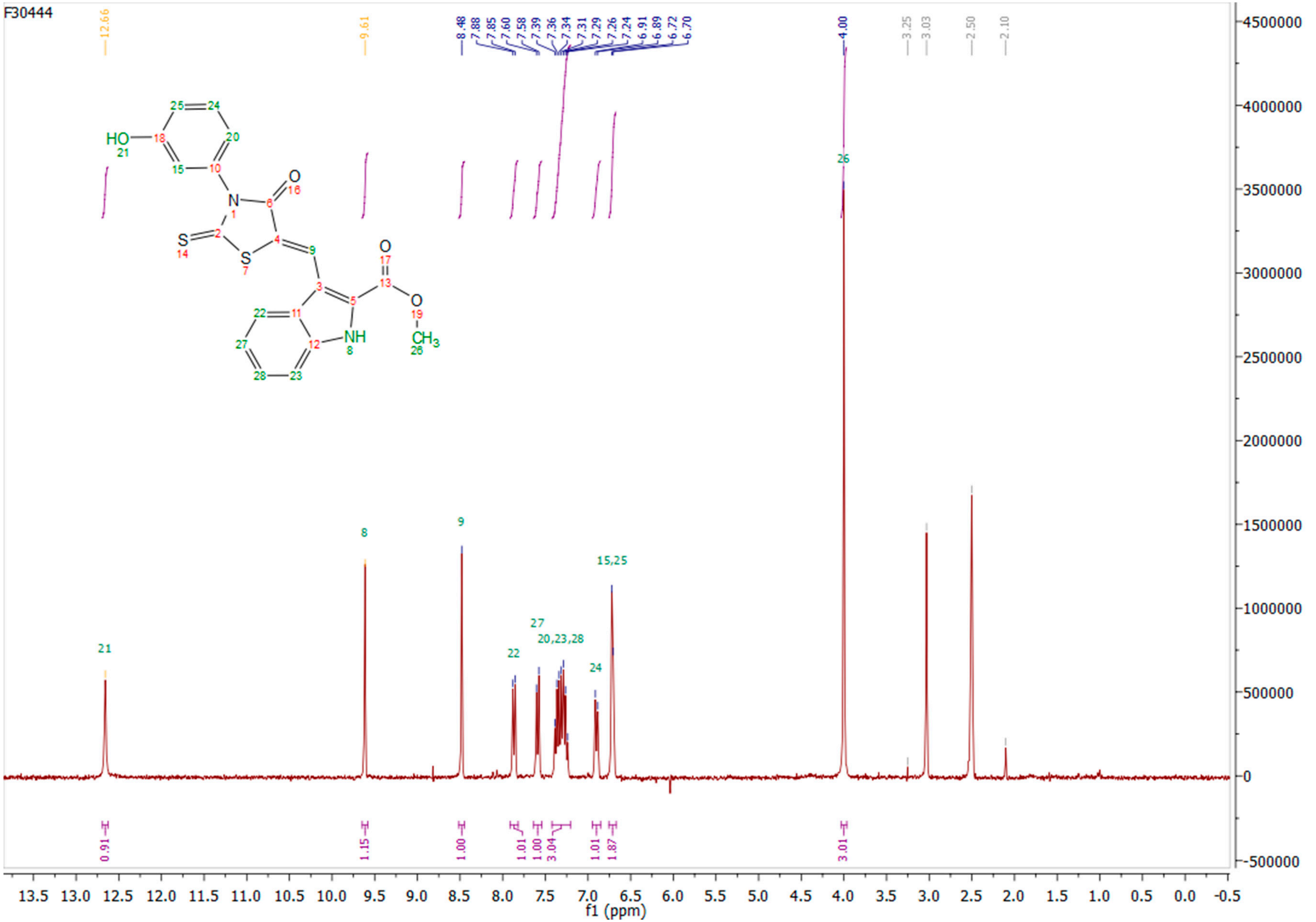

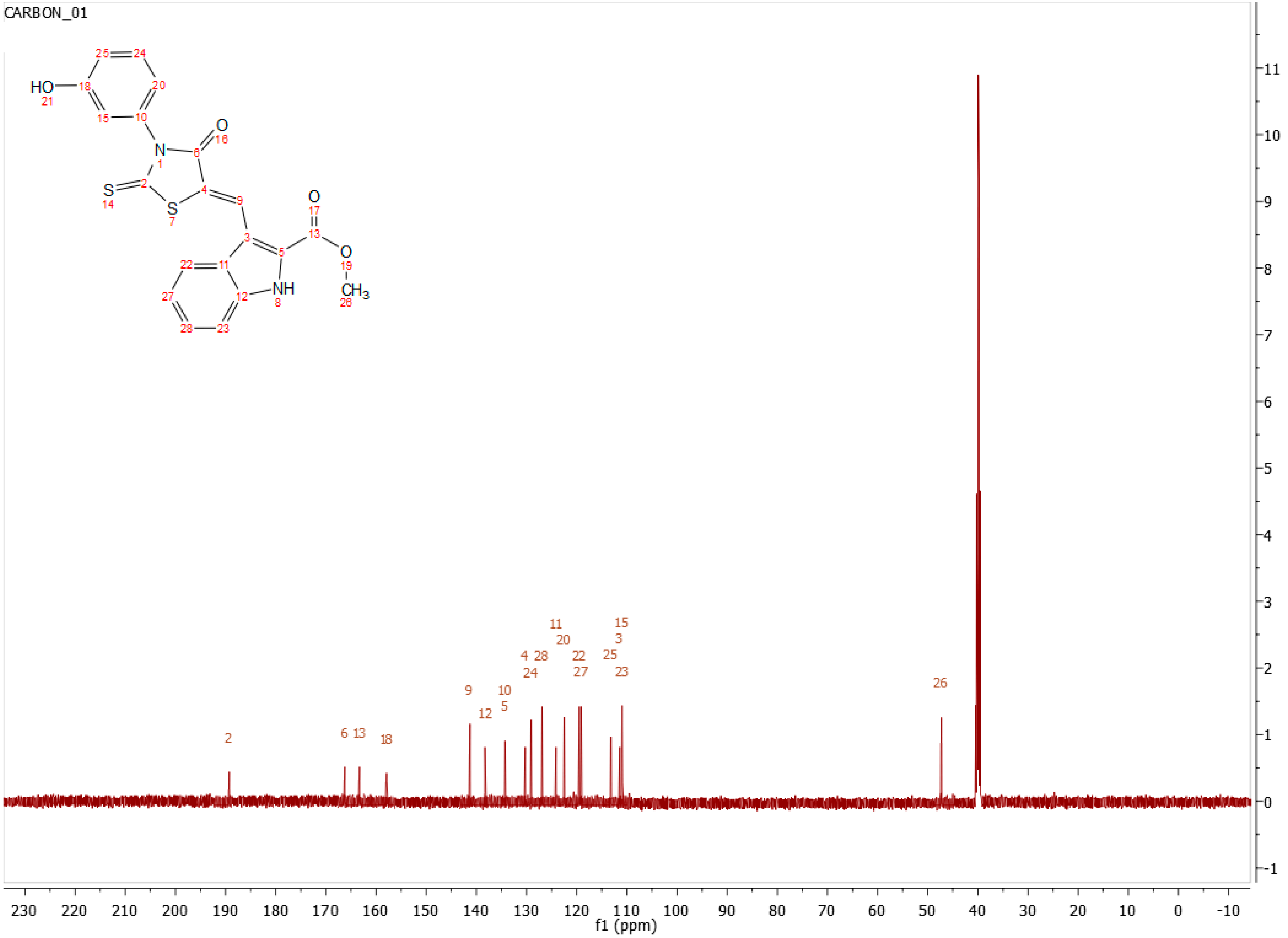
CARBON_01
HO
21
O
16
S
14
S
7
O
17
O
19
NH
8
CH3
26
2
6 13
18
9
12
10
5
4
24
28
11
20
22
27
15
3
25
23
26
f1 (ppm)

Compound 4

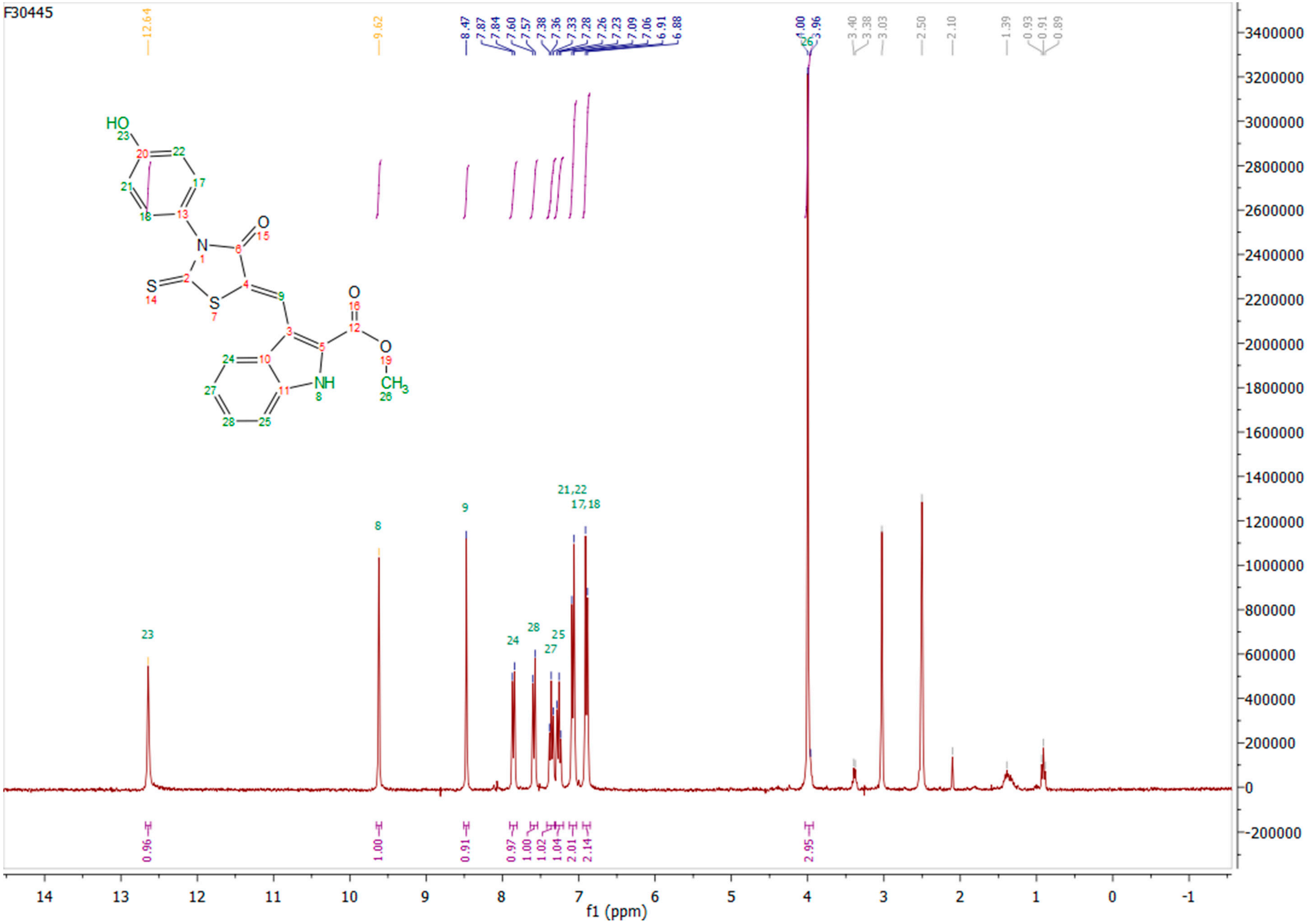
F30445
12.64
9.62
8.47
7.87
7.84
7.60
7.57
7.38
7.36
7.33
7.28
7.26
7.23
7.09
7.06
6.91
6.88
4.00
3.96
3.40
3.38
3.03
2.50
2.10
1.39
0.93
0.91
0.89
HO
NH
CH3
23
8
9
24
28
25
27
21,22
17,18
26
0.96
1.00
0.91
0.97
1.00
1.02
1.04
2.01
2.14
2.95
f1 (ppm)

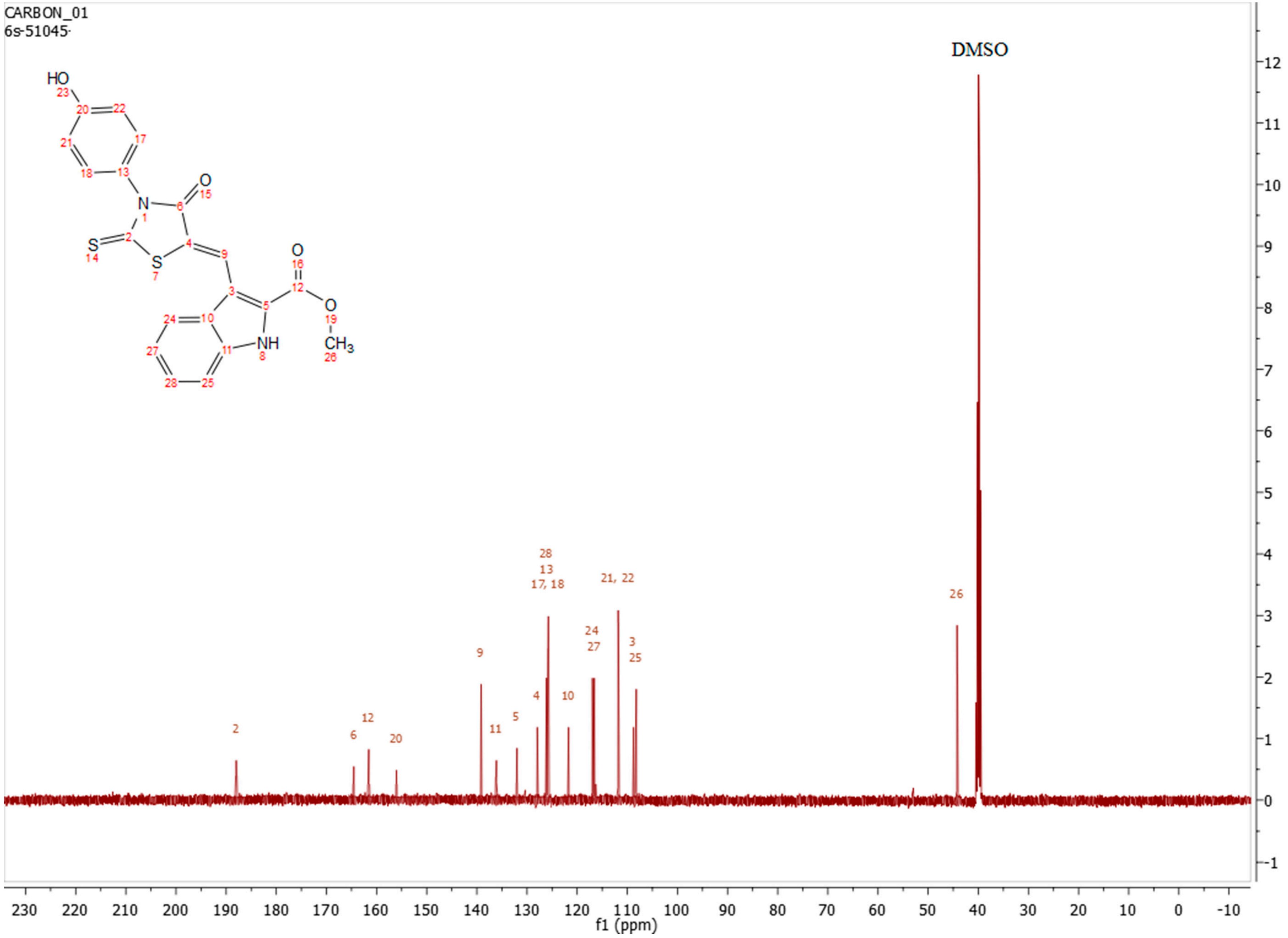
CARBON_01
6s-51045
HO
N
S
S
O
O
O
NH
CH3
DMSO
2
6
12
20
9
11
5
4
28
13
17, 18
10
24
27
21, 22
3
25
26
f1 (ppm)

Compound 5

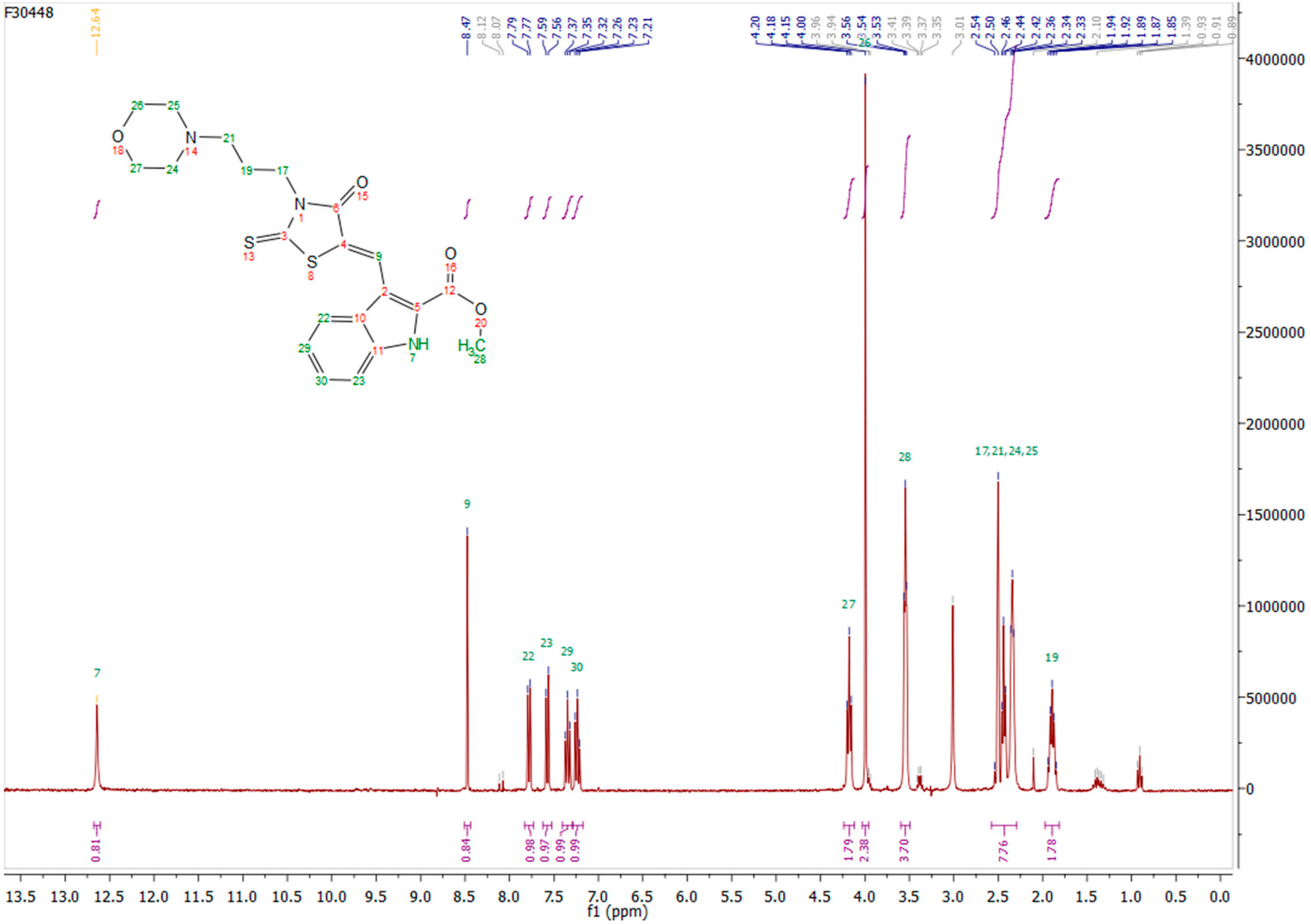
F30448
12.64
8.47
8.12
8.07
7.79
7.77
7.59
7.56
7.37
7.35
7.32
7.26
7.23
7.21
4.20
4.18
4.15
4.00
3.96
3.94
3.56
3.54
3.53
3.41
3.39
3.37
3.35
3.01
2.54
2.50
2.46
2.44
2.42
2.36
2.34
2.33
2.10
1.94
1.92
1.89
1.87
1.85
1.39
0.93
0.91
0.89
7
9
22
23
29
30
27
26
28
17,21,24,25
19
0.81
0.84
0.98
0.97
0.99
0.99
1.79
2.38
3.70
7.76
1.78
f1 (ppm)

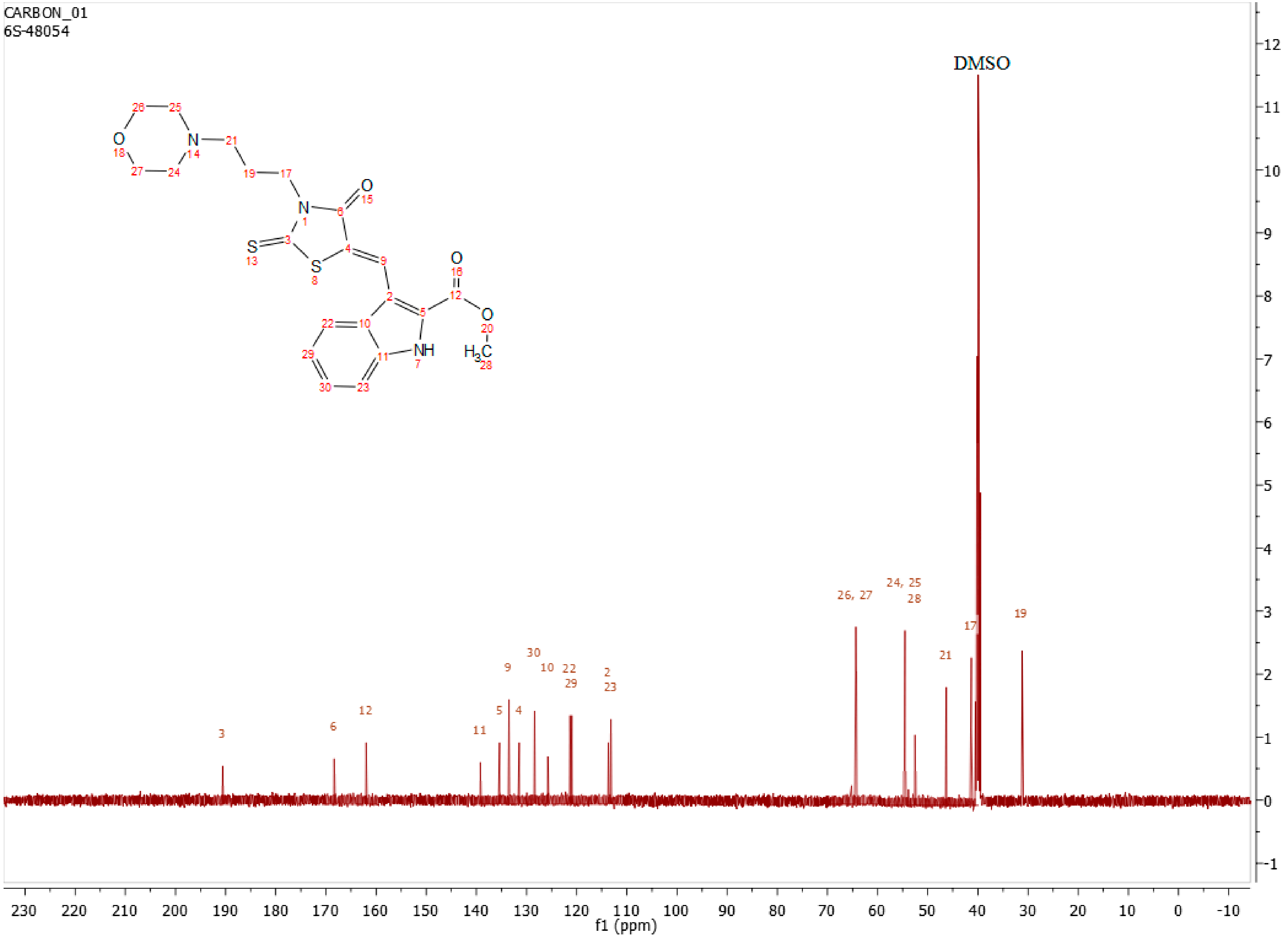

CARBON_01
6S-48054
DMSO
3
6
12
11
5
9
4
30
10
22
29
2
23
26, 27
24, 25
28
21
17
19
230
220
210
200
190
180
170
160
150
140
130
120
110
100
90
80
70
60
50
40
30
20
10
0
-10
f1 (ppm)

Compound 6

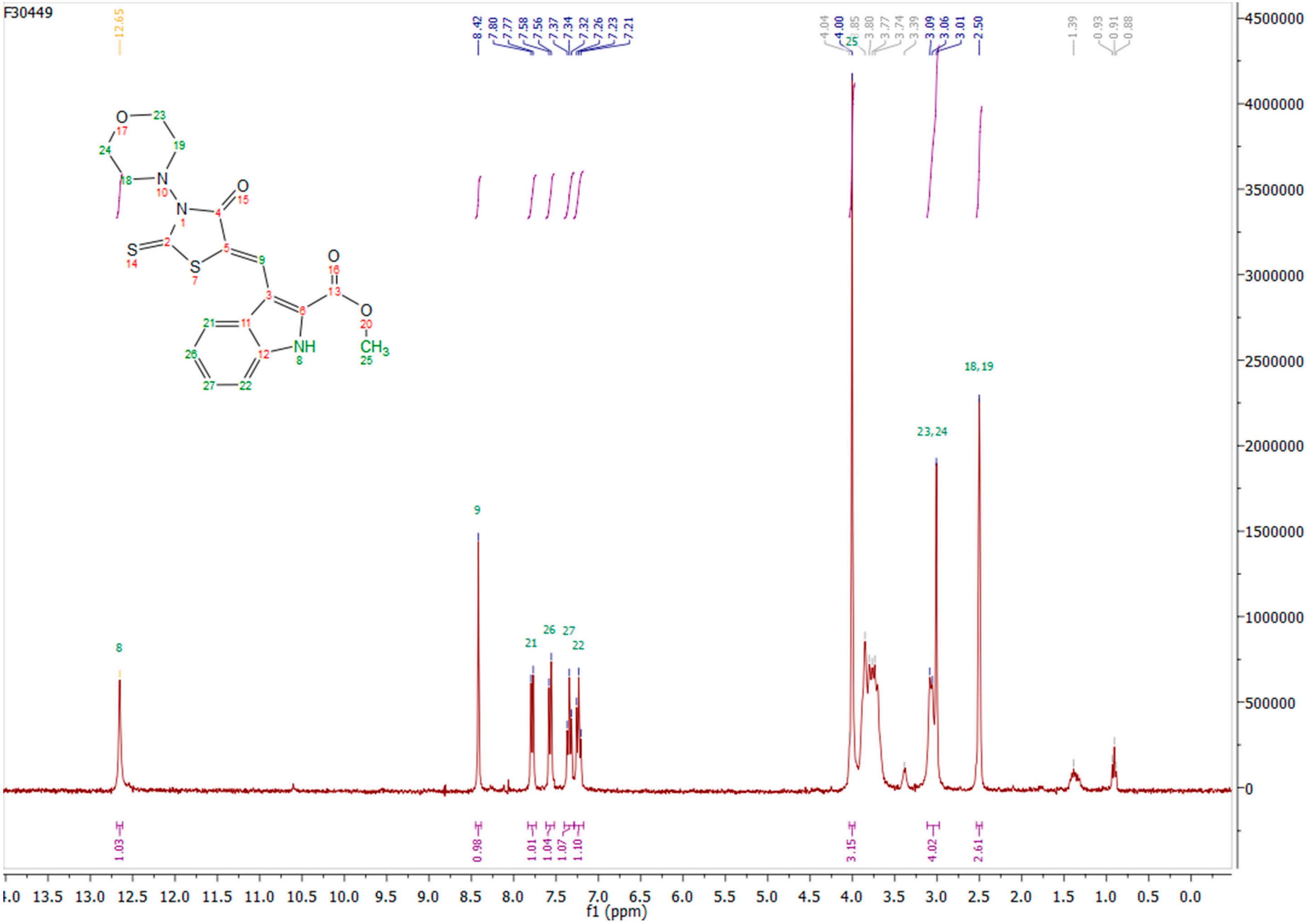

F30449
12.65
8.42
7.80
7.77
7.58
7.56
7.37
7.34
7.32
7.26
7.23
7.21
4.04
4.00
3.85
3.80
3.77
3.74
3.39
3.09
3.06
3.01
2.50
1.39
0.93
0.91
0.88
25
23,24
18,19
9
8
21
26
27
22
NH
CH3
1.03
0.98
1.01
1.04
1.07
1.10
3.15
4.02
2.61
f1 (ppm)

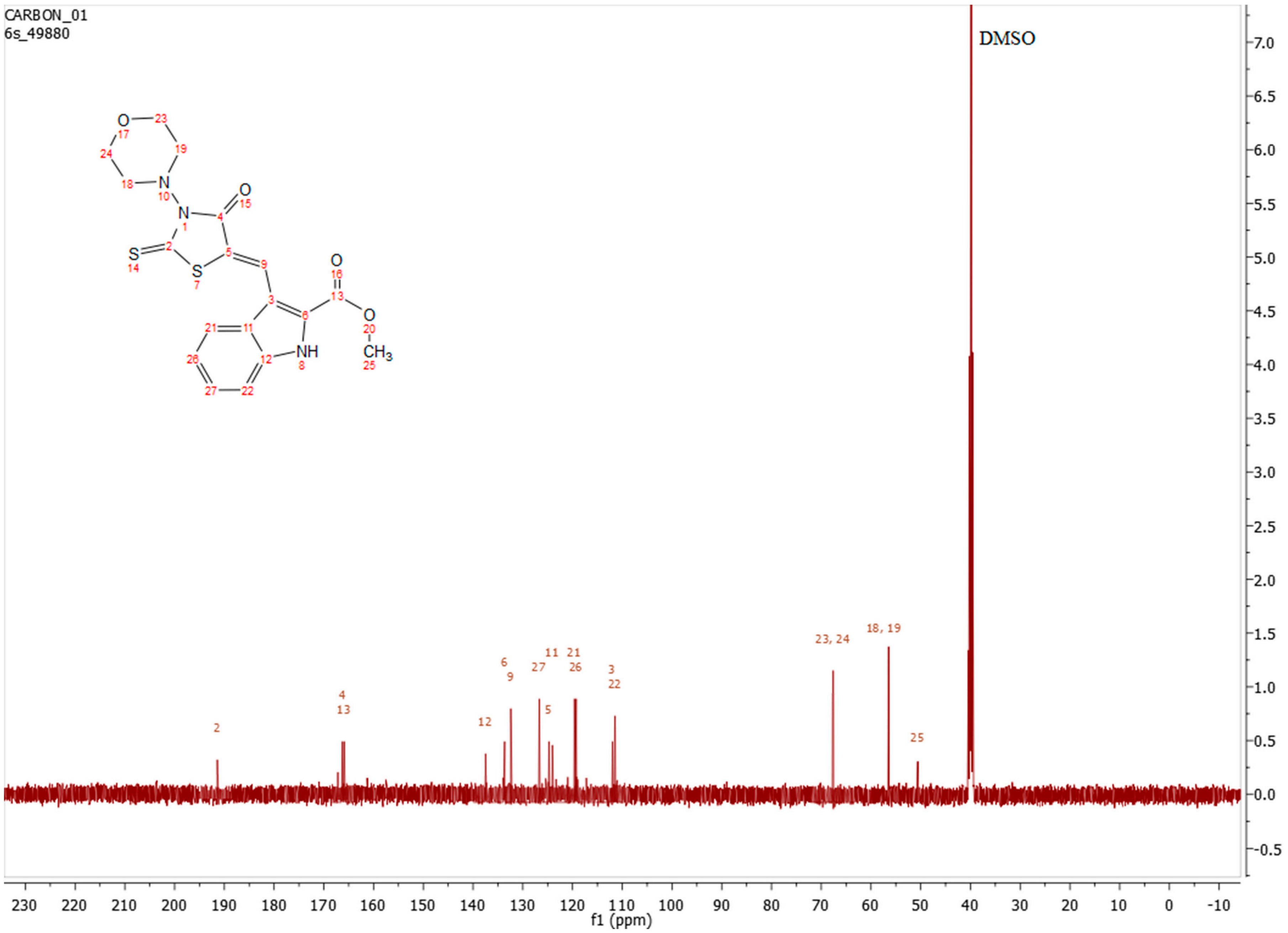
CARBON_01
6s_49880
DMSO
2
4
13
12
6
9
27
5
11
21
26
3
22
23, 24
18, 19
25
f1 (ppm)

Compound 7

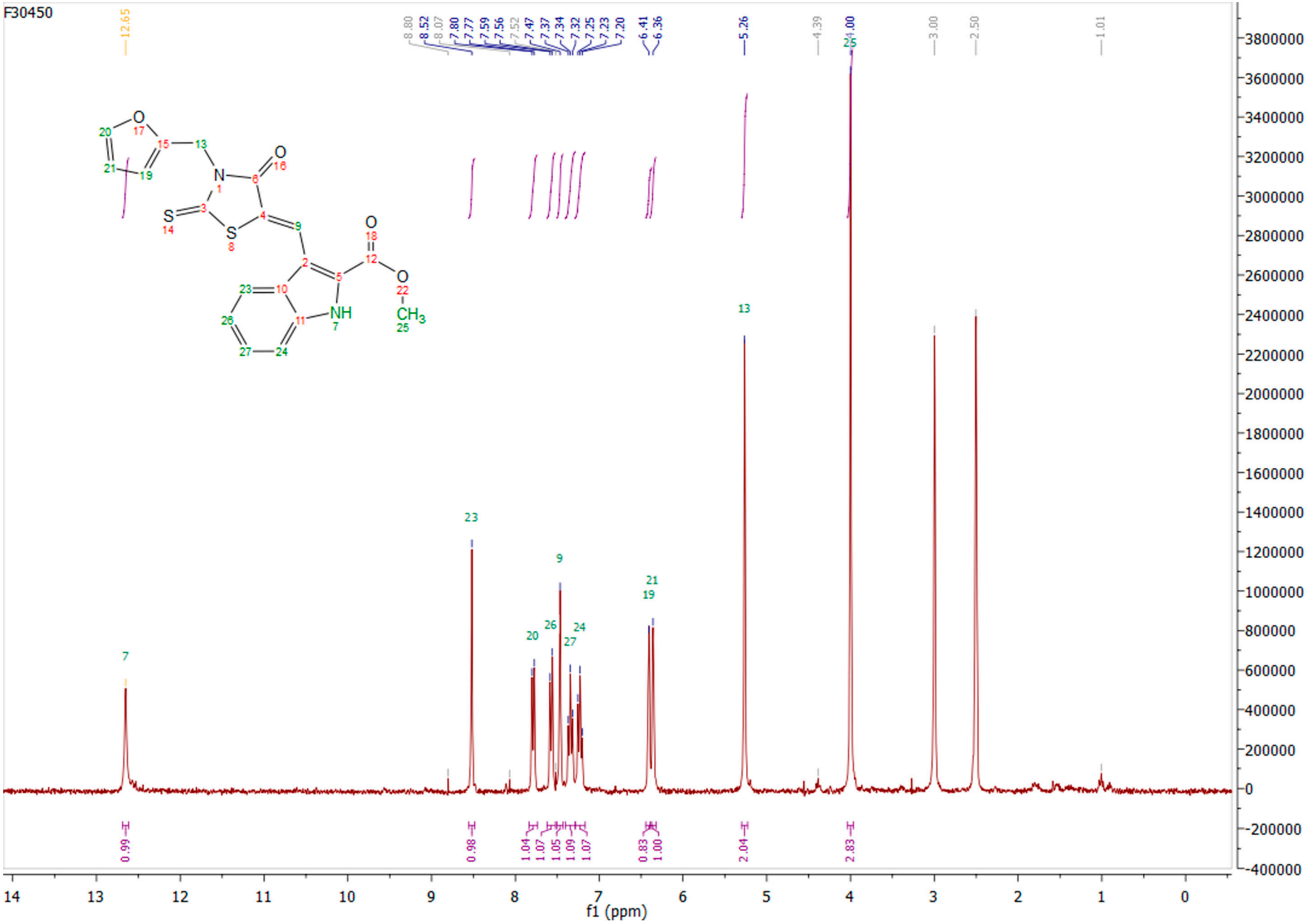

F30450
12.65
8.80 8.52 8.07 7.80 7.77 7.59 7.56 7.52 7.47 7.37 7.34 7.32 7.25 7.23 7.20
6.41 6.36
5.26
4.39
4.00
3.00
2.50
1.01
0.99 0.98 1.04 1.07 1.05 1.09 1.07 0.83 1.00 2.04 2.83
f1 (ppm)

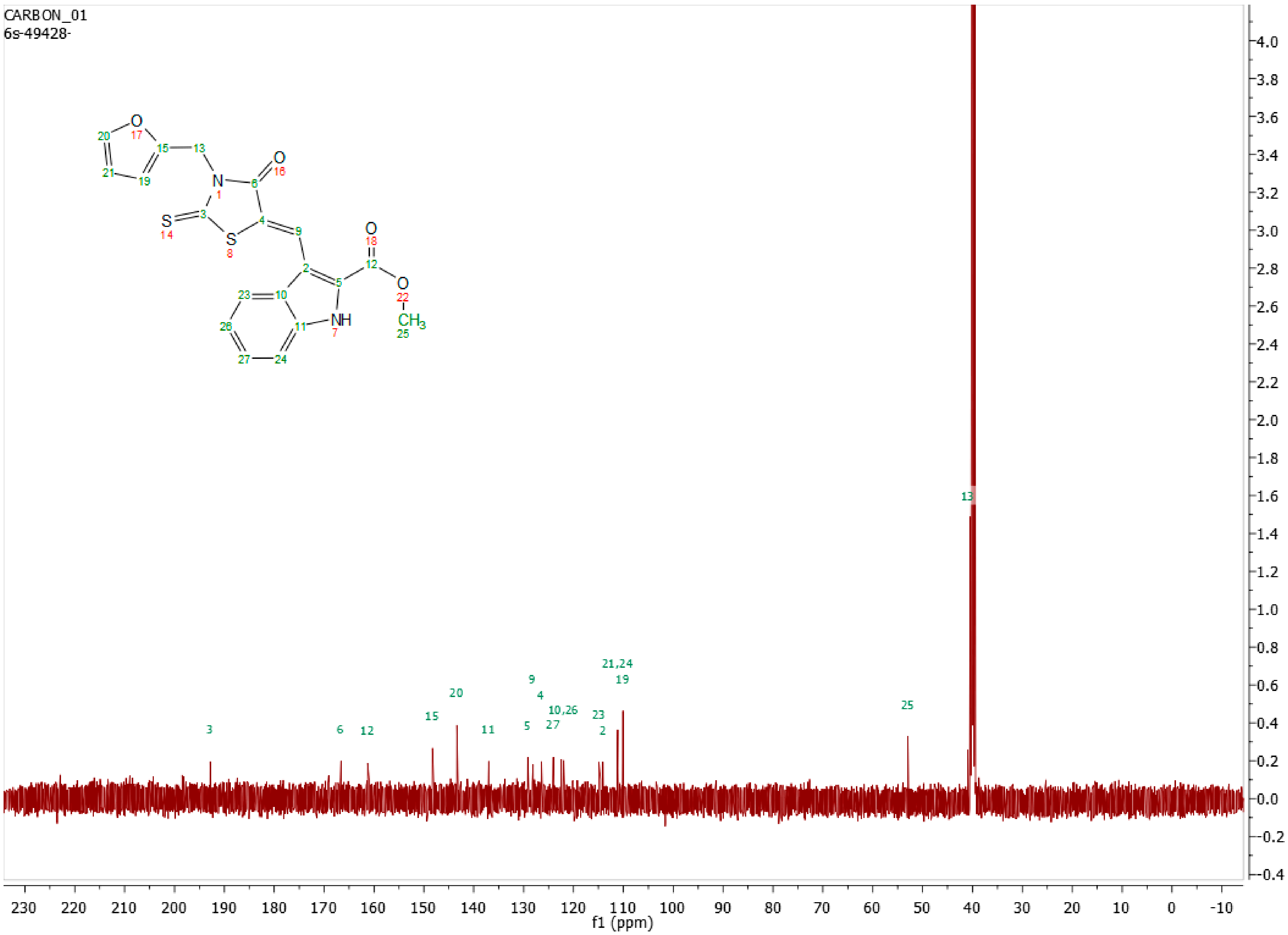

CARBON_01
6s-49428-
f1 (ppm)

Compound 8

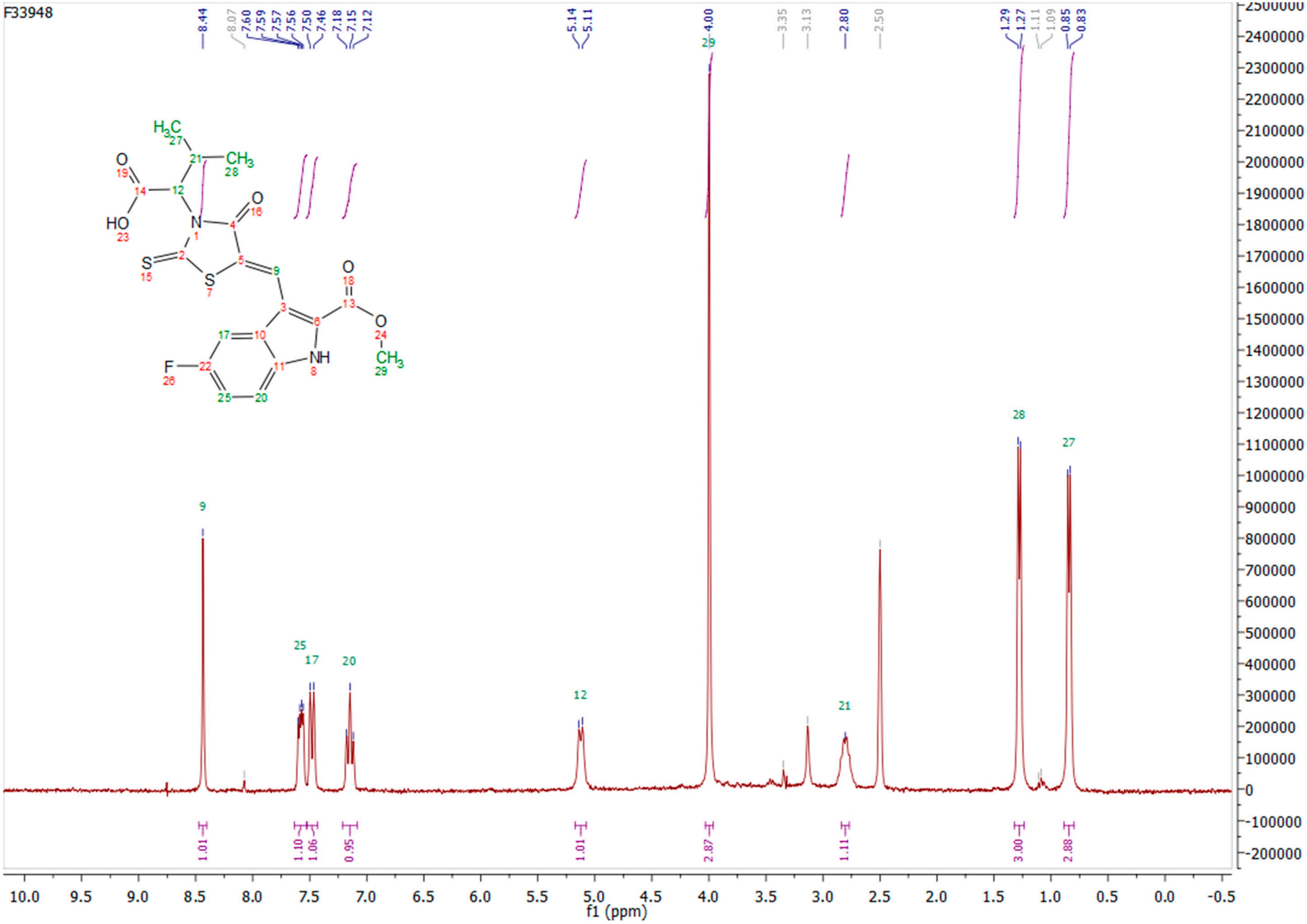

F33948
8.44
8.07
7.60
7.59
7.57
7.56
7.50
7.46
7.18
7.15
7.12
5.14
5.11
4.00
3.35
3.13
2.80
2.50
1.29
1.27
1.11
1.09
0.85
0.83
1.01
1.10
1.06
0.95
1.01
2.87
1.11
3.00
2.88
f1 (ppm)

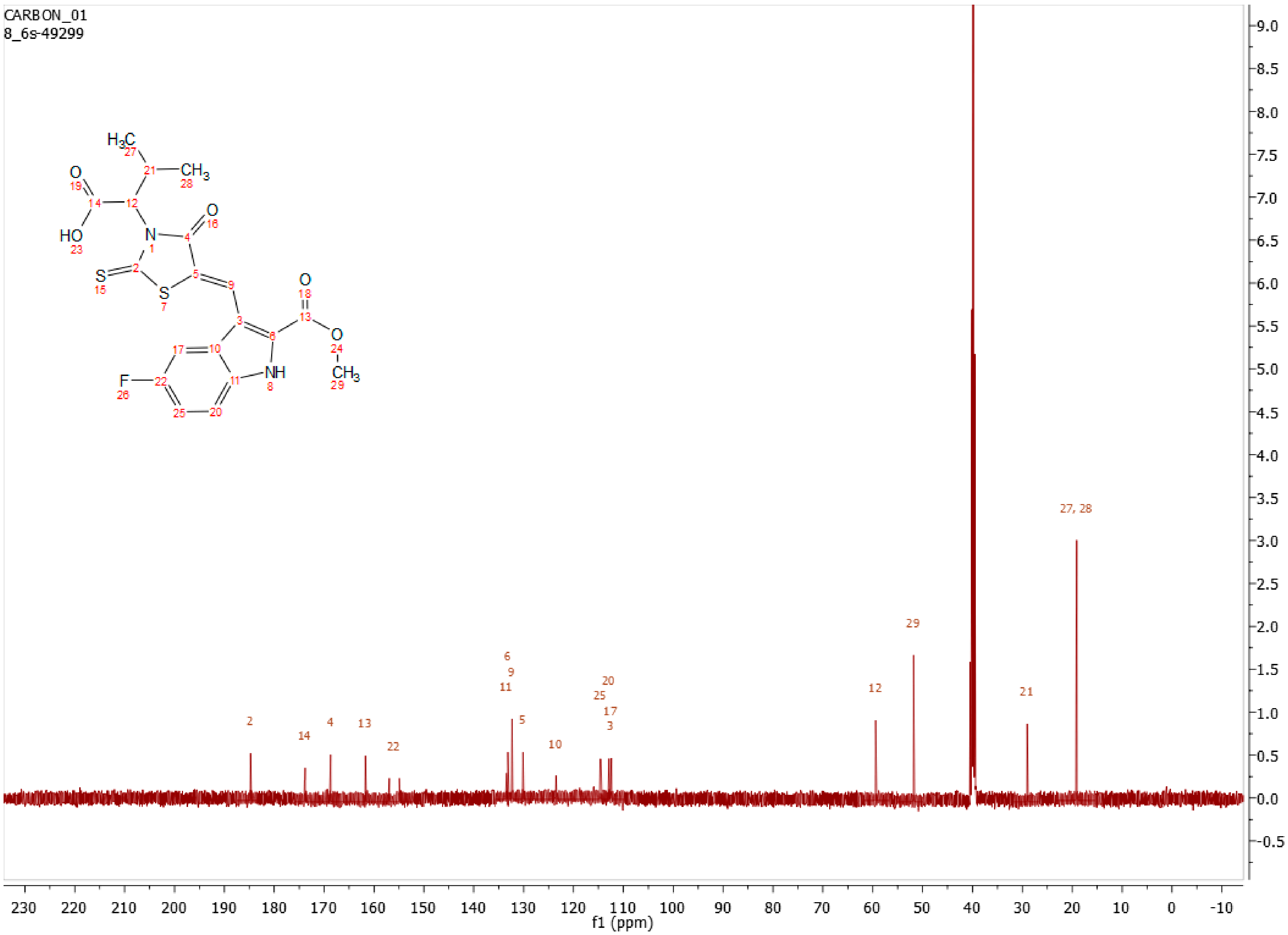

CARBON_01
8_6s-49299
H3C
CH3
O
HO
N
O
S
S
O
O
F
NH
CH3
2
14
4
13
22
6
9
11
5
10
20
25
17
3
12
29
21
27, 28
f1 (ppm)

Compound 9

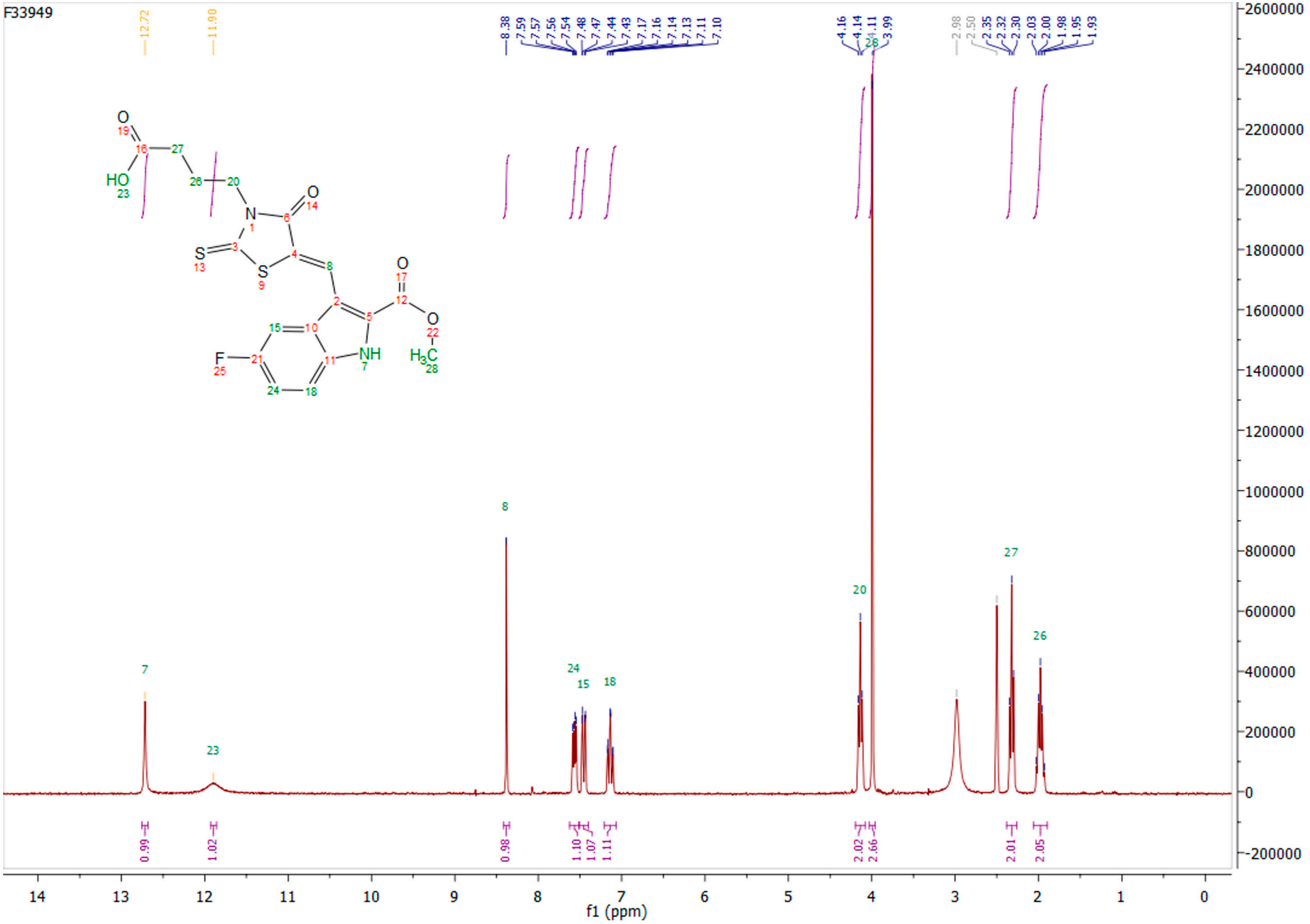
F33949
12.72
11.90
8.38
7.59
7.57
7.56
7.54
7.48
7.47
7.44
7.43
7.17
7.16
7.14
7.13
7.11
7.10
4.16
4.14
4.11
3.99
2.98
2.50
2.35
2.32
2.30
2.03
2.00
1.98
1.95
1.93
0.99
1.02
0.98
1.10
1.07
1.11
2.02
2.66
2.01
2.05
f1 (ppm)

CARBON_01

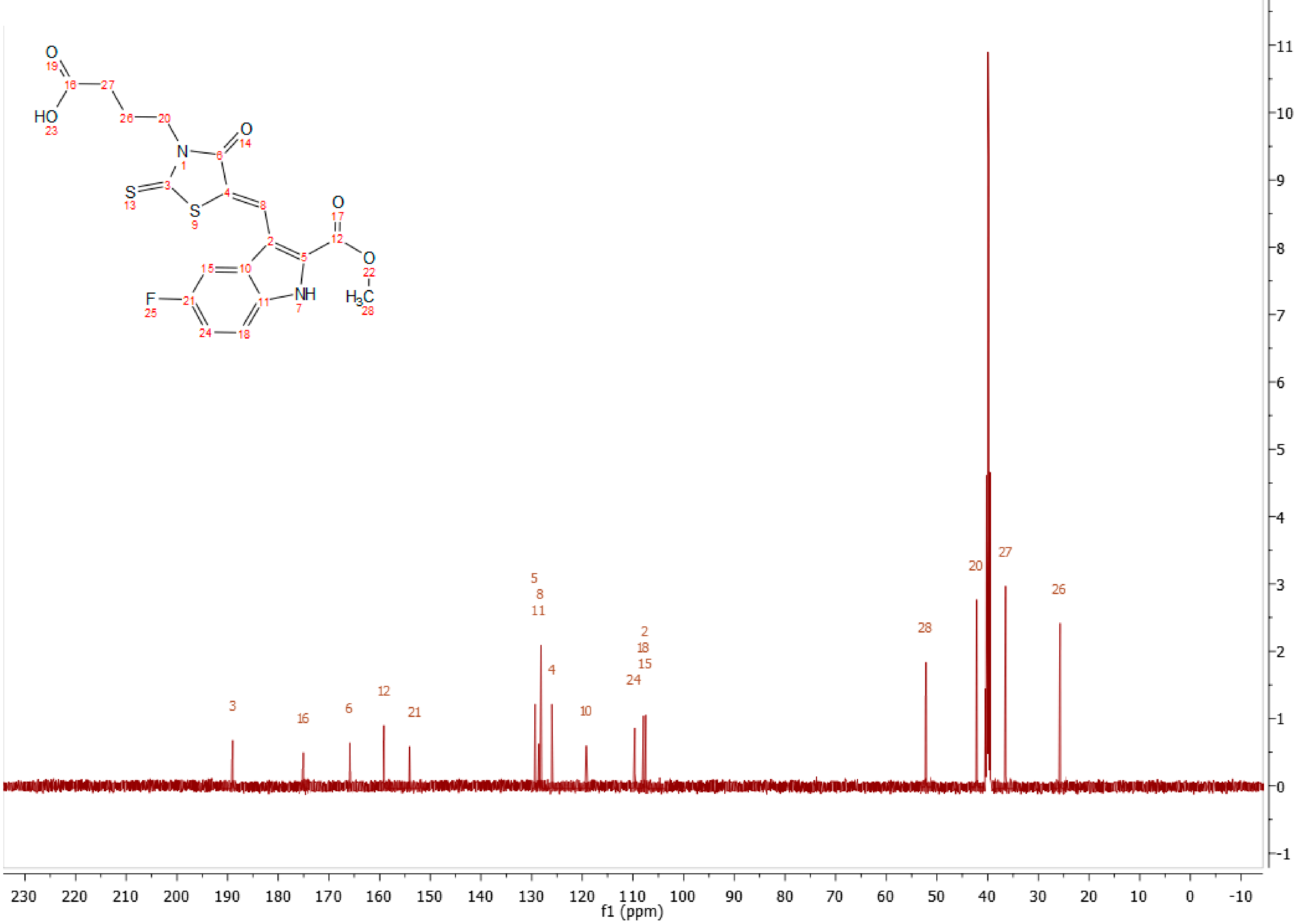

O
19
16
27
HO
23
26
20
N
1
6
O
14
S
13
3
S
9
4
8
O
17
12
2
5
O
22
15
10
F
25
21
11
NH
7
H3C
28
24
18
3
16
6
12
21
5
8
11
4
10
2
18
15
24
28
20
27
26
230
220
210
200
190
180
170
160
150
140
130
120
110
100
90
80
70
60
50
40
30
20
10
0
-10
f1 (ppm)

Compound 10

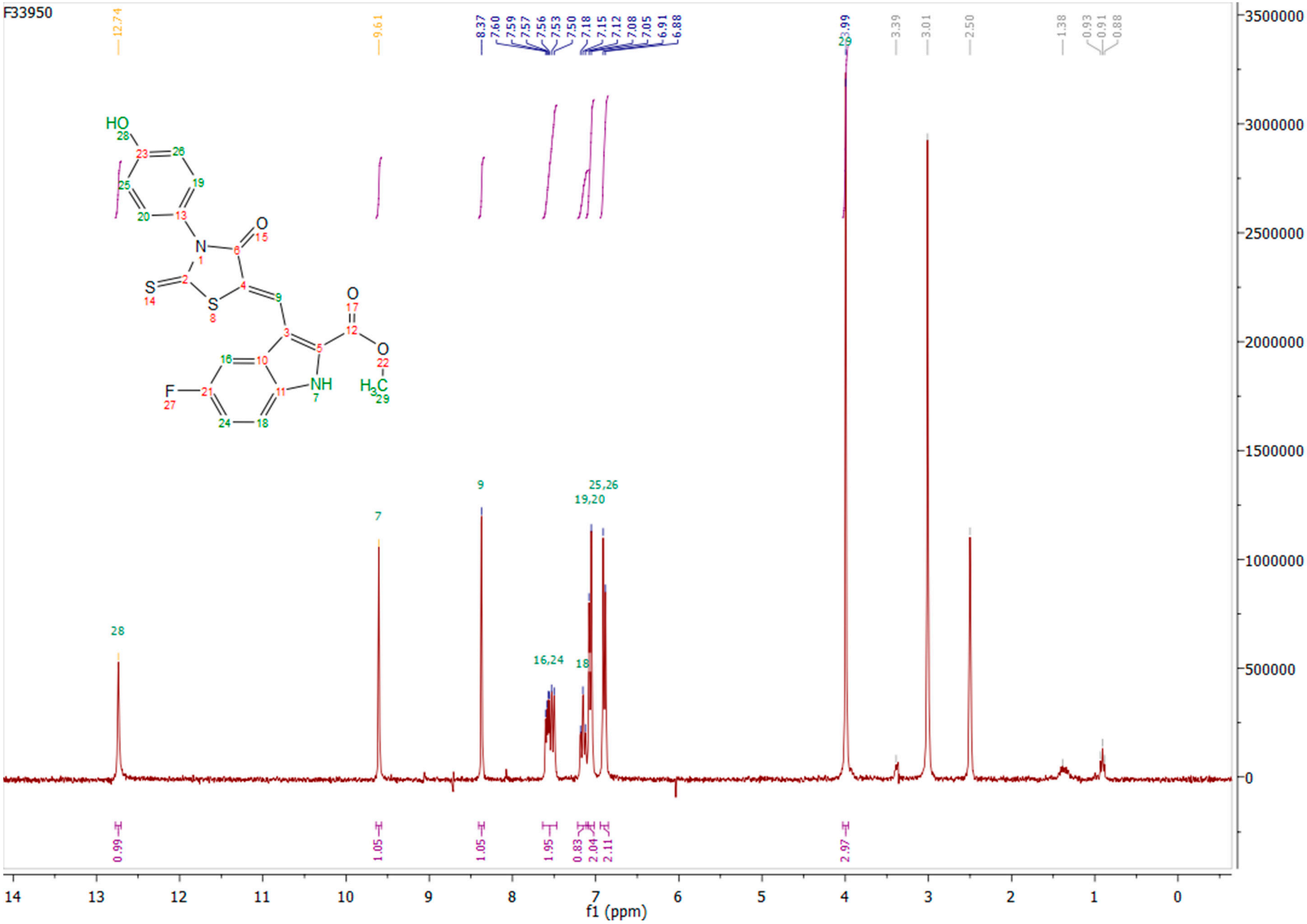

F33950
12.74
9.61
8.37
7.60
7.59
7.57
7.56
7.53
7.50
7.18
7.15
7.12
7.08
7.05
6.91
6.88
3.99
3.39
3.01
2.50
1.38
0.93
0.91
0.88
HO
NH
F
S
O
H_3C
28
7
9
16,24
18
19,20
25,26
29
0.99
1.05
1.05
1.95
0.83
2.04
2.11
2.97
f1 (ppm)
3500000
3000000
2500000
2000000
1500000
1000000
500000
0

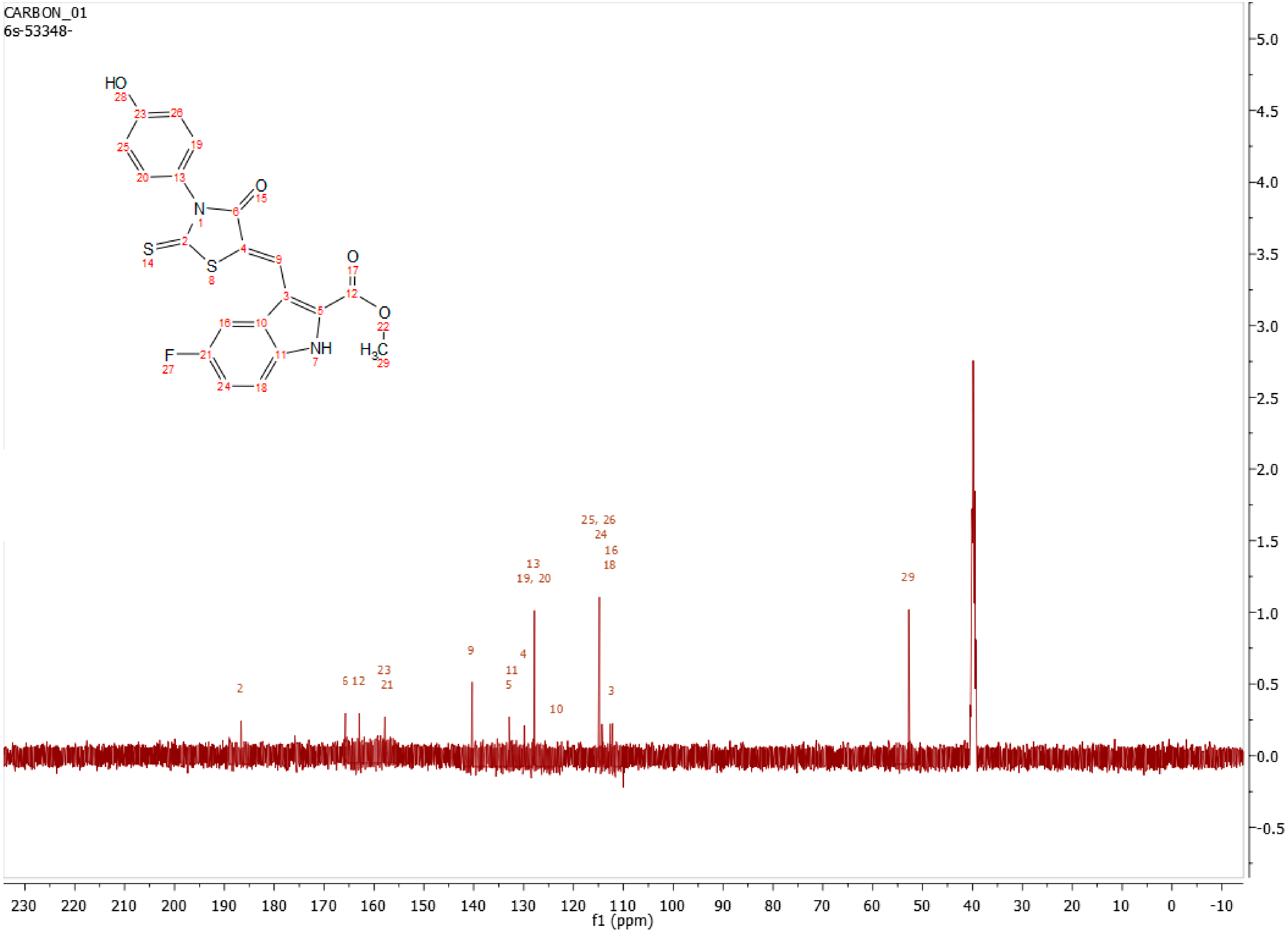
CARBON_01
6s-53348-
HO
N
O
S
S
O
O
F
NH
H3C
2
6 12
23
21
9
11
5
4
13
19, 20
10
25, 26
24
16
18
3
29
f1 (ppm)

Compound 11

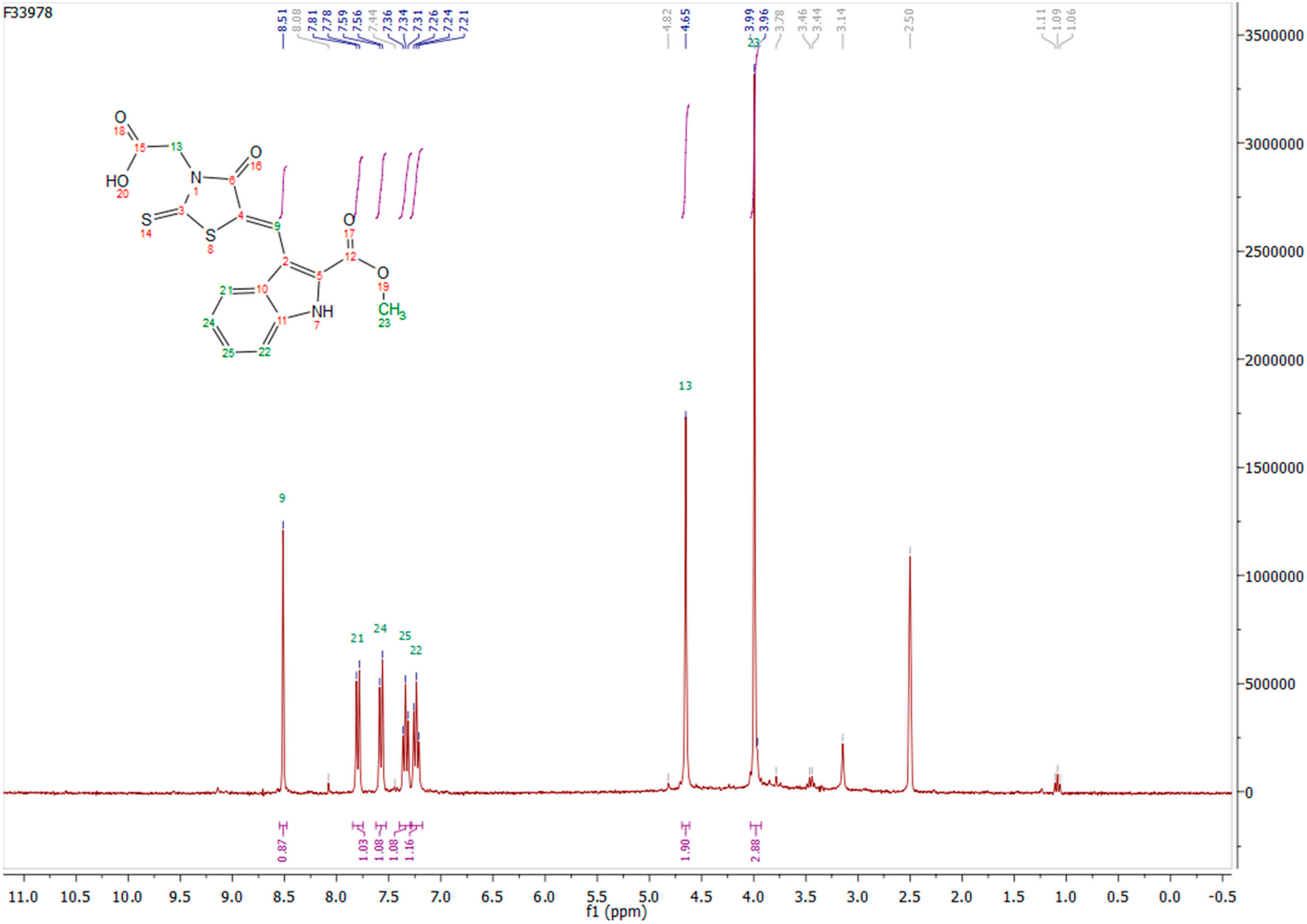
F33978
8.51
8.08
7.81
7.78
7.59
7.56
7.44
7.36
7.34
7.31
7.26
7.24
7.21
4.82
4.65
3.99
3.96
3.78
3.46
3.44
3.14
2.50
1.11
1.09
1.06
9
21
24
25
22
13
23
0.87
1.03
1.08
1.08
1.16
1.90
2.88
f1 (ppm)

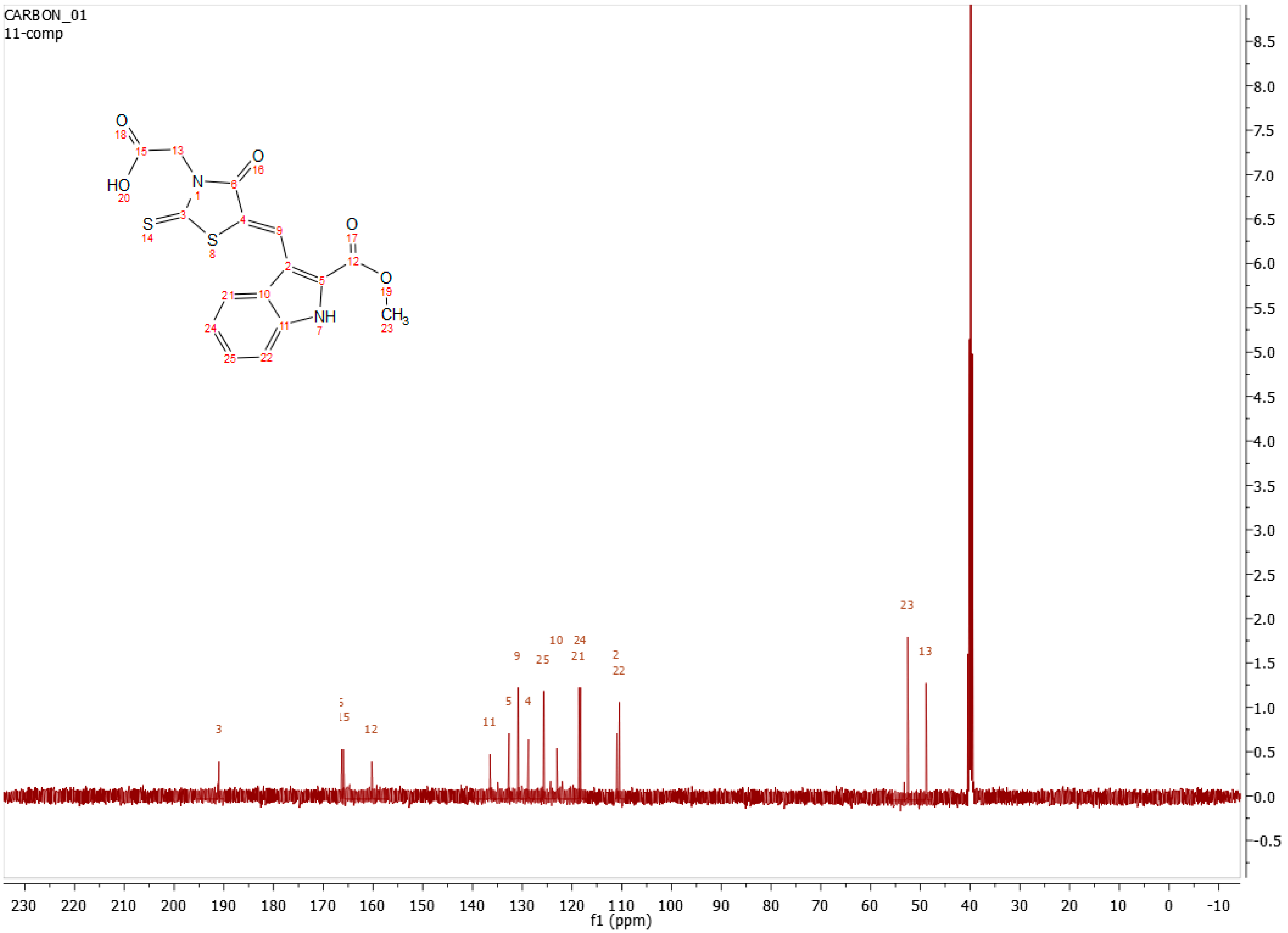

CARBON_01
11-comp
f1 (ppm)

Compound 12

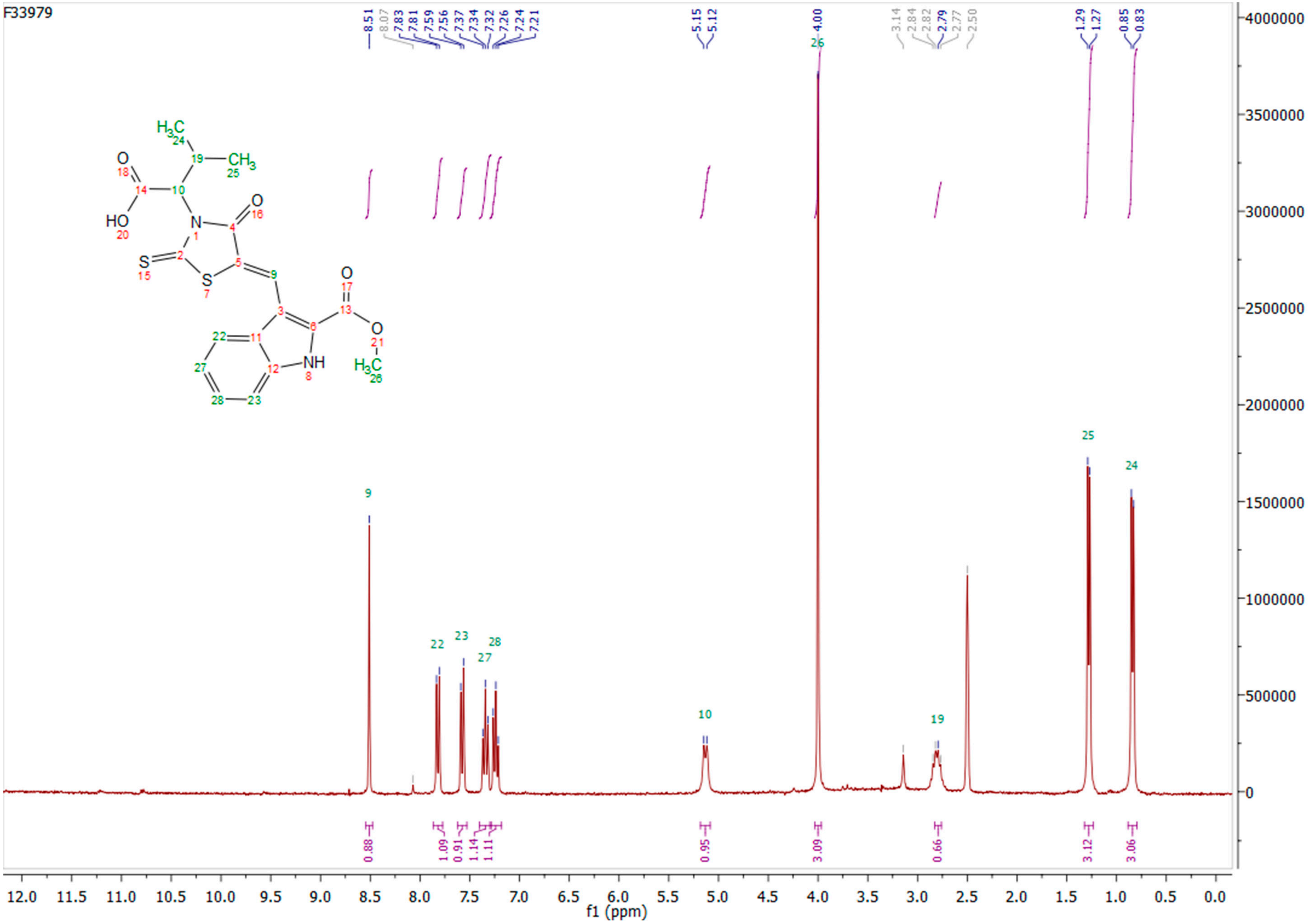

F33979
8.51
8.07
7.83
7.81
7.59
7.56
7.37
7.34
7.32
7.26
7.24
7.21
5.15
5.12
4.00
3.14
2.84
2.82
2.79
2.77
2.50
1.29
1.27
0.85
0.83
0.88
1.09
0.91
1.14
1.11
0.95
3.09
0.66
3.12
3.06
f1 (ppm)

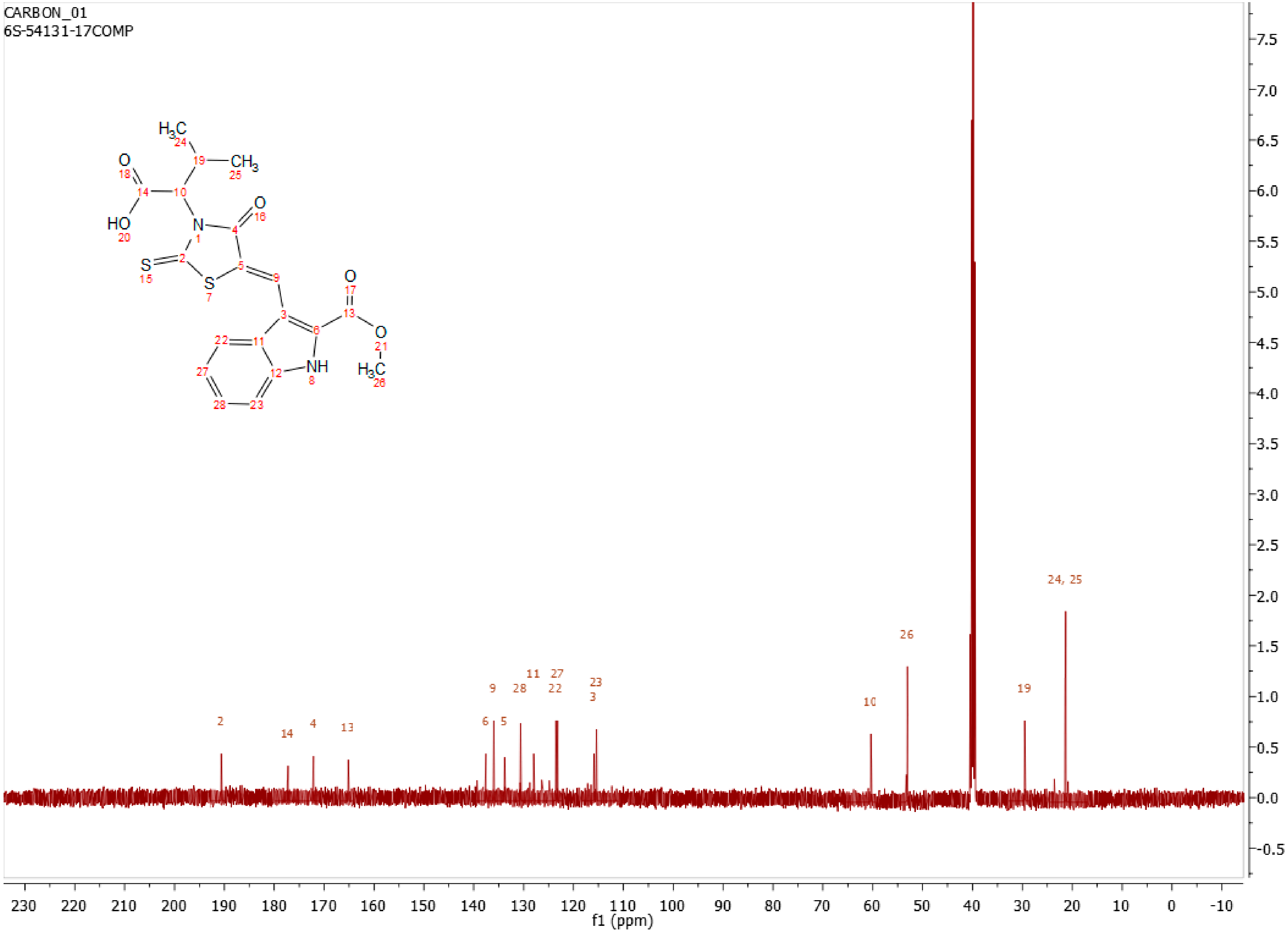

CARBON_01
6S-54131-17COMP
2
14
4
13
6
9
5
28
11
27
22
23
3
10
26
19
24, 25
f1 (ppm)

Compound 13

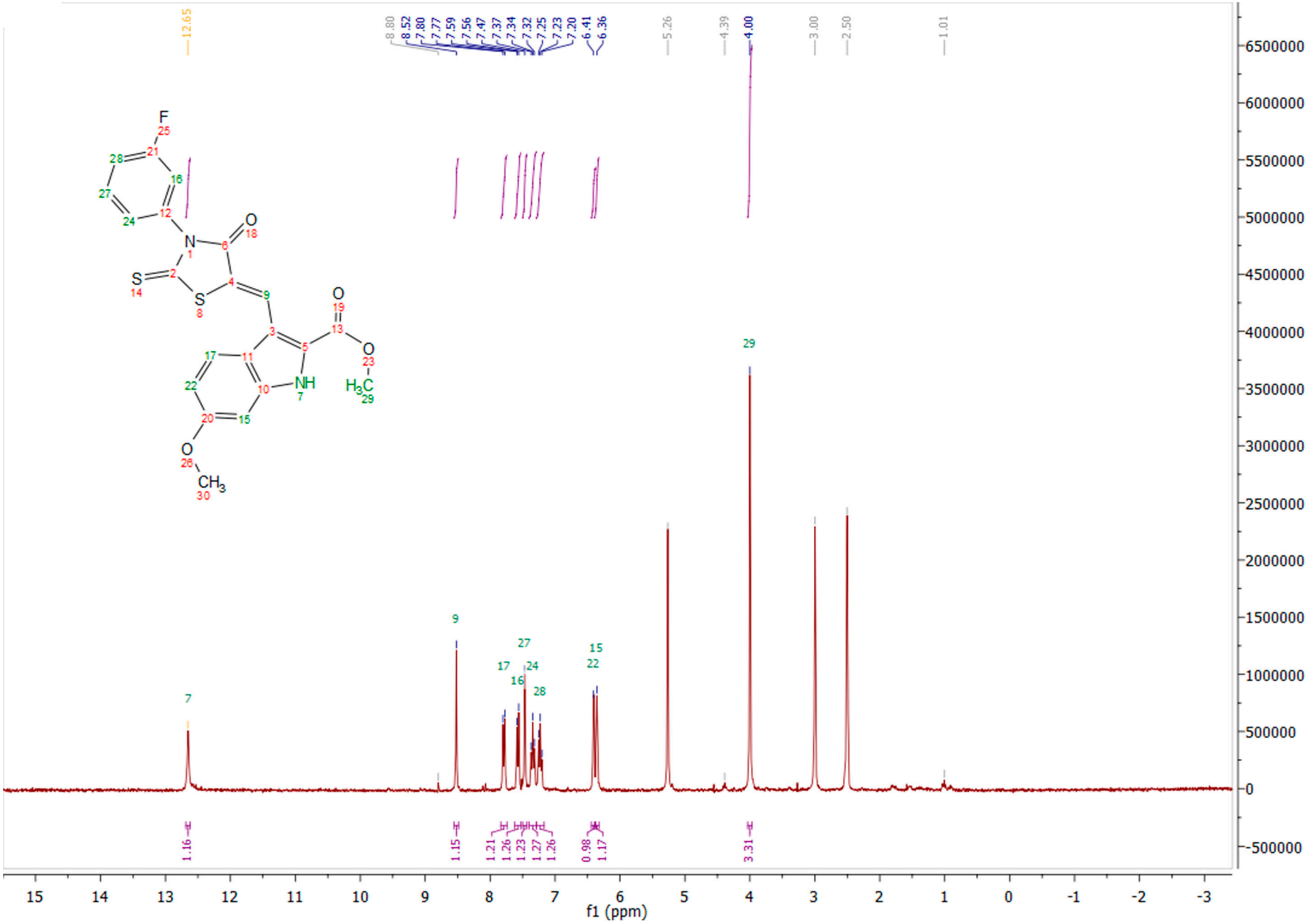

12.65
8.80
8.52
7.80
7.77
7.59
7.56
7.47
7.37
7.34
7.32
7.25
7.23
7.20
6.41
6.36
5.26
4.39
4.00
3.00
2.50
1.01
F
25
21
28
16
27
12
24
O
18
N
1
6
S
14
2
4
9
S
8
O
19
13
3
5
O
23
17
11
22
10
NH
7
H3C
29
20
15
O
26
CH3
30
29
9
27
17
24
16
28
15
22
7
1.16
1.15
1.21
1.26
1.23
1.27
1.26
0.98
1.17
3.31
6500000
6000000
5500000
5000000
4500000
4000000
3500000
3000000
2500000
2000000
1500000
1000000
500000
0
-500000
15
14
13
12
11
10
9
8
7
6
5
4
3
2
1
0
-1
-2
-3
f1 (ppm)

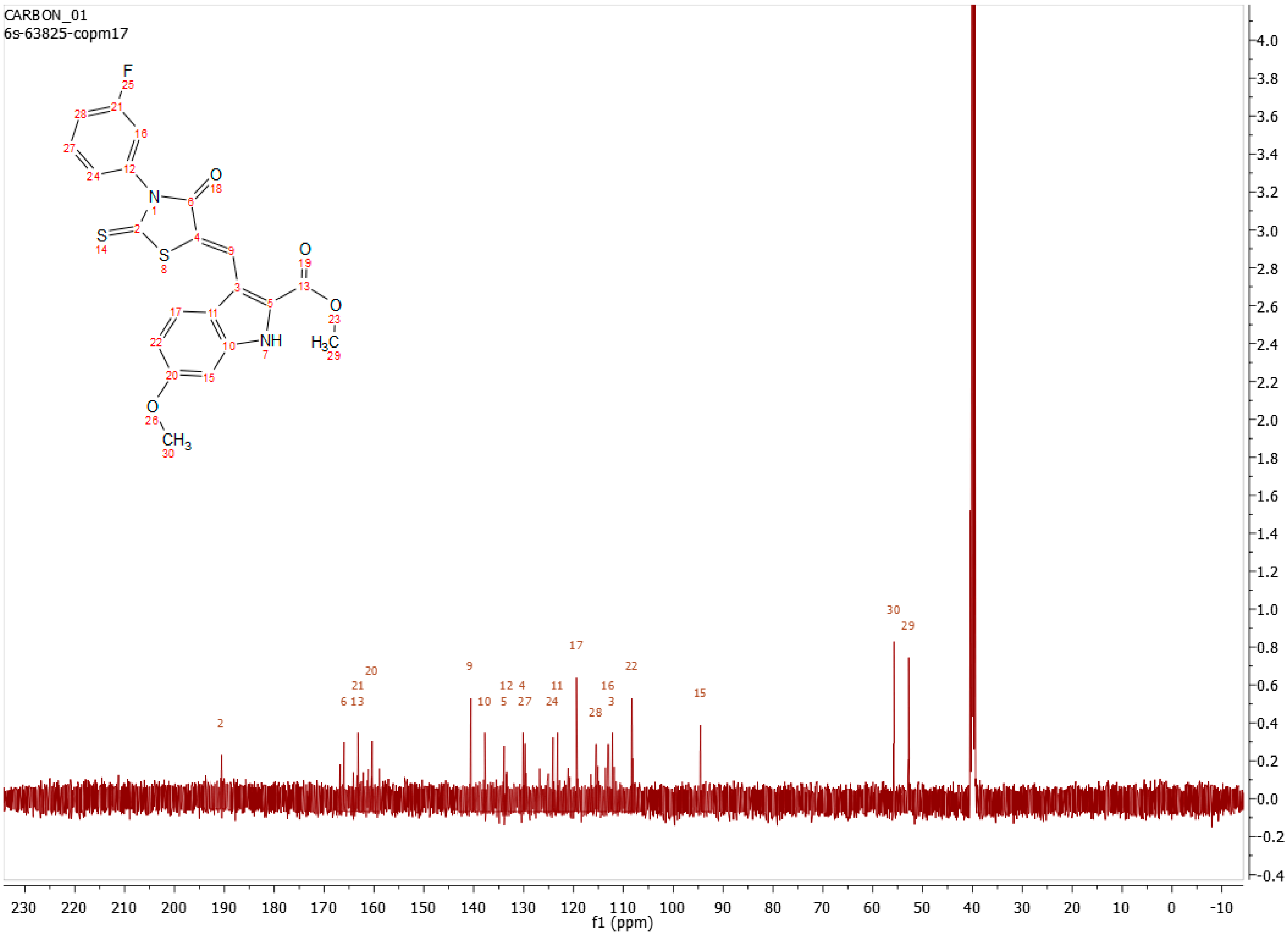

CARBON_01
6s-63825-copm17
f1 (ppm)

Compound 14

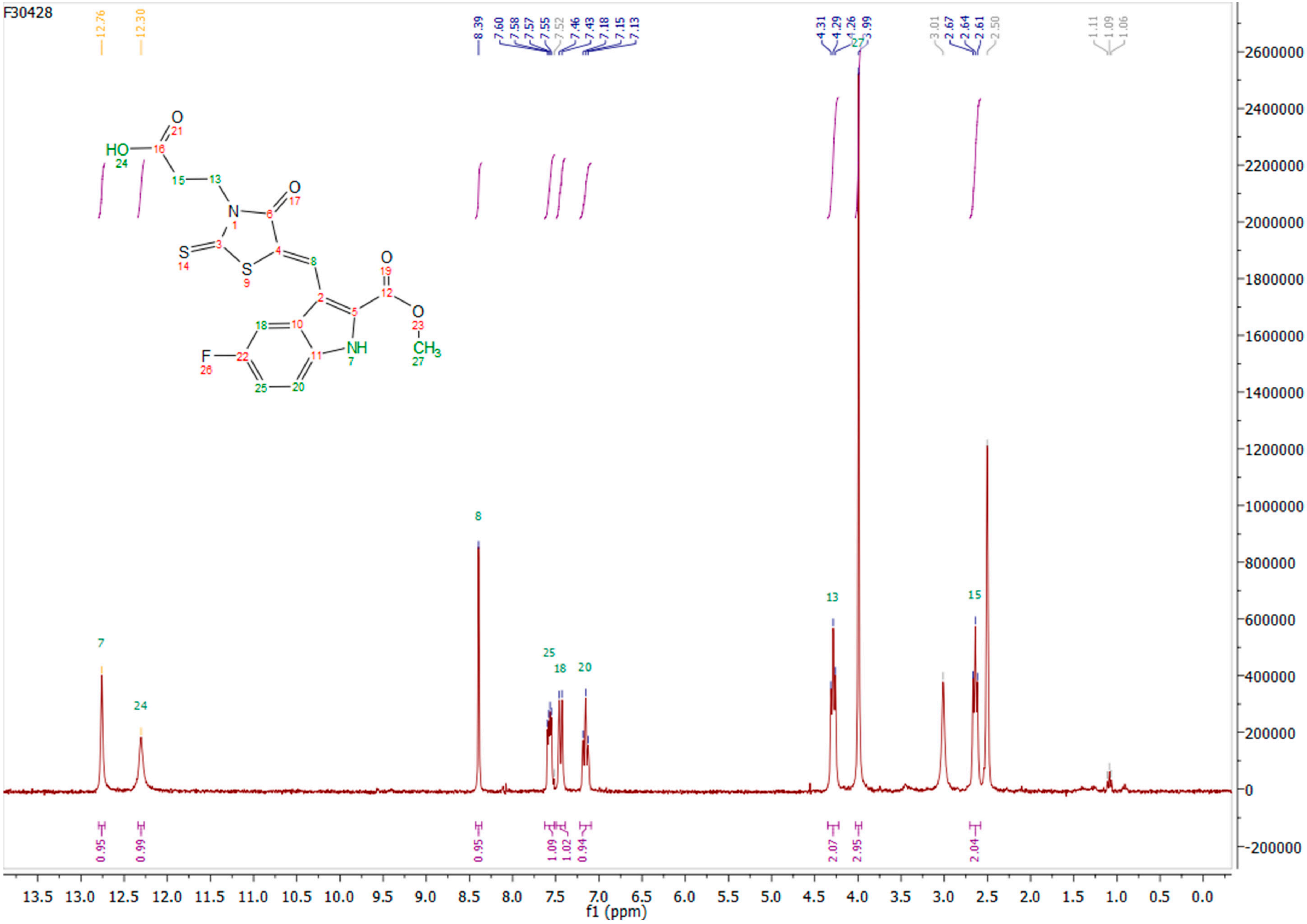

F30428
12.76
12.30
8.39
7.60
7.58
7.57
7.55
7.52
7.46
7.43
7.18
7.15
7.13
4.31
4.29
4.26
3.99
3.01
2.67
2.64
2.61
2.50
1.11
1.09
1.06
0.95
0.99
0.95
1.09
1.02
0.94
2.07
2.95
2.04
f1 (ppm)

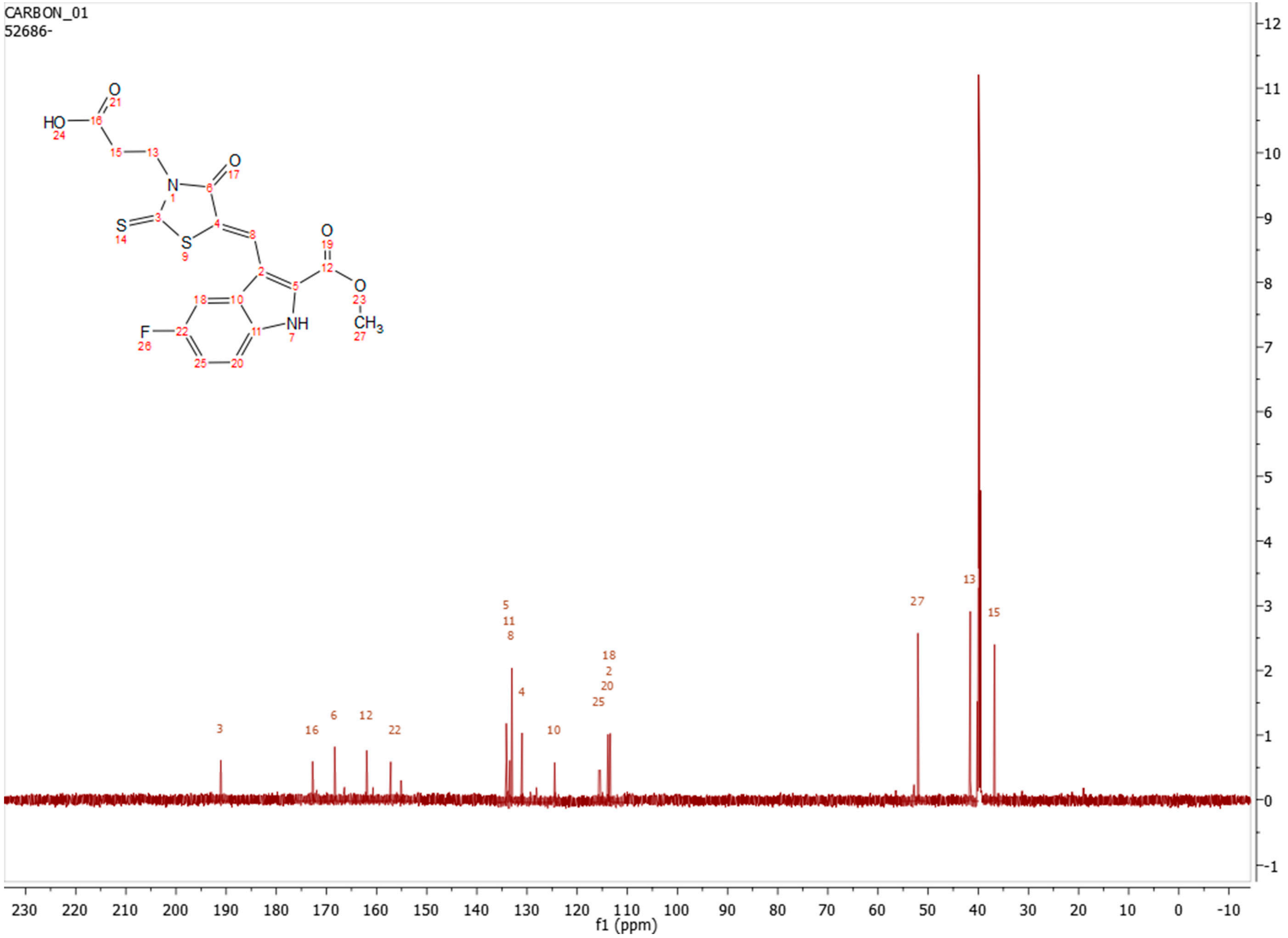

CARBON_01
52686-
HO
O
N
O
S
S
O
O
F
NH
CH3
3
16
6
12
22
5
11
8
4
10
18
2
20
25
27
13
15
f1 (ppm)

Compound 15

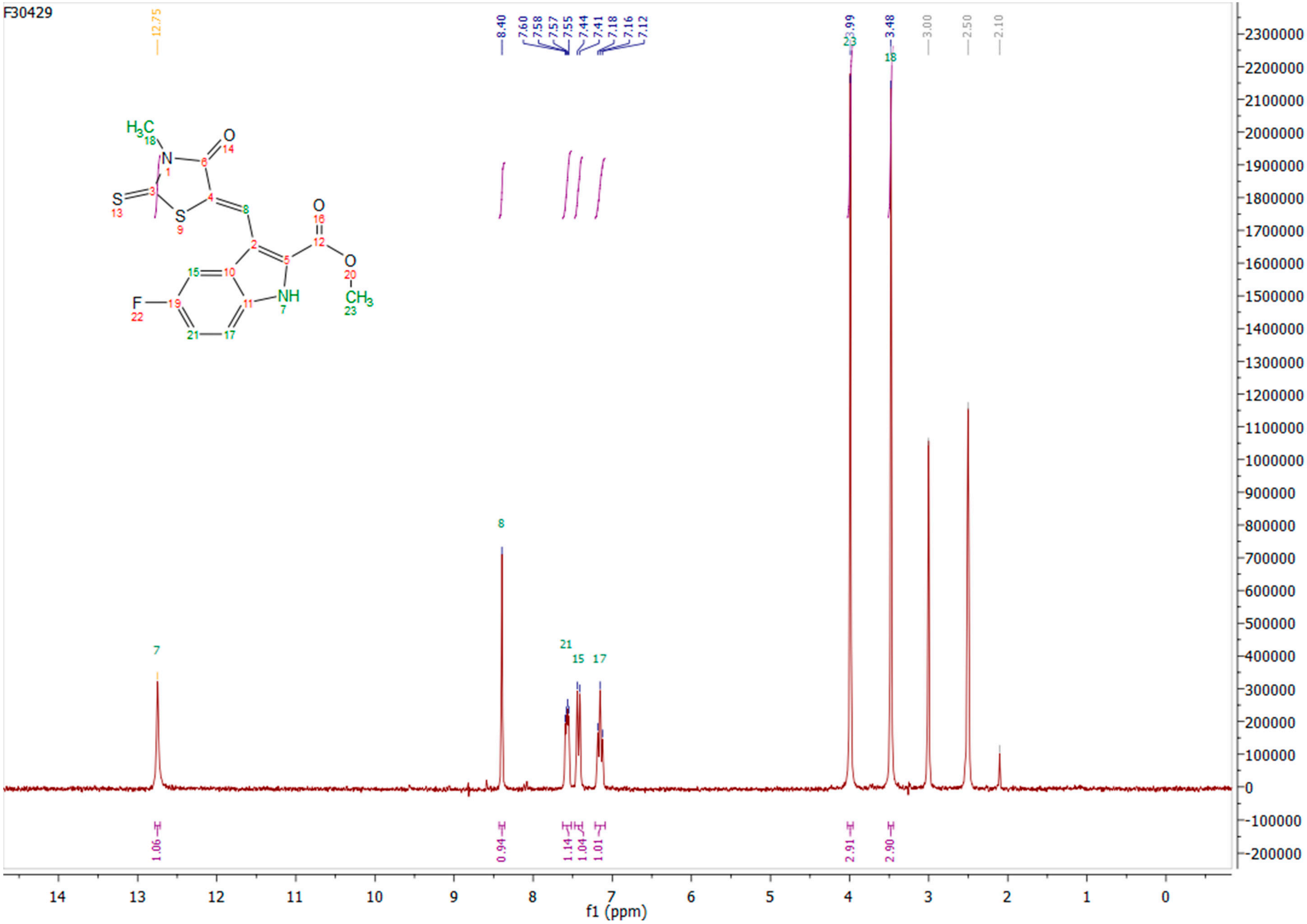

F30429
12.75
8.40
7.60
7.58
7.57
7.55
7.44
7.41
7.18
7.16
7.12
3.99
3.48
3.00
2.50
2.10
1.06
0.94
1.14
1.04
1.01
2.91
2.90
f1 (ppm)

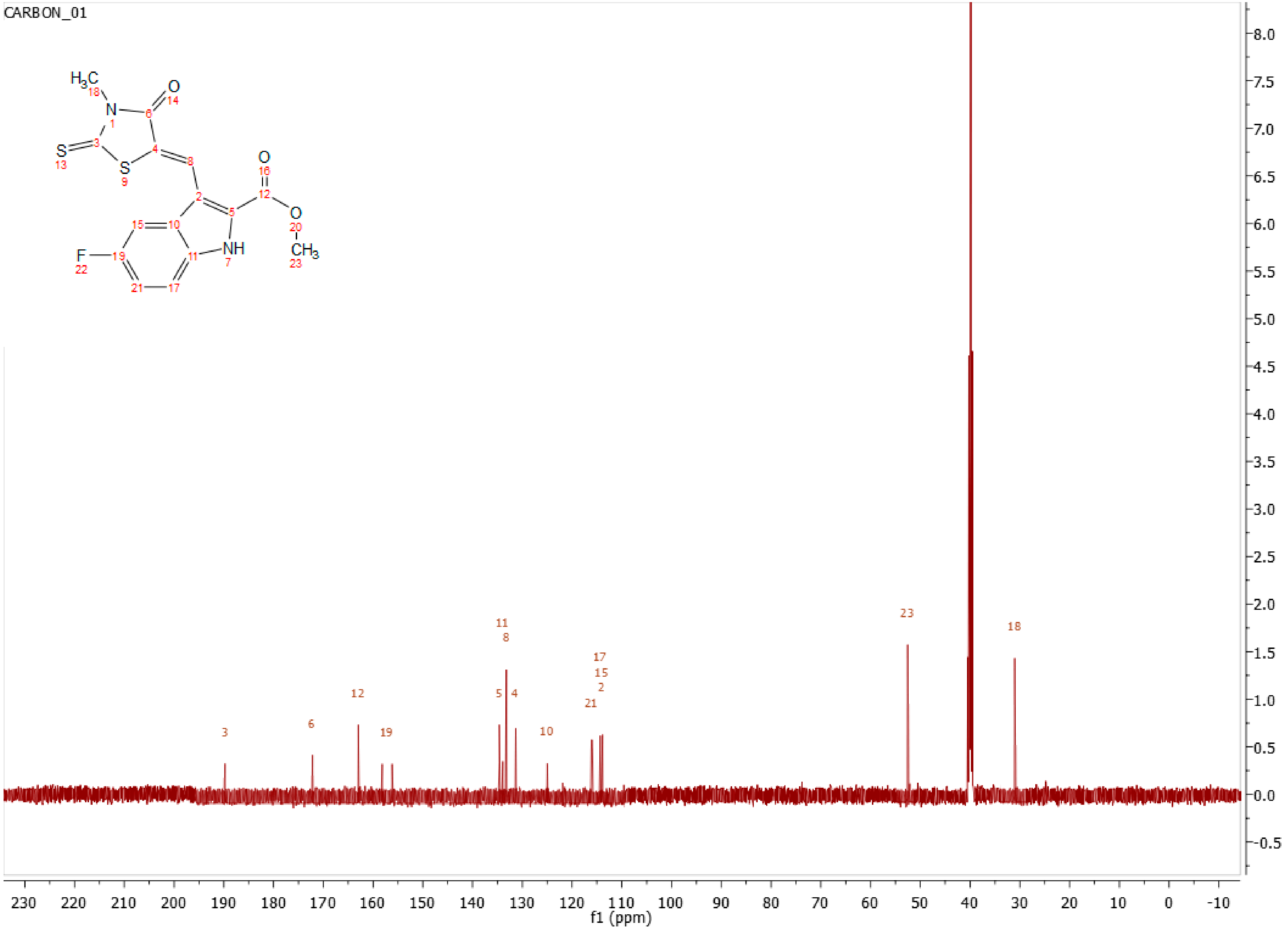
CARBON_01
H3C
18
N
1
O
14
6
S
13
3
4
S
9
8
O
16
12
2
5
O
20
15
10
F
22
19
11
NH
7
CH3
23
21
17
23
18
11
8
17
15
2
12
5
4
21
3
6
19
10
f1 (ppm)

Compound 16

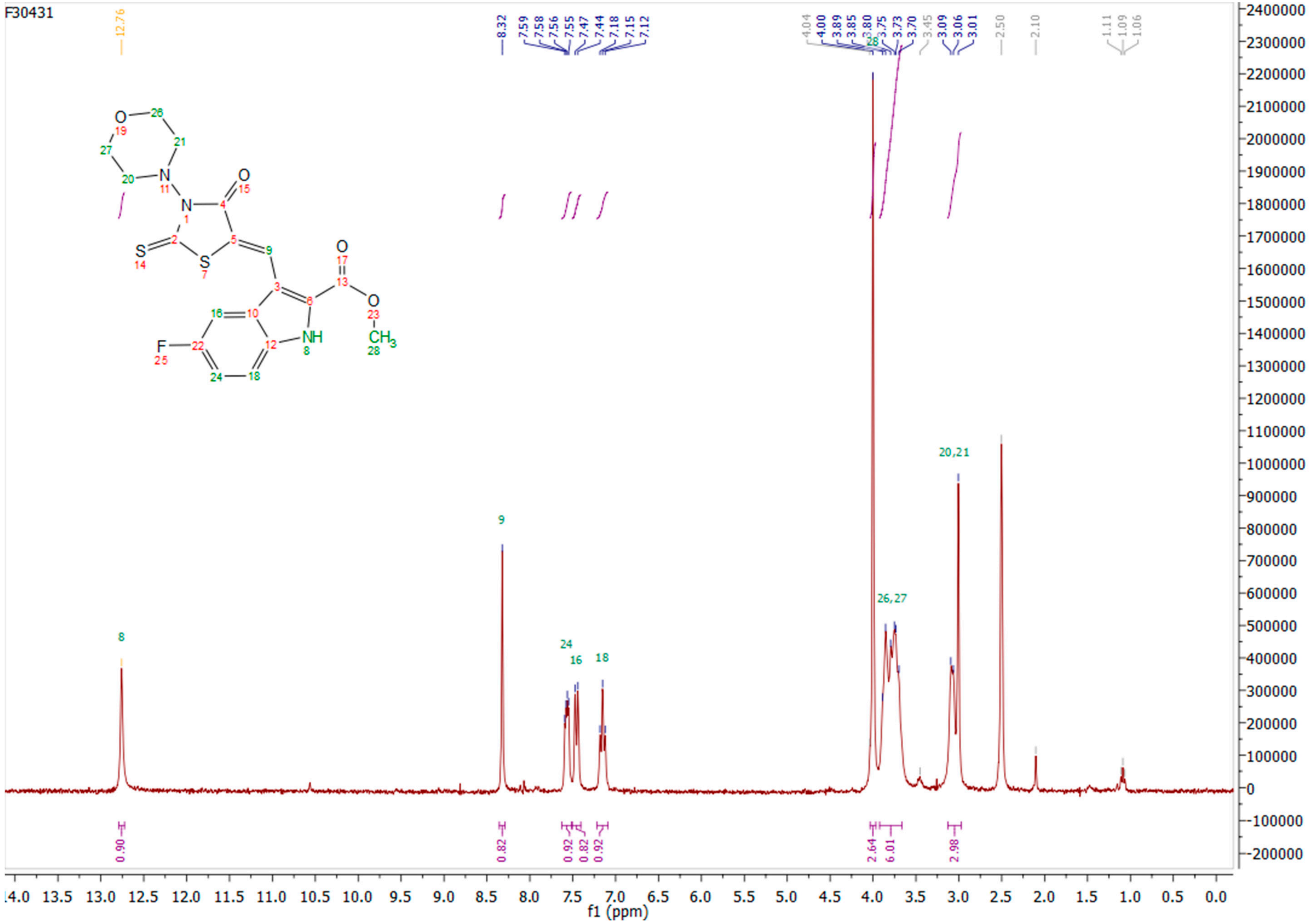
F30431
12.76
8.32
7.59
7.58
7.56
7.55
7.47
7.44
7.18
7.15
7.12
4.04
4.00
3.89
3.85
3.80
3.75
3.73
3.70
3.45
3.09
3.06
3.01
2.50
2.10
1.11
1.09
1.06
8
9
24
16
18
26,27
20,21
28
0.90
0.82
0.92
0.82
0.92
2.64
6.01
2.98
f1 (ppm)

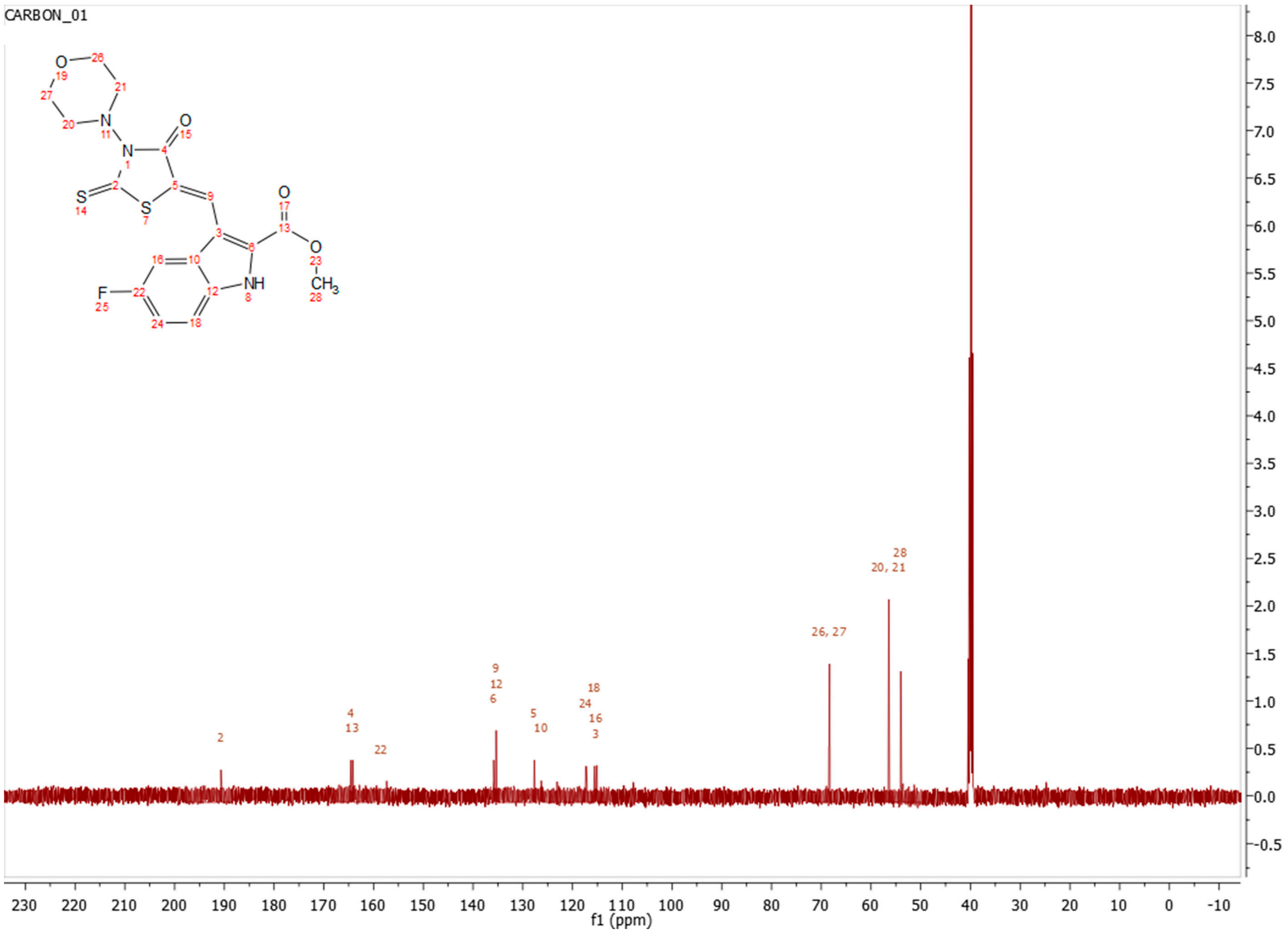

CARBON_01
f1 (ppm)

Compound 17

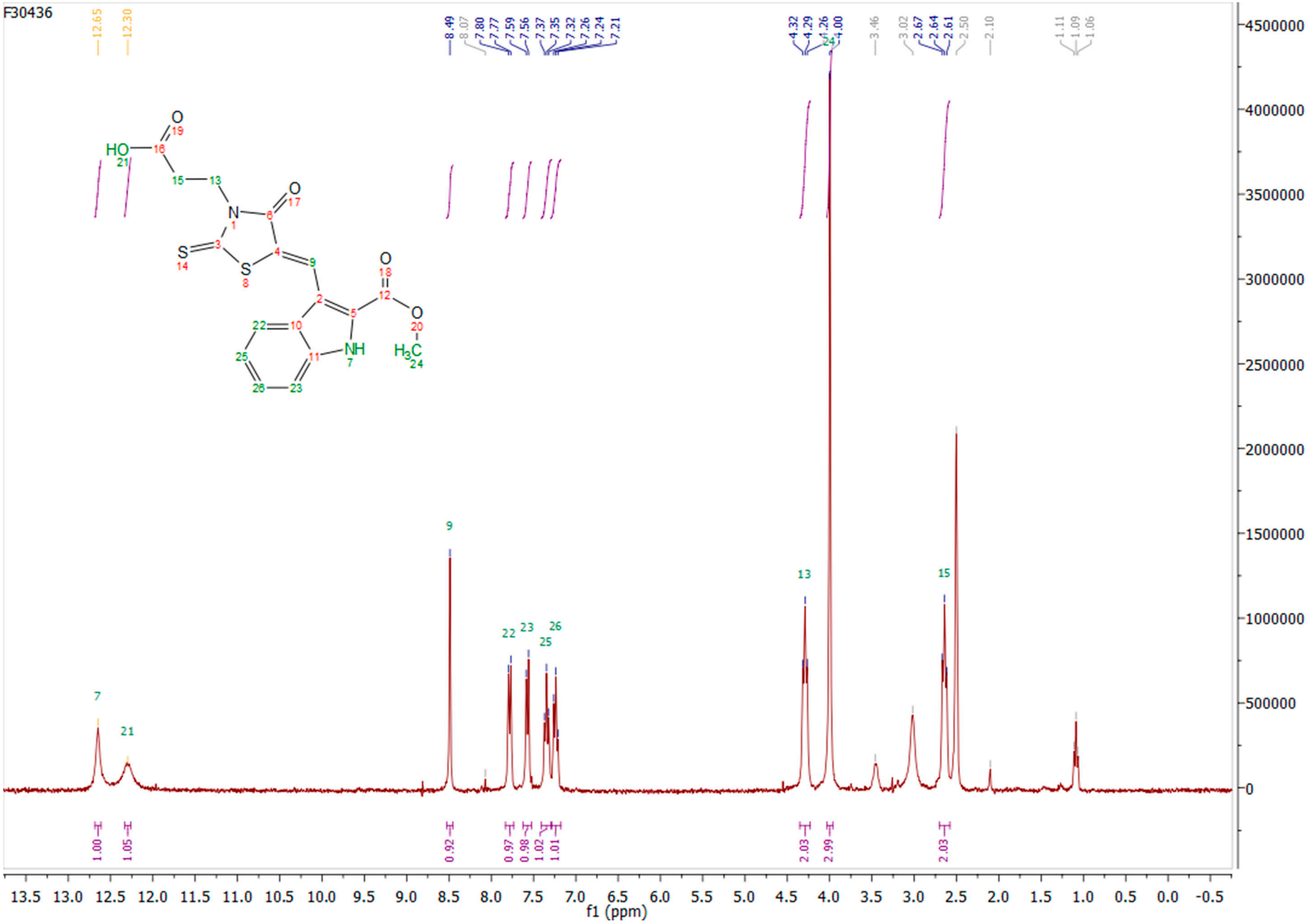
F30436
12.65
12.30
8.49
8.07
7.80
7.77
7.59
7.56
7.37
7.35
7.32
7.26
7.24
7.21
4.32
4.29
4.26
4.00
3.46
3.02
2.67
2.64
2.61
2.50
2.10
1.11
1.09
1.06
HO
O
N
O
S
S
O
O
NH
H_3C
7
21
9
22
23
25
26
13
24
15
1.00
1.05
0.92
0.97
0.98
1.02
1.01
2.03
2.99
2.03
4500000
4000000
3500000
3000000
2500000
2000000
1500000
1000000
500000
0
13.5 13.0 12.5 12.0 11.5 11.0 10.5 10.0 9.5 9.0 8.5 8.0 7.5 7.0 6.5 6.0 5.5 5.0 4.5 4.0 3.5 3.0 2.5 2.0 1.5 1.0 0.5 0.0 -0.5
f1 (ppm)

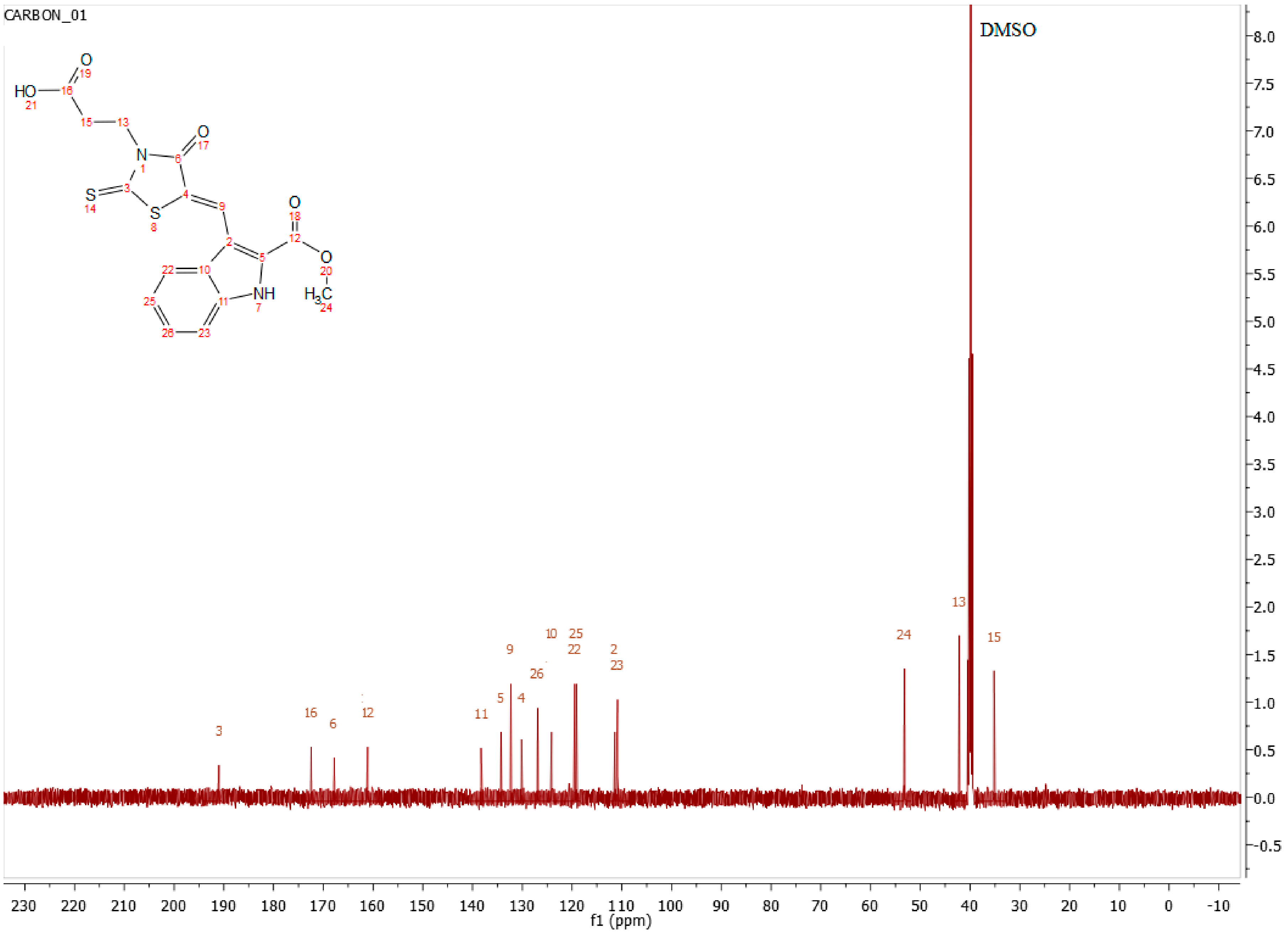
CARBON_01
DMSO
3
16
6
12
11
5
9
4
26
10
25
22
2
23
24
13
15
f1 (ppm)
HO
O
N
S
NH
H3C
